# Single-cell transcriptome analysis identifies distinct cell types and niche signaling in a primary gastric organoid model

**DOI:** 10.1038/s41598-019-40809-x

**Published:** 2019-03-14

**Authors:** Jiamin Chen, Billy T. Lau, Noemi Andor, Susan M. Grimes, Christine Handy, Christina Wood-Bouwens, Hanlee P. Ji

**Affiliations:** 1 Division of Oncology, Department of Medicine, Stanford University School of Medicine, Stanford, CA USA; 2 Stanford Genome Technology Center, Stanford University School of Medicine, Stanford, CA USA

## Abstract

The diverse cellular milieu of the gastric tissue microenvironment plays a critical role in normal tissue homeostasis and tumor development. However, few cell culture model can recapitulate the tissue microenvironment and intercellular signaling *in vitro*. We used a primary tissue culture system to generate a murine p53 null gastric tissue model containing both epithelium and mesenchymal stroma. To characterize the microenvironment and niche signaling, we used single cell RNA sequencing (scRNA-Seq) to determine the transcriptomes of 4,391 individual cells. Based on specific markers, we identified epithelial cells, fibroblasts and macrophages in initial tissue explants during organoid formation. The majority of macrophages were polarized towards wound healing and tumor promotion M2-type. During the course of time, the organoids maintained both epithelial and fibroblast lineages with the features of immature mouse gastric stomach. We detected a subset of cells in both lineages expressing *Lgr5*, one of the stem cell markers. We examined the lineage-specific Wnt signaling activation, and identified that *Rspo3* was specifically expressed in the fibroblast lineage, providing an endogenous source of the R-spondin to activate Wnt signaling. Our studies demonstrate that this primary tissue culture system enables one to study gastric tissue niche signaling and immune response *in vitro*.

## Introduction

Within the microenvironment of the stomach tissue, gastric epithelial cells have complex interactions with other cell lineages. These components include fibroblasts, immune lineages, endothelial cells, and extracellular matrix^1^. This complex cellular milieu plays a critical role in maintaining tissue function and integrity. Similarly, solid tumor development leads to changes in the cellular microenvironment – the interactions between cancer cells and tumor stroma influence tumor development, facilitate metastasis, enable evasion of immune surveillance, and alter therapeutic responses^2^. Despite its important role in tissue regulation and maintenance, very little is known about the cellular features and intercellular communication among gastric epithelial and stromal cells.

Developments in primary tissue culture techniques enable gastric cells to grow and organize into three dimensional structures that resembles miniature organs, referred to as organoids. These primary tissue cultures can be derived from primary gastric tissues^3,4^, epithelial stem cells^5^, or induced pluripotent stem cells^6^. They are capable of self-renewal and self-organization. For this study, organoids are derived from the primary mouse gastric tissues grown in an air-liquid interphase **(ALI) system**^4,7^. The ALI system can be applied to maintain gastrointestinal primary tissue cultures over long periods of time as well as develop engineered cancer models where driver mutations can be systematically introduced^8–10^. This method of primary tissue culture has significant advantages compare to the conventional approaches. First, it recapitulates features of organ structure, maintains multi-lineage differentiation from the primary tissue and has the ability of self-renewal^11^. Second, one can use this method to introduce specific cancer driver events into a wild-type background, thus modeling the progression of specific oncogenes or tumor suppressors. Third, ALI-grown gastrointestinal organoids do not require supplementation of exogenous Wnt growth factors such as Wnt3A and Rspo1^8,10^. Rather, these primary tissue cultures are self-sustaining, suggesting that the medley of different cell lineages provides an endogenous source of niche factors. Therefore, this organoid culture system presents a model system to study the intercellular signaling between epithelial cells and their stroma *in vitro*.

Using a droplet-based single cell RNA sequencing **(scRNA-Seq)** technology^12–14^, we analyzed thousands of individual cells and defined the cellular heterogeneity at single cell resolution in a self-sustaining p53 null gastric organoid model. At the granularity of single cell analysis, one can derive new insights into tissue cellular heterogeneity, identify the characteristics of the diverse cell types in the local microenvironment and discover signaling interactions among different cell populations. For this study of the microenvironment in the ALI model, we identified different cellular lineages, determined the gene differential expression among the major cell types, and as a result identified growth factors that enable signaling crosstalk and thus maintain microenvironment cellular diversity. We found majority of macrophages present in the tissue explants are polarized to wound healing M2-type. We measured the expression of different Wnt signaling genes and determined that the growth factor, *Rspo3* demonstrated highly specific expression in mesenchymal-derived fibroblasts. Our results point to the role of R-spondin, as provided by the cellular microenvironment, being important for maintaining gastric organoid epithelial cell populations in the ALI system.

## Results

### Single cell transcriptional profiling of primary gastric tissues and organoid cultures

**Figure 1:**
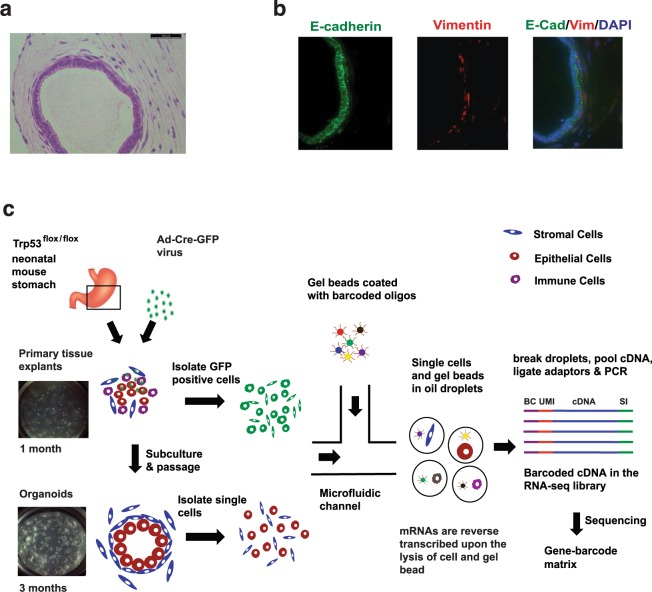
Analysis of gastric organoid populations with immunofluorescence and single cell RNA-Seq. (**a**) H&E staining shows that the Trp53−/− organoid, cultured for three months, consist a layer of tall columnar epithelial cells with an outer lining of spindle-shaped fibroblastic stroma cells. (**b**) The IF showed that E-cadherin (E-cad) is expressed in epithelial cell layer (green) and Vimentin (Vim) is expressed in surrounding fibroblast cells (red). Nuclei are counterstained with DAPI (blue). (**c**) Overview of dissecting cell population using single cell RNA sequencing (scRNA-Seq). Individual cells were encapsulated with single gel beads coated with oligonucleotides in droplet partitions via a high throughput microfluidic device. mRNAs were reverse transcribed by the barcoded oligonucleotides in individual droplets. Subsequently, the droplets were broken and barcoded cDNAs were pooled together for PCR amplification to generate complete scRNA-Seq libraries for sequencing. BC - Barcode, UMI - Unique Molecular Identifier, SI - Sample Index.

We used a p53 null gastric tissue model derived from neonatal *Trp53*^flox/flox^ mice. Wild-type gastric organoids maintained in the ALI environment grow for only a limited time, typically 30 days and are unable to be passaged^15^. We found that inactivation of *Trp53*, the mouse homology of *TP53*, enabled long-term culture and passages of gastric organoids in ALI environment. Moreover, *TP53* loss of function is an early oncogenic transformation event, thus this model replicated the initiation of gastric cancer development^8^. The *Trp53*^*−/−*^ gastric organoids underwent serial passages (passage > 3) and were stably grown for more than 20 weeks. Importantly, the *Trp53*^*−/−*^ organoid consisted of an epithelial layer with surrounding fibroblastic stroma **(**Fig. 1a**)**, which was confirmed by E-cadherin and Vimentin immunofluorescence **(**Fig. 1b**)**. The Cre-mediated *Trp53* deletion in gastric organoids was confirmed by genotyping **(**Additional file 1: Fig. S1**)**. We validated the loss of Trp53 expression with immunofluorescence **(IF)** and western blotting **(**Figs S2–3**)**.

Our experimental design is outlined in Fig. 1c. With scRNA-Seq, we first evaluated single *Trp53*^*−/−*^ cells presented *Trp53*^*flox/flox*^ mouse gastric tissue explants that were cultured in ALI for one month (passage = 0). We sorted out GFP positive cells (~9%) to ensure all sequenced cells were infected with Ad-Cre viruses and thus were *Trp53*^*−/−*^. Second, we isolated and analyzed single cells from *Trp53*^*−/−*^ gastric organoids that have been cultured for three months **(**passage = 4, Figs 1c and S4**)**. The scRNA-Seq library preparation occurred as follows: individual cells were encapsulated with single gel beads coated with oligonucleotides in droplet partitions via a high throughput microfluidic device^13^. Each oligonucleotide is consisted of a 30nt poly-A primer, a 14nt cell barcode, and a 10nt random sequence as unique molecular identifier **(UMI)** to eliminate molecular duplicates and enable single molecule transcript counting. Upon the lysis of cell and gel bead, mRNAs were reverse transcribed by the barcoded oligonucleotides in individual droplets. Subsequently, the droplets were broken and barcoded cDNAs were pooled together for PCR amplification to generate complete scRNA-Seq libraries for sequencing.

In total, we sequenced 4,391 cells from two samples: (1) 2,304 cells selected based on GFP signal from the initial *Trp53*^*−/−*^ gastric tissue explants; (2) 2,087 cells from the stable *Trp53*^*−/−*^ organoid culture **(**Table 1**)**. To ensure that an adequate number of mRNA transcripts were sequenced, we generated more than 200 million reads for each sample, and more than 90,000 reads per cell. A previous study has shown that 50,000 reads per cell is sufficient for accurate cell-type classification and biomarker identification^16^. Approximately 78% and 69.9% of reads were mapped to exonic regions while 4% and 6.6% of reads were mapped to intronic regions in the tissue explants and organoids, respectively. The median number of genes and mRNA transcripts (UMI counts) detected per cell were higher in the gastric tissue explants (~2,900 and ~11,000) compared to the cells from the organoid samples (~2,100 and ~5,800) **(**Table 1 and Fig. S5**)**.

**Table 1:** Sequencing metrics for gastric organoid microenvironment analysis.

Samples	Tissue explants	Organoids
Total Cell Number Sequenced	2,304	2,087
Total Reads	218,223,677	203,273,762
Mean Reads/Cell	94,715	97,399
Median Genes/Cell	2,969	2,090
Median UMI Counts/Cell	11,986	5,802
Total Cell Number Analyzed	2,232	1,961

We used the program Seurat to analyze the scRNA-Seq data^17^. Using best practices per Seurat, our quality control metrics were such that we evaluated genes that were expressed in three cells or more and included those cells that had 500 gene transcripts or more. To remove potential cell doublets where two cells exist within a single droplet partition as well as low quality cells with poor RNA-Seq data^18^, we filtered out those cells that have unique gene counts over 5,000 or contain more than 5% the percentage of mitochondrial genes. Principal component analysis **(PCA)** was performed on remaining cells to reduce the dimensionality of the scRNA-Seq data matrix using high variable genes^14^. Subsequently, cells were clustered based on a graph-based clustering approach^19,20^ and were visualized in two dimensional space using t-distributed Stochastic Neighbor Embedding **(tSNE)**^21^.

### Single cell characterization of primary gastric tissue explants and the microenvironment

**Figure 2:**
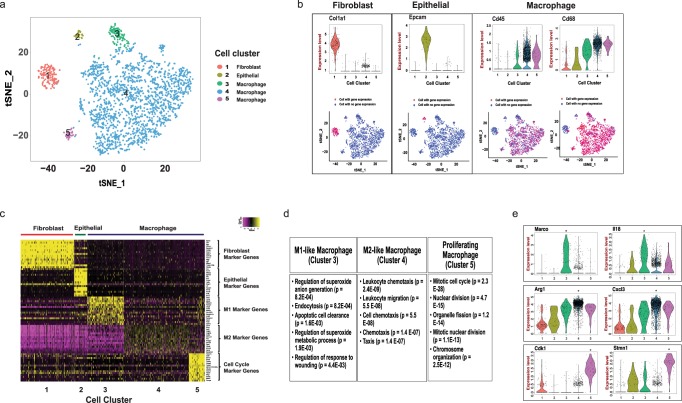
Distinct cell types in the tissue explants. (**a**) A tSNE projection of 2,232 cells from tissue explants. Cells are grouped into five clusters based on transcriptome profiles and are colored accordingly. The cell type assignment of each cluster is based on the gene expression analysis. (**b**) The expression of cluster specific genes, i.e., *Col1a1*, *Epcam*, *Cd45*, *Cd68*, are displayed on violin plots and tSNE maps. (**c**) Heatmap shows the expression of top 15 cluster specific genes in 200 cells (or less) from each cluster. Yellow - high expression. Purple - low/no expression. Each row represents a gene, and each column represents a single cell. (**d**) The top five GO biological processes in macrophage clusters based on the gene enrichment analysis. Top 50 genes in each cluster were included in the analysis. *p* value was calculated with the Fisher’s Exact Test and adjusted by the Bonferroni correction^24^. (**e**) The violin plots of macrophage group specific genes, i.e., *Marco*, *IL18*, *Arg1*, *Cxcl3*, *Cdk1*, *Stmn1*. Every dot represents an individual cell in both violin plots and tSNE maps. The gene expression level is the natural log of the normalized UMI counts in the violin plot. *Bonferroni adjusted *p* value < 0.001.

Our analysis indicated that ALI-based primary gastric tissue explants underwent macrophage-based tissue remodeling. Five cellular clusters were apparent on the tSNE map **(**Fig. 2a**)**. Cell lineage markers revealed three cell types in the primary tissues **(**Fig. 2b**)**: fibroblast cells (cluster 1), epithelial cells (cluster 2), and macrophages (clusters 3–5). Fibroblast specific genes, such as *Col1A1*, *Bgn*, *Dcn*^22^, were significantly expressed in the cluster 1, while epithelial specific markers, such as *Epcam*, *Krt7* and *Krt14*^13^, were significantly expressed in cluster 2. Most of the cells in clusters 3–5 expressed leukocyte common antigen *Cd45* (*Ptprc*). Moreover, the expression of *Cd68*, *Cd11b (Itgam)*, *and Cd18* (*Itgb2*) in clusters 3–5 indicated that these cells were tissue macrophages^23^.

We identified cluster specific genes by comparing each cluster of cells to all other cell clusters using differentially expression analysis **(**Fig. 2c and Table S1**)**. To evaluate the three macrophage clusters, we performed gene ontology **(GO)** enrichment analysis on cluster specific genes using the EnrichR program^24^, and identified the top ranked pathways **(**Fig. 2d**)**. There were distinct gene expression patterns among the macrophages: cluster 3 was enriched with the inflammatory responses and cell clearance related genes, cluster 4 was enriched with the leukocyte cytokine and migration genes, and cluster 5 was enriched with cell cycle genes. Our results suggested that a subgroup (~6%) of the macrophages (cluster 3), expressing scavenger receptor *Marco* and cytokine *Il18* **(**Fig. 2e**)**^25^, and thus, had inflammatory characteristics consistent with what is described as the M1 class. Cluster 4 demonstrated an upregulation of genes associated such as *Arg1* (anti-inflammatory), *Cxcl3* (pro-angiogenic), *Mmp12* (extracellular matrix remodeling) **(**Fig. 2e**)**^26,27^. These genes are often upregulated in tumor-activated macrophages **(TAMs)**, also known as M2 macrophages, a category of immune cells that play a critical role in tissue remodeling. Cell cycle genes, such as *Cdk1*, *Aurkb* and *Stmn1* were significantly upregulated in cluster 5 **(**Fig. 2e**)**, indicating a subgroup (~2%) of macrophages was actively proliferating. This finding agrees with previous studies where it was observed that tissue macrophages undergo cell division within the primary tissue where they reside^28^. To evaluate the reproducibility of our finding, we sequenced and analyzed a separately prepared single cell library from an independent biological replicate. Based on this analysis, we confirmed that epithelial, fibroblast and macrophage are the three main cell populations in ALI cultured tissue explants with cell type specific markers in distinct cell clusters **(**Fig. S6**)**.

Overall, fewer epithelial cells and fibroblast cells were detected compared to macrophages. The enrichment of tissue-resident macrophages may be due to the selection of single cells using GFP signals from ALI-based primary gastric tissue explants. The Ad-Cre-GFP viruses infected the cells in tissue explants were isolated based on GFP signals after cultured in ALI for 1 month. We speculated that the GFP signals were more likely to remain in the non-proliferating, long half-life tissue-resident macrophages than other cell lineages. Our data suggested that the growth condition of ALI favors macrophages during the initial tissue explants remodeling phase, which provides a potential platform for future studies of macrophages *ex vivo*.

### Cellular lineages in the gastric organoids and their microenvironment

**Figure 3:**
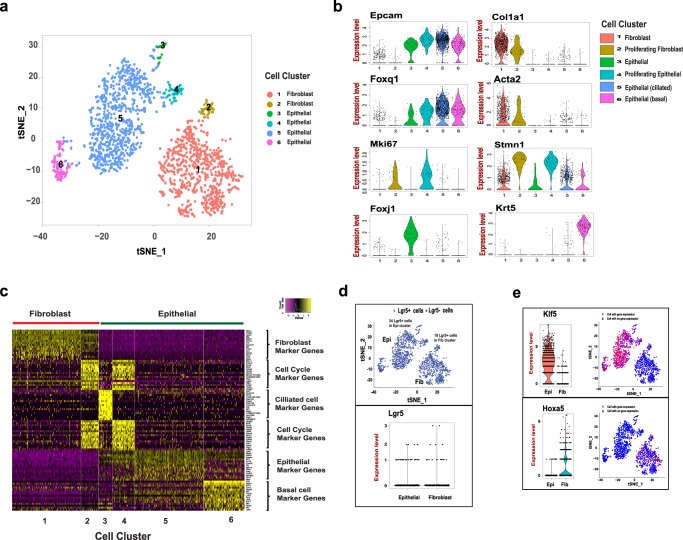
Organoids are composed of epithelial and fibroblast cell types. (**a**) tSNE projection of 1961 cells from gastric organoids. Cells are grouped into six clusters based on transcriptome profiles and were colored accordingly. (**b**) The expression of cell type specific genes, *Epcam*, *Col1a1*, *Foxq1*, *Acta2*, *Mki67*, *Stmn1*, *Foxj1*, and* Krt5* are displayed on violin plots and tSNE projection. The expression level of violin plot is the natural log of the normalized UMI counts. (**c**) The heatmap of top 15 cluster specific genes in 200 cells (or less) from each cluster. Yellow - high expression. Purple - low/no expression. Each row represents a gene, and each column represents a single cell. (**d**) Stem cell marker Lgr5 is expressed in both epithelial and fibroblast cells. *Lgr5* mRNA is detected in both epithelial and fibroblast cell clusters on the tSNE map (top). Raw *Lgr5* counts in Epithelial and Fibroblast cell clusters (bottom). (**e**) The expression of lineage specific transcriptional factors, *Klf5* (top) and *Hoxa5* (bottom), on violin plots and tSNE maps. Epi – Epithelial, Fib – Fibroblast. The expression level of violin plot is the raw UMI counts * Bonferroni adjusted *p* value < 0.001.

Next, we identified multiple cellular lineages from gastric organoid cultures maintained over three months and having undergone four passages. From this extended primary tissue culture sample, we sequenced 2,087 cells from the gastric organoid culture and characterized a total of 1,961 cells that had adequate sequencing quality. Two cell types, epithelial and mesenchymal-derived fibroblast, were clearly denoted based on gene expression patterns and cell clustering on tSNE map **(**Fig. 3a,b**)**. Similar to the gastric explants, the epithelial cell type was enriched with markers, such as *Epcam*, *Krt7* and *Krt14*, and the fibroblast type was enriched with lineage-specific markers, such as *Col1a1*, *Bgn* and *Dcn*. The lack of expression of intestinal lineage markers, such as *Muc2* and *Cdx1*, indicating that all of these cells were strictly of gastric origin. Notably, cells from the primary organoid cultures did not express leukocyte lineage markers, indicating macrophages could not be continuously passaged despite their initial dominant presence in the ALI environment.

We defined the heterogeneity within the epithelial lineage and mesenchymal-fibroblast lineage, and identified cluster specific genes **(**Fig. 3b,c and Table S2**)**. Based on the transcriptome profiles, two subpopulations were identified within the fibroblast lineages **(cluster 1 and 2)** and four subpopulations were identified within the epithelial lineages **(cluster 3**, **4**, **5 and 6)**. The majority of epithelial cells (>85%) were grouped in cluster 5, expressing gastric mucin-producing lineage specific transcriptional factor *Foxq1*^29^. The majority of mesenchymal fibroblast cells (>90%) were grouped in cluster 1, expressing myofibroblast markers *Acta2* and *Postn*. Fibroblast cells in cluster 2 and epithelial cells in cluster 4, accounting for ~6% of respective cell lineage, were enriched with cell cycle genes, such as *Mki67* and *Stmn1*. This is an indication of the proliferating cells in each cell lineages. In cluster 3, epithelial cells were enriched with transcriptional factors *Foxj* and *Mlf1*, suggesting presence of a small population of ciliated epithelial cells^30^. Epithelial cells in cluster 6 specifically expressed *Krt5* and *Krt14*, suggesting they are basal epithelial cells^31^. Basal epithelial cells normally present in esophageal and forestomach^31^. Therefore, basal cells may be derived from the non-glandular forestomach during tissue dissection.

Studies have shown that single Lgr5+ cells were capable of replenishing gastric cells, and thus is one of gastric stem cell markers^11^. Neonatal Lgr5+ cells were reported to contribute to formation of the mature gland in both the pylorus and corpus^3^. More recently, Barker and colleagues reported Lgr5 expression in a subpopulation of chief cells in mouse corpus glands, driving epithelial regeneration and maintaining the homeostatic stem cell pool^32^. We examined the Lgr5 expression in the organoid cell population. Despite low count numbers, we found that ~3% of cells (34 cells) in the epithelial cluster **(**Fig. 3d) and ~2% of cells (18 cells) in the fibroblast cluster expressing *Lgr5* mRNA **(**Fig. 3d**)**. To further characterize the heterogeneity between Lgr5+ cell lineages, we selected and generated the Lgr5+ only cell clusters with epithelial (Lgr5_1) and fibroblast (Lgr5_2) lineages from the respective clusters **(**Fig. 3d**)**. Per single cell gene expression analysis, epithelial- and fibroblast-specific genes distinguished the two Lgr5+ groups. For example, *Epcam* and *Dcn* were significantly upregulated in Lgr_1 cells, while *Col1a1* and *Krt14* were significantly upregulated in Lgr5_2 cells **(**Fig. S7 and Table S3**)**. Our data showed that both epithelial and fibroblast lineage cells express *Lgr5* mRNA in this p53 null organoid model.

We found the development status of organoids resembled their tissue of origin, namely immature stomach tissue. We did not detect lineage specific markers that usually present in differentiated gastric epithelial cells, including *Muc5ac* (pit mucous cells), *Muc6* (gland mucous cells), *Atp4b* (parietal cells), and *Pgc* (Chief cells). We detected a small number of endocrine cells that express ghrelin (*Ghrl*). The epithelial cells expressed transcriptional factors *Klf5* and *Sox2* **(**Figs 3d and S8**)**, which are typically expressed in progenitor cells in the immature epithelium of the stomach^6^. Similarly, the fibroblast cells expressed *Hoxa5* and *Sox9* **(**Figs 3d and S8**)**, two transcriptional factors that are specifically expressed the undifferentiated cells in the mesenchyme of immature stomach^33^. Overall, these results suggest that these *Trp53* null gastric organoids retain cellular properties of the mouse stomach.

### Distinct Wnt/beta-catenin signaling activation between the cellular lineages

Wnt/b-catenin signaling pathways regulates the homeostasis of gastrointestinal tract and maintain the self-renewal capability of epithelial stem cells^34^. For example, loss of Wnt signaling, due to gastric specific inactivation of Wnt receptor frizzled 7 (Fzd7), is deleterious to mouse gastric epithelium and triggers rapid epithelial repopulation^35^. Many methods for primary tissue cultures only maintain pure epithelial cells. To sustain their growth in tissue culture, these isolated epithelial cells require external supplementation of niche factors such as Rspo1, Wnt3A, Noggin^3^ or external fibroblasts as feeder cells^36^. Given that ALI-maintained, multi-lineage organoid cultures did not require external supplementation, we hypothesized that secretion of endogenous niche factors by fibroblast cells in stroma sustained Wnt/β-catenin signaling and stem-cell renewal in organoids.

**Figure 4:**
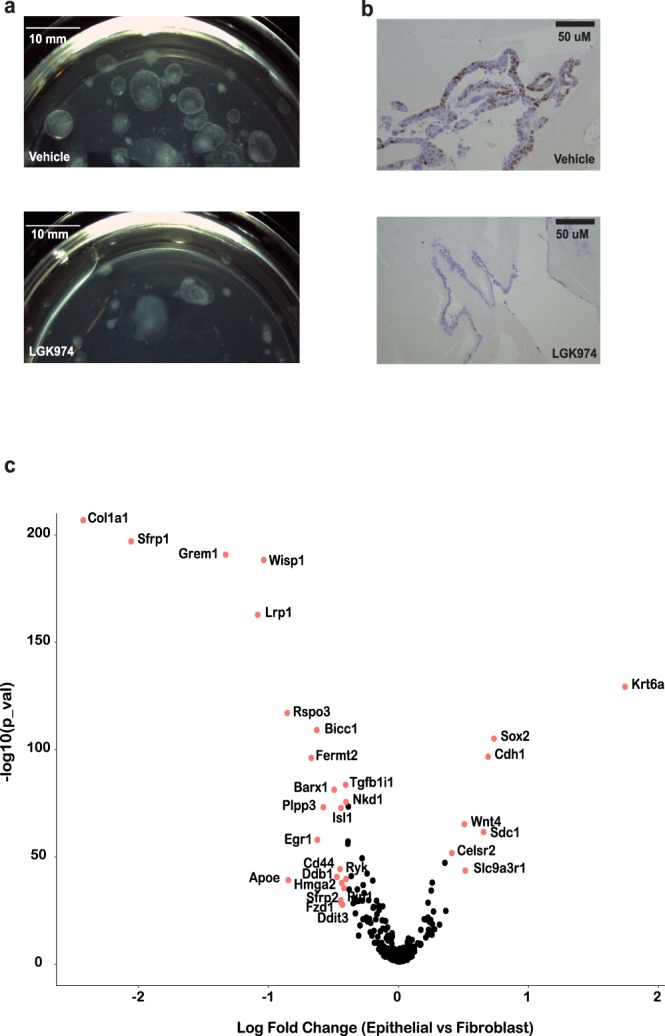
Wnt signaling regulates the growth of gastric organoids in air-liquid interface. The growth of organoids were examined using stereoscope (**a**) and Ki67 staining (**b**) at Day 21 after treating with 10 uM LGK974 for 7 days. 0.1% DMSO in organoid growth media as the vehicle control. (**c**) Differentially expressed Wnt related genes between epithelial and fibroblast cells. Genes with fold change >1.5 (|Ln| > 0.4) and Bonferoni adjusted  *p* < 0.05 were labeled in red with gene name. The x-axis is the natural log transformed fold change of gene expression in epithelial vs mesenchymal cells. The y-axis is the −log10 transformed *p* value.

To examine the effect of Wnt inhibition, we treated the gastric organoids with the Wnt signaling inhibitor LGK974. This molecule targets Porcupine, a Wnt-specific acyltransferase, and thus inhibits Wnt signaling both *in vitro* and *in vivo*. Previous studies have shown that inhibition of Wnt signaling leads to cessation of epithelial cell proliferation and loss of crypt in mouse as well as in organoids derived from primary gastric and intestinal tissues^4,35,37,38^. After LGK974 treatment, the organoid epithelial layer degenerated and there was notable growth inhibition as observed with Ki67 staining **(**Fig. 4a,b**)**. These results indicated that this gastric organoid model, despite p53 inactivation, recapitulated the *Wnt* niche dependency *in vitro*.

We examined our results from scRNA-Seq to define the Wnt dependencies between epithelial and fibroblast cell types. Among Wnt signaling pathway genes from Mouse Genome Informatics (http://www.informatics.jax.org/, GO:0016055), 330 were expressed among the organoid cells **(**Table S4**)**. Thirty genes were differentially expressed with statistical significance (bonferroni corrected *p* < 0.05 and fold change >1.5) between the epithelial and stromal fibroblast cell populations **(**Fig. 4c**)**. Differentially expressed genes included well-known lineage specific markers, i.e. upregulation of *Col1a1* and *Grem1* in fibroblast cells as well as upregulation of *Krt6a* and *Cdh1* in epithelial cells. The expression of transcription factor *Barx1* is restricted to stomach mesenchyme during gut organogenesis^39^. Barx1 signals through Sfrp1 and Sfrp2 to control stomach-specific epithelial differentiation. The upregulation of *Barx1*, *Sfrp1*, and *Sfrp2* in organoid fibroblast cells further supported that ALI based self-sustain p53 null gastric organoids retain properties of the mouse stomach.

### Lineage-specific expression of *Rspo3* and *Wnt4* crosstalk

Wnt proteins bind to a variety of different receptors that include FZD, LRP5 and LRP6. This interaction leads to β-catenin nuclear translocation and Wnt/b-catenin activation. Similarly, the family of R-spondin growth factors (RSPO1–4) interact with LGR4-LGR6 receptors, inhibit the degradation of FZD and potently activate Wnt/b-catenin signaling^40^. However, Wnt and R-spondin proteins are not equivalent in terms of their function^36^. Unlike R-spondin ligands, Wnt proteins cannot induce Lgr5+ stem cell self-renewal, but instead prime the expression of R-spondin receptors. Given the differential expression of Wnt genes among the various lineages, we examined gene expression for secretory factors between the two major cell populations of the gastric cancer precursory cultures.

**Figure 5:**
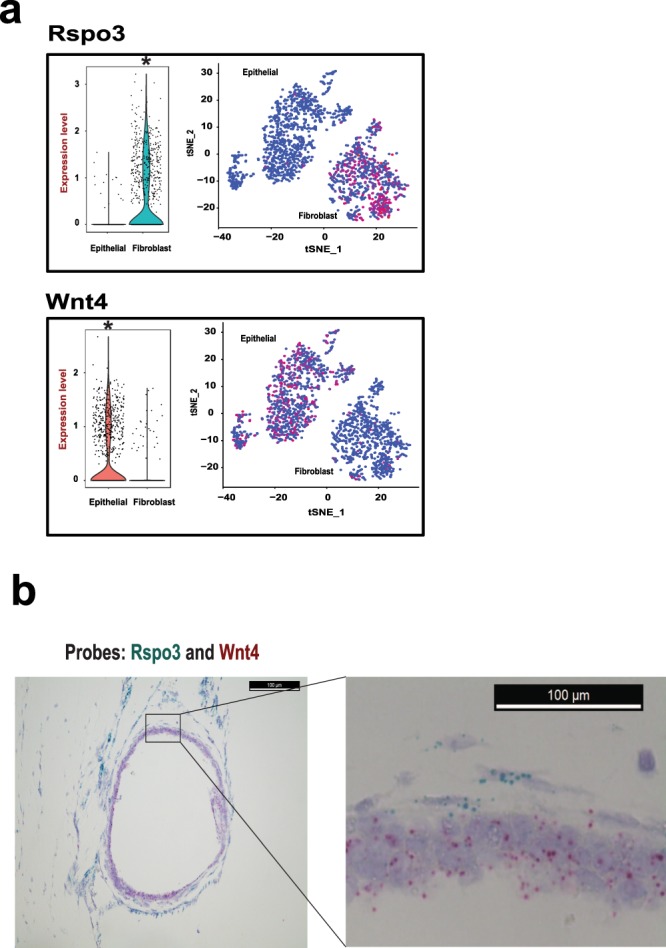
Cell type specific expression of Rspo3 and Wnt4. (**a**) Cell type specific expression of Wnt ligands. The violin and tSNE plots depict that *Rspo3* mRNA expression is enriched in the fibroblast cells and *Wnt4* mRNA expression is enriched in the epithelial cells. The expression level of violin plot is the natural log of the normalized UMI counts. *Bonferroni adjusted *p* value < 0.001. (**b**) RNA *In situ* hybridization confirms cell type specific expression of *Rspo3* and *Wnt4* in the organoid. The organoid FFPE section is hybridized and amplified by the probes specifically targeting *Rspo3* and *Wnt4* mRNAs. Rspo3 probes are the blue dots detected in the fibroblast cells surrounding the organoid, and Wnt4 probes are the red dots detected in the epithelial cells forming the wall of the organoid sphere (Scale bar = 100uM).

Based upon single cell analysis, we found that the gene expression of *Rspo3* was significantly enriched in the fibroblast cells (Adjusted *p* value = 7.6E-126), while the gene expression of *Wnt4* was significantly enriched in the epithelial cells with an adjusted *p* value = 7.1E-84 **(**Fig. 5a**)**. For the initial gastric tissue explants, we observed specific expression of *Rspo3* in the fibroblast cell. However, the expression of *Wnt4* was very low in the epithelial cells (Fig. S9).

We confirmed that the *Rspo3* gene was specifically expressed in the fibroblast cells and *Wnt4* was specifically expressed in the epithelial cells. As an orthogonal validation, we applied RNA *in situ* hybridization **(ISH)** to directly examine mRNA expression in organoid cells. First, we evaluated the sensitivity and specificity of the RNA ISH with two positive controls. Specifically, we measured the expression of two housekeeping genes *Ppib* and *Polr2a* and found that they were simultaneously expressed in all organoids cells while there was no expression of a negative control, the E. coli gene *dapB* gene **(**Fig. S10**)**. Using gene-specific probes, we confirmed that *Wnt4* mRNA was specifically expressed in the epithelial cellular layers making up the cystic portion of the organoid. In contrast, the *Rspo3* mRNA was specifically expressed in the adjacent lining composed of fibroblast cells **(**Fig. 5b**)**. Overall, we confirmed the lineage specificity of expression based on ISH localization of the *Wnt4* and *Rspo3* transcripts. Most recently, Sigal and colleagues reported that endogenous stroma-derived Rspo3 controls gastric epithelial stem cells and gland homeostasis using mouse gastric tissues^41^. They demonstrated lineage specific expression of Rspo3 in gastric myofibroblasts and of Wnt ligands in epithelial cells, which agreed with our findings. Our results implicated that *Rspo3* and *Wnt4* are endogenous Wnt growth factors mediating intercellular communication in ALI based gastric tissue culture.

## Discussion

To study the epithelial-stromal interaction *in vitro*, we applied a high-throughput single cell RNA-Seq in a gastric organoid model that is composed of epithelial cell and stromal components. Unlike the epithelial only organoids, this model does not require supplementation of exogenous Wnt growth factors such as Wnt3A and Rspo1, thus providing a platform to study the endogenous growth factors maintaining the growth of organoids. Wild-type gastric tissue explants can be cultured in ALI and form organoids up to weeks. However, we and others have determined that inactivation of p53 is the minimum requirement for the long-term culture of gastric organoids in ALI^8^. *Trp53*, which encodes p53, is the most frequently mutated genes in gastric cancer^42^. A recent study demonstrated that loss of p53 enhanced the proliferative and self-renewal abilities of gastric epithelial cells^43^. Importantly, gastric epithelial organoid with TP53 inactivation remained dependent on R-spondin^44^.

To dissect the cell populations, we profiled the transcriptomes of 4,391 individual cells from initial gastric tissue explants as well as stably cultured organoids. Previous IHC and IF studies on ALI based organoid culture identified epithelial and stromal fibroblast cells^8,15^. In this study, we demonstrated that macrophage were present in the early stroma as well. However, only epithelial and fibroblast cell types were present in organoids cultured for three months, suggesting that macrophages cannot be maintained over time after repeated passages in ALI. We noticed that macrophages were selectively enriched due to selection of GFP positive cells from the tissue explants cultured in ALI for 1 month. We speculate that the growth condition of ALI favors macrophages with p53 inactivation to retain the GFP signals compared to other cell types during the initial tissue explants remodeling phase. However, this bias provided an opportunity to study macrophages in the tissue explants. Studies have shown that p53 attenuates both M1 inflammatory responses and alternative M2 polarization of macrophages^45,46^. During the preparation of this manuscript, Neal *et al*. reported the presence of M2-type macrophages in the ALI based culture from a patient derived clear cell renal carcinoma organoids^47^. In this study, we found that majority of macrophages with p53 inactivation resembled tumor-associated M2-like status and only a small population was similar to inflammatory M1-like. This result suggests that *Trp53*^*−/−*^ macrophages in the initial tissue explant microenvironment were prone to polarize towards wound healing and tumor promotion.

In organoids cultured for three months, we defined heterogeneous epithelial cell subpopulation and identified a small subpopulation of ciliated and basal epithelial cells. Normally, ciliated and basal epithelial cell types were not present in the mouse gastric tissues. Gastric stem cells were reported to be able to adopt various aberrant differentiation patterns, resulting, in rare instances, in cells with ciliated features^48^. However, a previous study reported that the presence of ciliated cells in stomachs of mice lacking the gastric H,K-ATPase alpha -subunit, suggesting loss of functional parietal cells would lead to the development of ciliated cells. We were unable to detect any parietal cells in our organoid culture, which may also contribute to the development of ciliated cells. Basal epithelial cells normally present in the non-glandular forestomach^31^. Therefore, we cannot exclude the possibility of basal cell contamination from the non-glandular forestomach during tissue preparation.

The ALI organoids had the properties resembling the neonatal stomach based on transcriptional factors. We were able to detected the presence of stem cell marker *Lgr5* in a subset of cell populations, albeit very low counts. Cluster analysis suggested that cells expressing *Lgr5* mRNA were present in both epithelial and fibroblast cell types. A previous study observed *Lgr5* mRNA expression among wild-type gastric epithelial cells, but not in the fibroblast cells^49^. However, a recent study found LGR5 was expressed in both epithelium and stromal cells in normal colon tissue, and the expression level was notably increased in tumor samples^50^. Moreover, LGR5 was found to play a role in tumors of mesenchymal origin^51^. Therefore, it is plausible that oncogenic events, such as loss of p53, might lead to the expression of Lgr5 in stromal fibroblasts. Future studies are needed to characterize the potential functional role of Lgr5 in gastric stromal cells.

Importantly, we identified Rspo3 as the endogenous source supplied by fibroblasts in the gastric stroma. Recently, Virshup *et al*. found that Rspo3 to be the predominant Rspos expressed in cultured mouse intestinal stroma and was sufficient to support the intestinal homeostasis^36^. Another group reported that stromal Rspos sustain gastric epithelial stem cells and gland homeostasis^41^. Recurrent *RSPO* fusions were identified in a subset of colon cancers and were mutually exclusive with *APC* mutations, suggesting that they are involved in the activation of Wnt signaling^52^. The *RSPO* fusions probably render epithelial cells to lose the dependency on the *RSPO3* secreted by the fibroblasts. Targeting RSPO3 in colon tumors with RSPO fusions promoted differentiation and loss of stem-cell function^53^. More recently, Sato and colleagues reported that a subset of patient derived gastric organoids were sensitive to a Porcupine inhibitor, suggesting Wnt niche could be a targetable module for a subset of Wnt-dependent human gastric cancers^44^. They also showed that gastric epithelial organoids with either *TP53* or *CDH1* inactivation are RSPO dependent. Together, our studies suggested that fibroblasts are the likely source of Rspos that support the growth niche in gastric tissue homeostasis and early cancer development.

## Materials and Methods

### Organoid development and growth

The Stanford University Administrative Panel on Laboratory Animal Care approved all animal experimental protocols. All the experiments were performed in accordance with relevant guidelines and regulations. *Trp53*^flox/flox^ mice were kindly provided by Dr. Anton Berns (Meuwissen, Linn *et al*. 2003). We dissected corpus and antrum regions of stomachs from neonatal mice (age P4-7) and washed them in cold F12 to remove all luminal contents. The stomach was extensively minced and embedded in collagen gel using a double-dish culture system as previously described^4^. Cre recombinase adenoviruses (Ad-Cre-GFP, Vector Biolabs) were added the cultures to induce *Trp53* deletions in the cultured gastric tissue. The cultures were checked for GFP signaling by fluorescence microscopy after 3 days to confirm the infection. We replace organoid growth media (F-12 nutrient mixture, 20%FBS, 1%Antibiotic-Antimycotic) every week. The organoids were passaged at a 1:2 ratio every three to four weeks. To determine the effects of Wnt inhibition on these organoids, we treated the organoid culture with 10 uM LGK974 (Selleck Chemicals) for a period of 7 days.

### Molecular characterization of mouse gastric organoids

The mouse genomic DNA (gDNA) was extracted from the tail biopsy using the Maxwell® 16 Tissue DNA Purification Kit (Promega), and the organoids gDNA was extracted using QuickExtract™ DNA Extraction Solution (Epicentre). The amount of DNA was quantified by Qubit® dsDNA BR Assay Kit (Invitrogen). The PCR primer sequences used for genotyping: Trp53_Fw: CACAAAAACAGGTTAAACCCAG. Trp53_Rv: AGCACATAGGAGGCAGAGAC. Cdh1_Fw: GGGTCTCACCGTAGTCCTCA. Cdh1_Rv: GATCTTTGGGAGAGCAGTCG. The PCR was performed using the Q5® Hot Start High-Fidelity 2X Master Mix (NEB) according to the manufacturer’s protocol. Briefly, initial denaturation at 98 °C for 30 sec, followed by 30 cycles of amplification (98 °C for 10 sec, 64 °C for 10 sec, 72 °C for 15 sec), and a final extension at 72 °C for 3 min. The PCR products were run on 2% agarose gel.

### Western blotting

Cell lysates (30 μg) were separated on 4–15% precast polyacrylamide gels (Mini-PROTEAN® TGX™ Precast Protein Gels, Bio-Rad) and were transferred to nitrocellulose membranes. The antibodies used for blotting included p53 (Santa Cruz, sc-1311-R) and beta actin (Abcam, ab8227).

### Immunofluorescence and immunohistochemistry studies

The organoid samples were fixed with 4% paraformaldehyde overnight and paraffin-embedded as previously described^4^. Sections (~5 uM) from blocks were deparaffinized and stained with H&E for histology analysis. For IF staining, we used primary antibodies for the following proteins: p53 (1:100, Santa Cruz, sc-1311-R), E-cadherin (1:300, BD Biosciences, 610182) and Vimentin (1:100, Cell signaling, 5741). The secondary antibodies used were Alexa Fluor 488 goat anti-mouse (1:500; Invitrogen, A10680) and Alexa Fluor 555 goat anti-rabbit (1:500; Invitrogen, A21428). The ProLong Gold Antifade Reagent with DAPI (Cell Signaling) was used for mounting. For IHC staining, we used Ki67 (1:200, Cell signaling, 9027), SignalStain® Boost IHC Detection Reagent (Cell signaling, HRP, Rabbit) and SignalStain® DAB Substrate Kit (Cell signaling).

### Organoid disaggregation, single cell library preparation and flow cytometry

The gastric organoids were isolated from collagen gel by incubating with collagenase IV (500 mg/mL, Worthington) in a 15-mL Falcon tube at 37 °C for up to 1 hour. The tube was centrifuged at 400 g for 5 min. The supernatant was discarded and the organoid pellet was washed twice with 10 mL F-12. The organoids were collected by centrifuging and were re-suspended with 500 uL Trypsin-EDTA (0.25%, Gibco) at 37 °C for 25 min to dissociate into single cells. We used a P200 Pipette to pipet the cell suspension up and down several times to ensure digestion. The gastric cells were washed with 5 mL organoid growth media (F-12 nutrient mixture, 20% FBS), and filtered twice through cell strainers (mesh size: 40 uM). After being centrifuged at 400 g for 5 min, the supernatant was discarded, cells were washed with 1X PBS and were re-suspended at ~1000 cells/uL in 1X PBS containing 0.4% BSA. The GFP signal was detectable in tissue explants 1 month after the Ad-Cre-GFP infection and individual cells with positive GFP signal were isolated using a BD Influx cell sorter.

The single cell RNA-Seq libraries were prepared using the 10X Genomics Single Cell 3′ Gel Bead and Library Kit following the manufacturer’s instruction. Briefly, cell suspensions were loaded on 10X Genomics Single Cell Instrument where single cells are partitioned in droplets. Upon encapsulation, cells are lysed and the gel bead dissolution releases sequencing adapter oligonucleotides that mediated the reverse transcription of poly-adenylated RNAs. With the incorporation of the oligonucleotide adapter, each cDNA molecule incorporates a UMI and droplet partition barcode. Subsequently, the emulsions were broken and barcoded cDNAs were pooled for PCR amplification. Amplified cDNAs were shared to ~200 bp using a Covaris E210 system. After end repair and A-tailing, adapters were ligated to the sheared DNA product, followed by PCR to incorporate sample indices. The scRNA-Seq libraries were run on an Agilent High Sensitivity DNA chip for quality control, and were quantified using the KAPA Library Quantification Kits for Illumina® platform (KAPA Biosystems). The libraries were loaded at 20 pM on an Illumina HiSeq 2500 using the TruSeq v3 200 cycles HS kit or at 1.8 pM on an Illumina NextSeq 500 using the 150 cycles High Output kit. Standard Illumina paired-end sequencing with dual indexing uses the following read length: Read 1–98 bp (RNA read), Read 2–10 bp (UMI, tissue explant library only have 5 bp), i7 Index −14 bp (Single cell index), i5 index −8 bp (Sample barcode).

### Data analysis

The Chromium Single Cell Software Suite (http://support.10xgenomics.com/single-cell/software /overview/welcome) was used to demultiplex samples, process barcodes, and count single cell genes^54^. Briefly, FASTQ files were generated from Read 1 (RNA read), Read 2 (UMI) and i7 index (single cell index). Reads were assigned to individual samples after demultiplexing with the i5 index. Read 1 was aligned to mouse reference genome mm10 using STAR^55^. Subsequently, single cell index and UMI were filtered using the Cell Ranger pipeline^54^. The final output was a gene-barcode matrix contains only confidently mapped (MAPQ = 255), non-PCR duplicates with valid barcodes and UMI. Matrices from multiple libraries were combined together by concatenation in the Cell Ranger R Kit (http://support.10xgenomics.com/single-cell/software/pipelines/latest/rkit).

The gene-barcode matrices were further analyzed and visualized based on the Seurat R package^17^. Per Seurat’s recommendation for quality control, the gene expressed less than 3 cells and the cell contained less than 500 genes were discarded. To remove potential cell doublets and low quality cells^18^, the cells that have unique gene counts over 5,000 or the percentage of mitochondrial genes more than 5% were filtered out.

Top variable genes across single cells were identified using the method described in Macosko *et al*.^14^. Briefly, the average expression and dispersion were calculated for each gene, genes were subsequently placed into 20 bins based on expression, and then calculating a z-score for dispersion within each bin. Principal component analysis **(PCA)** was performed to reduce the dimensionality on the log transformed gene-barcode matrices of top variable genes^14^. Cells were clustered based on a graph-based clustering approach^19,20^, and were visualized in 2-dimension using tSNE^21^. Likelihood ratio test that simultaneously test for changes in mean expression and in the percentage of expressed cells was used to identify significantly differentially expressed genes between clusters^56^.

### *In situ* RNA hybridization

*In situ* RNA hybridization was performed on organoid FFPE tissue sections using the RNAscope® 2-plex Reagent Kit (Advanced Cell diagnostics) following the manufacturer’s instructions. Briefly, the FFPE tissue sections were deparaffinized, boiled in the target retrieval reagent for 8 mins and digested by protease for 15 mins, followed by a series of probe hybridization and signal amplification. The slides were counterstained in 50% hematoxylin for 30 sec, mounted with VectaMount (Vector laboratories), and examined under a bright field microscope. The RNAscope probes used were as follows: Mm-Rspo3 (402011), Mm-Wnt4-C2 (401101-C2), RNAscope® 2-plex positive control probe_Mm (320761), and RNAscope® 2-plex negative control probe (320751).

## Supplementary information

Supplementary Information

## Data Availability

The sequencing data from this study were deposited at NCBI Sequence Read Archive (Accession number: SRP104455).
