# Supplementary material for: Single-cell transcriptome analysis identifies distinct cell types and niche signaling in a primary gastric organoid model
Source: Sci Rep. 2019 Mar 14;9:4536. doi: 10.1038/s41598-019-40809-x (PMC6418230; doi:10.1038/s41598-019-40809-x)
Supplement: Supplementary file 1 — Supplementary Information [file 41598_2019_40809_MOESM1_ESM.pdf]

SUPPLEMENTARY INFORMATION

Single-cell transcriptome analysis identifies distinct cell types and niche signaling in a primary gastric organoid model

Jiamin Chen[1], Billy T Lau[2], Noemi Andor[1], Susan M Grimes[2], Christine Handy[1], Christina Wood-Bouwens[1], Hanlee P Ji[1,2]

[1]Division of Oncology, Department of Medicine, Stanford University School of Medicine

[2]Stanford Genome Technology Center, Stanford University School of Medicine

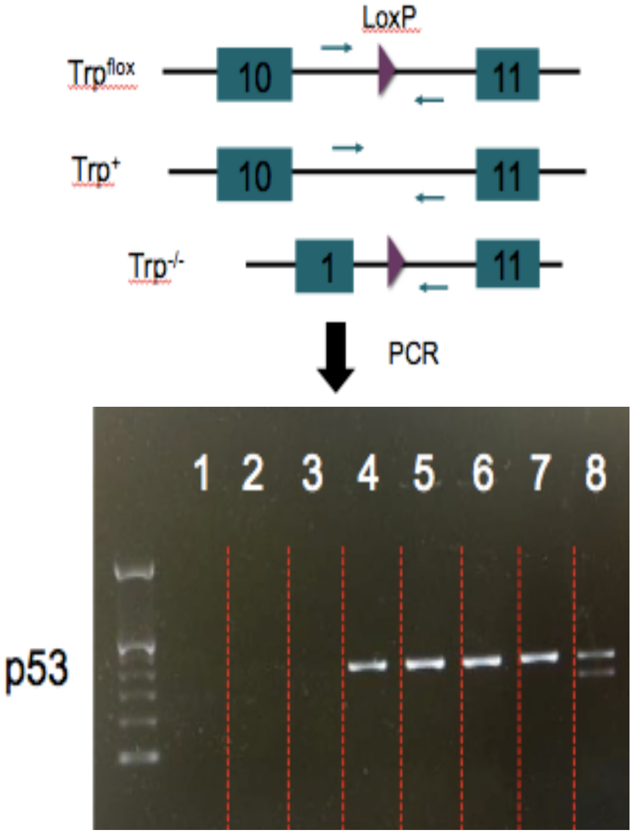

Organoids (after cre)

1. Trp53$^{-/-}$

2. Trp53$^{-/-}$

3. Trp53$^{-/-}$

Mouse (before cre)

4. Trp53$^{flox/flox}$

5. Trp53$^{flox/flox}$

6. Trp53$^{flox/flox}$

7. Trp53$^{flox/flox}$

8. Trp53$^{+/flox}$

Figure S1. Cre-mediated deletion of floxed *Trp53* is confirmed by genotyping. The PCR scheme of genotyping is at the top and the PCR result is at the bottom. As expected, no products are generated from the *Trp53*$^{-/-}$ organoid samples (Well 1-3), one band is visible in the *Trp53*$^{flox/flox}$ mouse samples (Well 4-7), and two bands are visible from the *Trp53*$^{+/flox}$ mouse sample (Well 8). The PCR primers are blue arrows, and LoxP sites are purple arrowheads between exons. The PCR products run on 2% agarose gel.

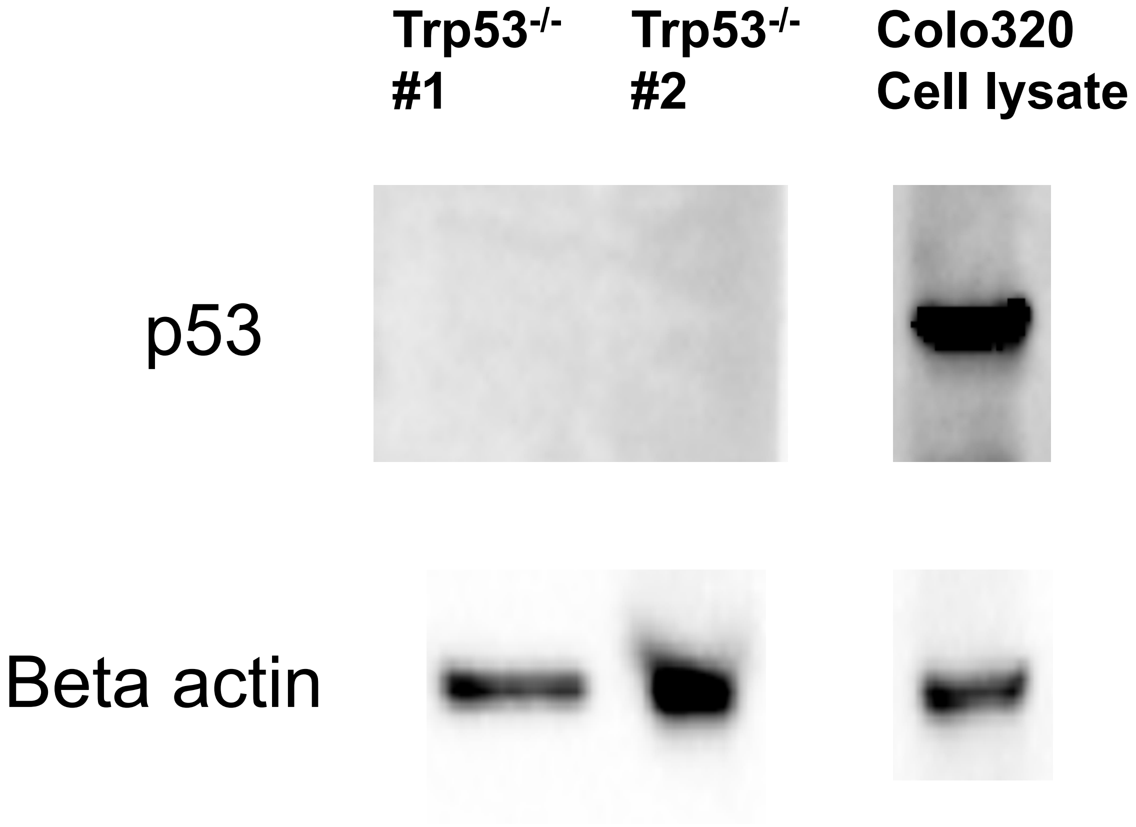

Figure S2. Loss of p53 is confirmed by western blotting. No p53 protein is detected in the *Trp53*$^{-/-}$ organoids cultured for two months (*Trp53*$^{-/-}$ #1) and three months (*Trp53*$^{-/-}$ #2). The whole cell lysate from Colo320 is used as the positive control for p53 protein. Beta actin is used as the loading control.

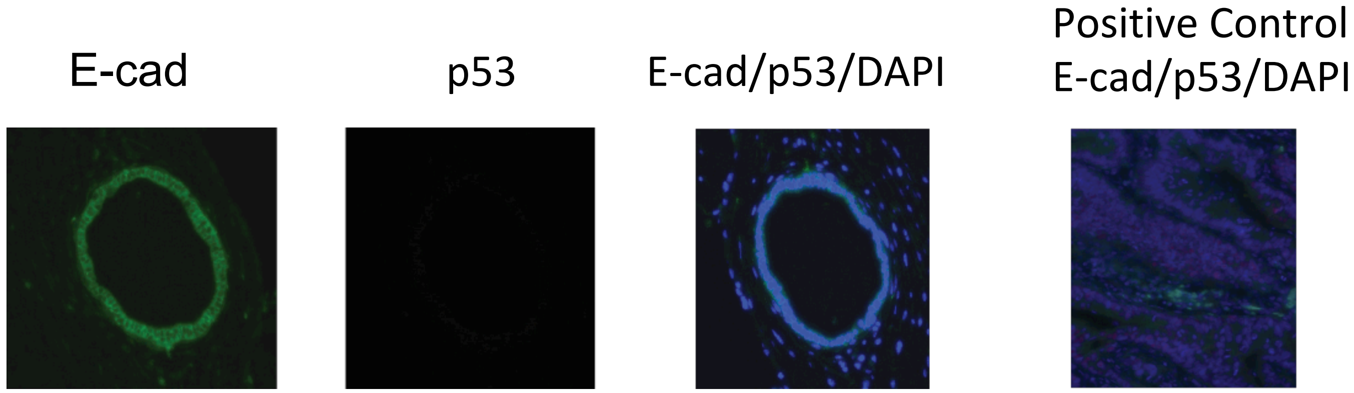

Figure S3. Loss of p53 expression in the *Trp53*-/- organoids using immunofluorescence (IF). E-cadherin (E-cad) is expressed in epithelial cells (green signal), and no expression of p53 was detected (red signal). Nuclei are counterstained with DAPI (blue signal). A colon tumor tissue section with p53 expression is used as a positive control.

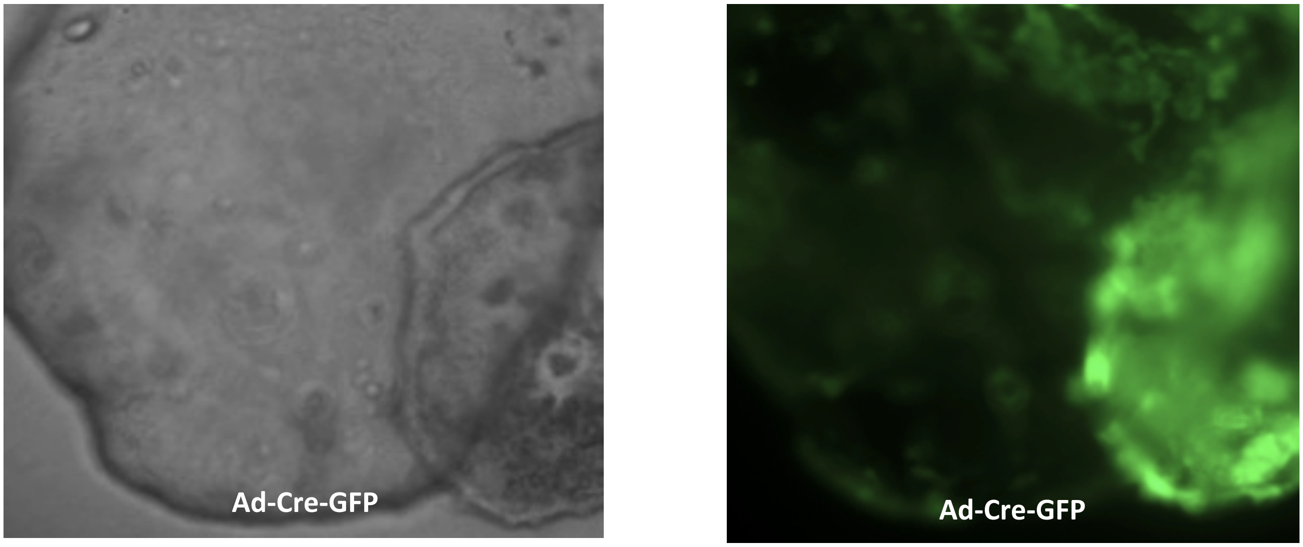

Figure S4. Gastric tissue explants infected with Ad-Cre-GFP. The cystic structures of tissue explants in collagen are examined under bright light (left panel) and fluorescent light (right panel) at day 3 post Ad-Cre-GFP infection.

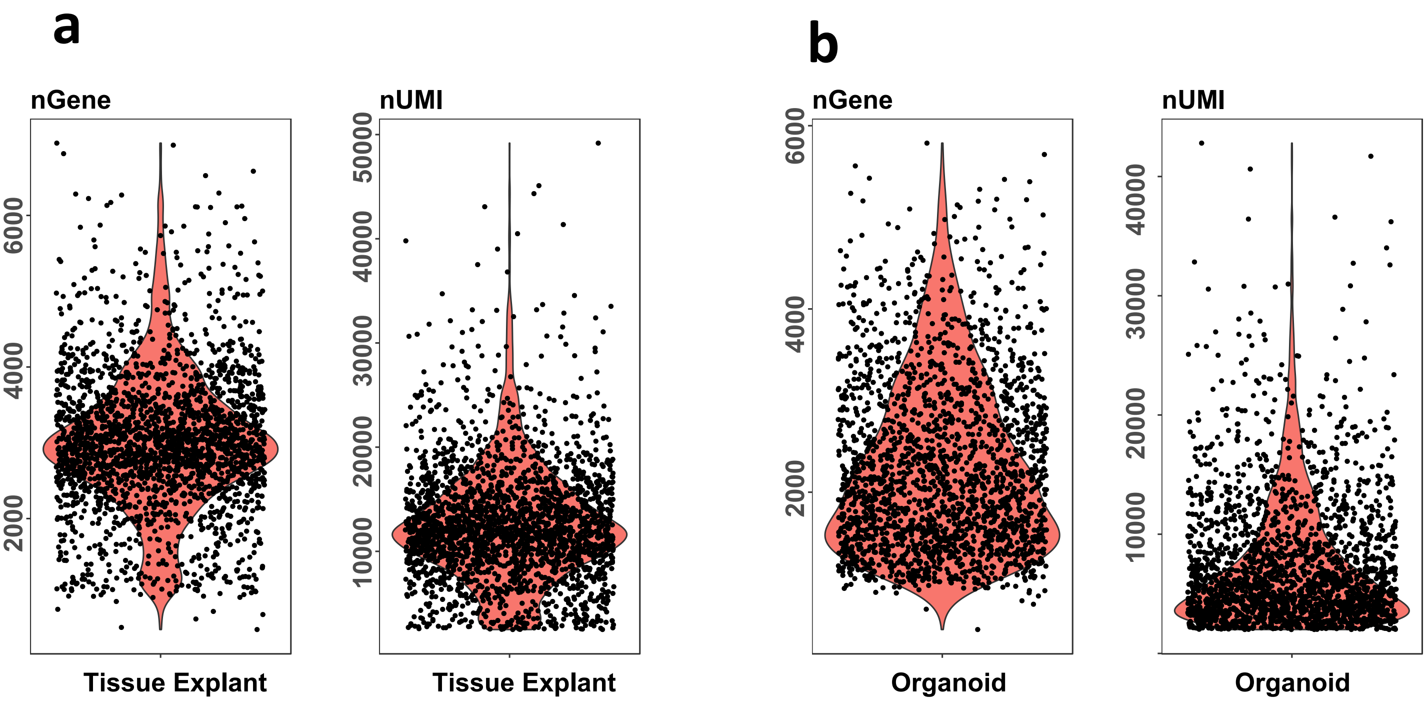

Figure S5. The distribution of number of genes and UMI counts in tissue explants and organoids. Violin plots display the distribution of number of genes (nGene) and number of unique mRNA transcript (nUMI) in single cells from tissue explant (a), Organoid (b). Each dot represents a single cell.

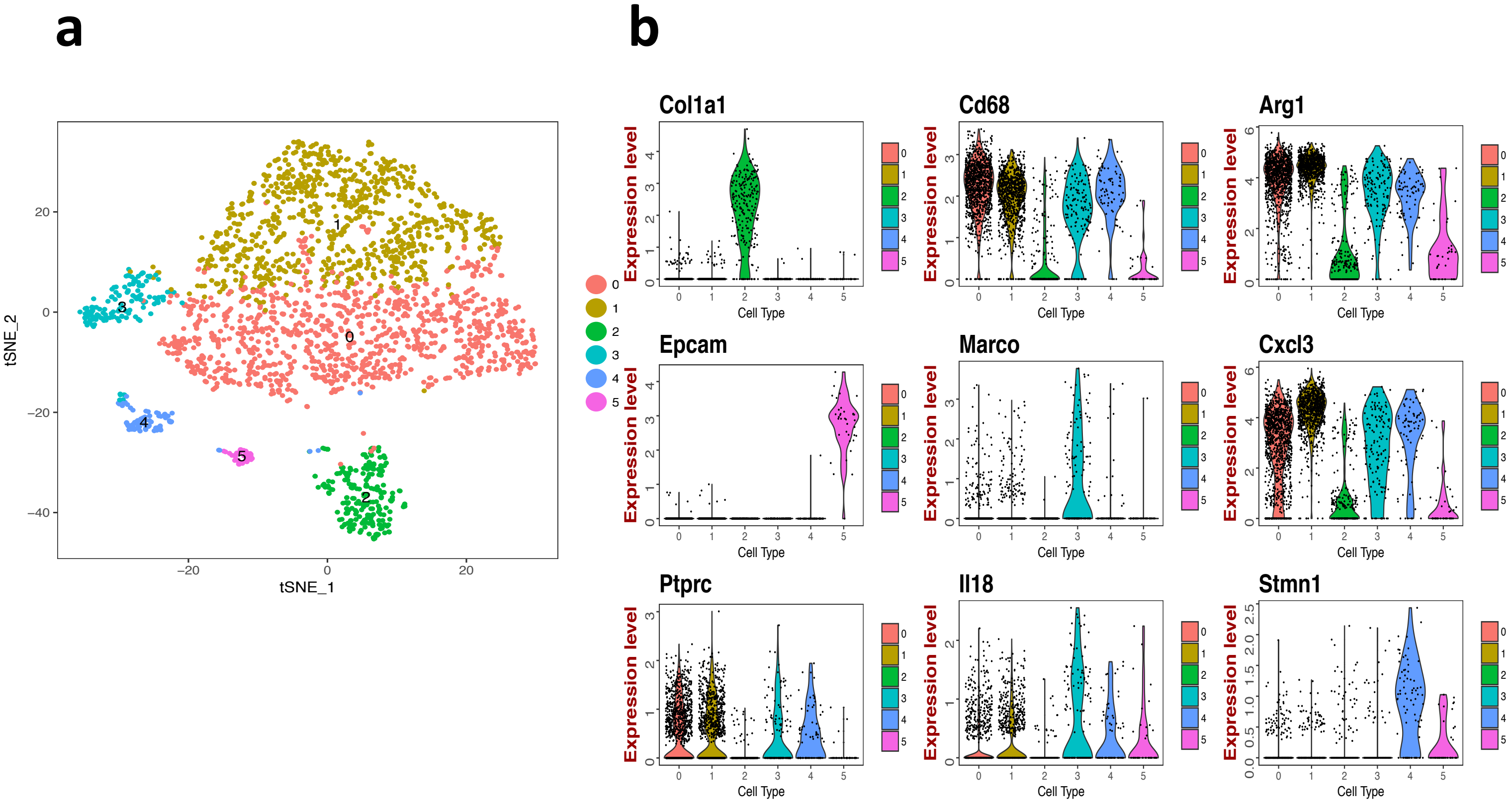

Figure S6. An independent scRNA library confirmed cell types in ALI cultured tissue explants. (a) A tSNE projection of 1961 cells from tissue explants. Cells are grouped into six clusters (0, 1, 2, 3, 4, 5) based on transcriptome profiles and are colored accordingly. The cell type assignment of each cluster is based on the gene expression analysis. (b) The expression of cell type specific genes in each cluster are displayed on violin plots. Specifically, fibroblast specific gene *Col1a1* in Cluster 2, epithelial specific gene *Epcam* in Cluster 5, and macrophage marker *Cd68* in Cluster 0, 1, 3, 4. Again, we observed three distinct subtypes of macrophage: Cluster 0 & 1 − Arg1 (M2 like macrophage), Cluster 3 − Macro & Il18 (M1 like macrophage), and Cluster 4 − Stmn1 (proliferating macrophage). Every dot represents an individual cell in violin plots, and the gene expression level is the natural log of the normalized UMI counts.

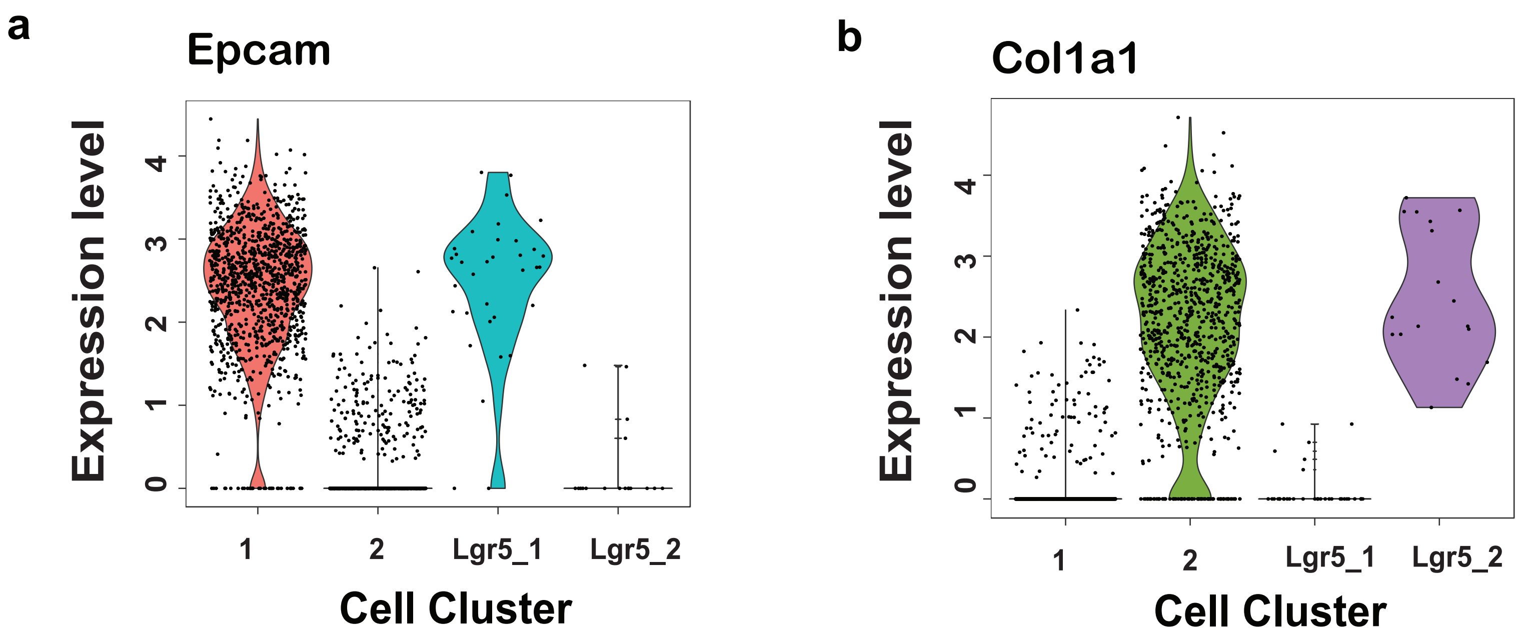

Figure S7. The expression of epithelial and fibroblast lineage markers, *Col1a1* (a) and *Epcam* (b), in Lgr5+ cells. Cluster 1 – Epithelial, Cluster 2– Fibroblast. Lgr5_1 is Lgr5+ cells in epithelial cell cluster and Lgr5_2 is Lgr5+ cells in fibroblast cell cluster. The expression level of violin plot is the natural log of the normalized UMI counts.

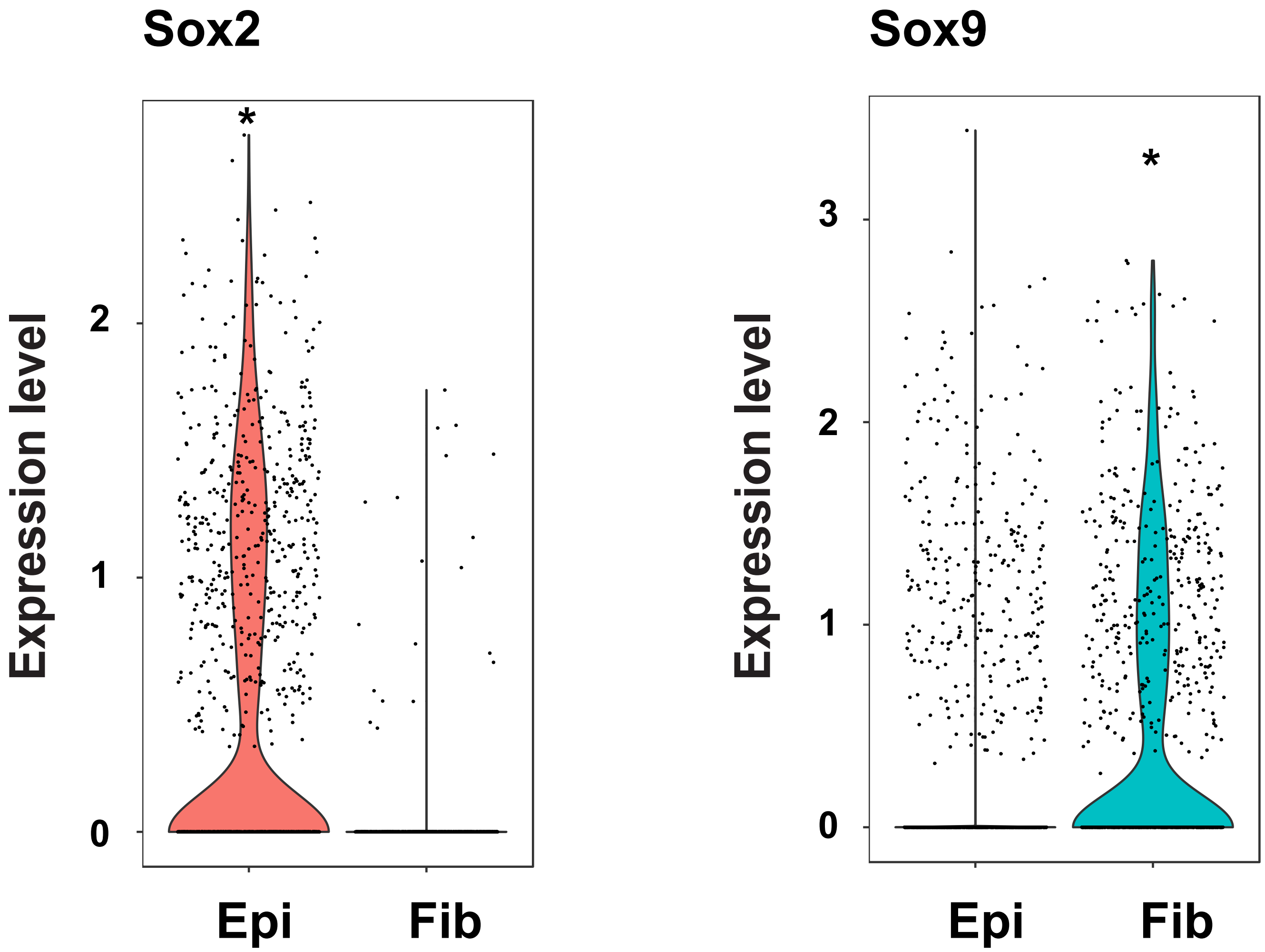

Figure S8. The expression of lineage specific transcriptional factors, Sox2 and Sox9 in epithelial and fibroblast cell types. The expression level on Y-axis is the natural log of the normalized UMI counts. * Bonferroni adjusted p <0.001. Epi - Epithelial, Fib – Fibroblast.

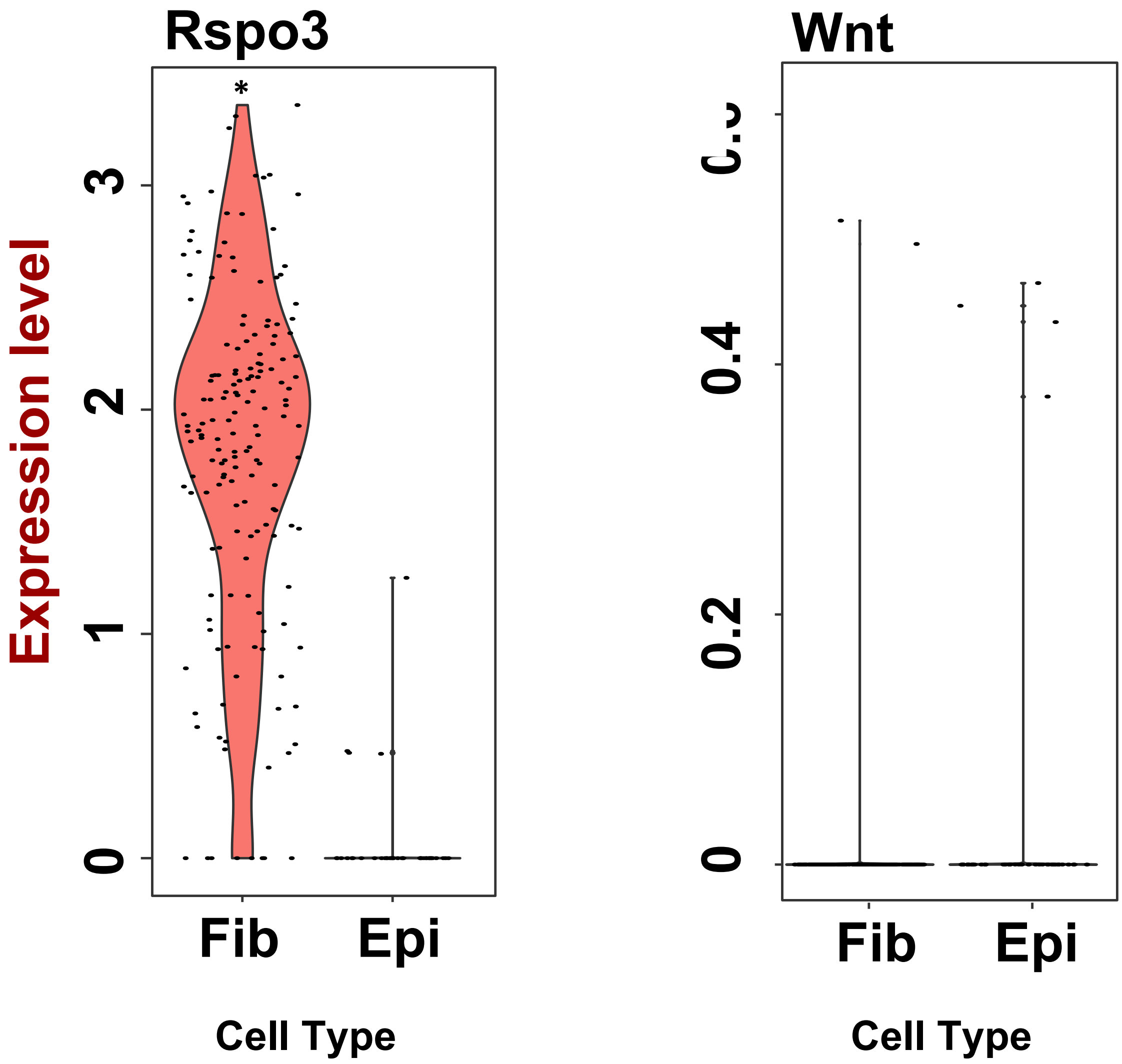

Figure S9. Rspo3 and Wnt4 expression in tissue explants . The expression level on Y-axis is the natural log of the normalized UMI counts. Epi – Epithelial, Fib – Fibroblast. * Bonferroni adjusted p <0.001

Ppib – green Polr2a - red **E. coli DapB - green**

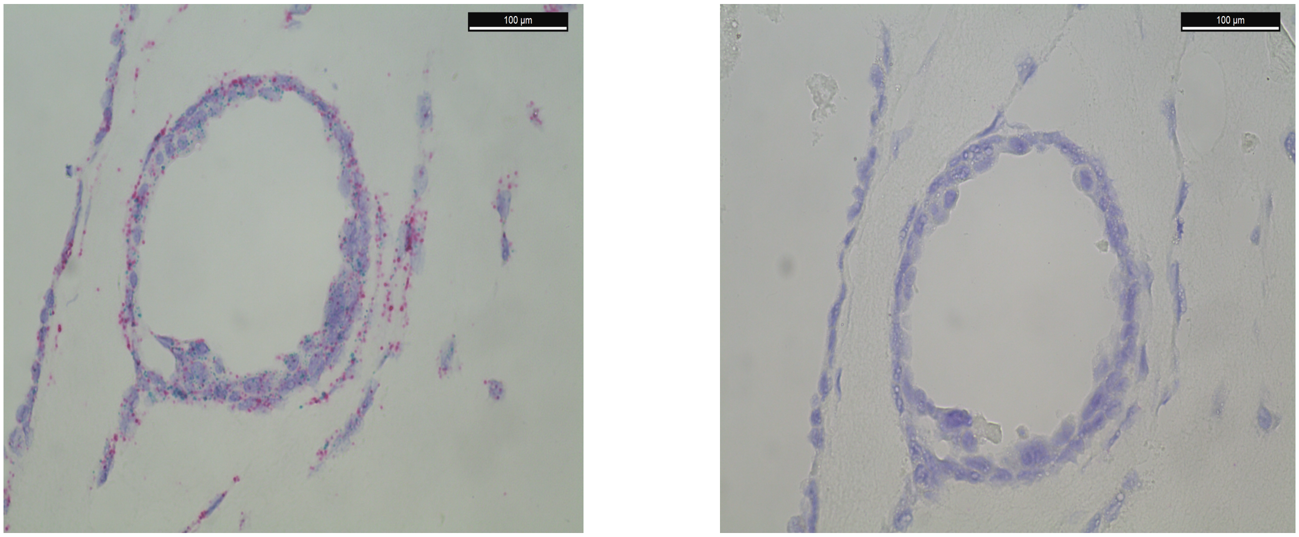

Figure S10. Evaluation of RNA ISH by targeting housekeeping genes and an E.coli gene. Two mouse housekeeping genes Ppib (green) and Polr2a (red) are detected simultaneously in all organoid cells. Negative control E.coli dapB mRNA is undetectable.

Table S1: Top 50 genes enriched in each cluster identified by differential analysis in tissue explants.

Cluster	gene	p value	fold change *
1	Sparc	0	3.55
1	Dcn	0	3.45
1	Bgn	0	3.28
1	Col1a1	1.618916818	3.04
1	Serpinh1	1.07E-307	2.90
1	Col3a1	1.82E-303	3.06
1	Grem1	1.81E-285	2.70
1	Igfbp7	3.12E-285	3.02
1	Col1a2	9.07E-284	2.65
1	Timp1	1.78E-281	3.03
1	Rcn3	5.17E-281	2.42
1	Mgp	1.03E-272	3.12
1	Efemp2	9.63E-272	1.57
1	Igf1	7.76E-265	2.63
1	Loxl1	3.43E-243	1.87
1	Mfap4	6.61E-243	2.59
1	Serping1	2.28E-240	2.43
1	Tmem176b	1.38E-239	2.55
1	Serpina3n	4.93E-239	2.21
1	Tmem176a	2.40E-235	2.42
1	Pcolce	5.50E-234	2.23
1	Wbp5	1.17E-231	1.55
1	Selm	7.49E-227	1.91
1	Map1lc3a	5.46E-225	1.34
1	Rspo3	1.08E-222	2.04
1	Sfrp1	1.12E-220	2.32
1	Serpinf1	3.07E-216	2.20
1	Prnp	7.38E-216	1.66
1	Col5a2	1.45E-213	1.78
1	Olfml3	2.41E-209	1.67
1	Itm2a	1.17E-208	2.15
1	Mylk	2.00E-207	1.85
1	Nedd4	4.28E-206	1.73
1	Mfap2	7.69E-205	1.73
1	Oat	3.38E-204	1.18
1	Mxra8	5.82E-204	1.39
1	Fbln2	1.92E-202	2.08
1	Serpine2	3.61E-202	2.42
1	Lox	6.65E-194	1.59
1	Col4a2	7.25E-191	1.45
1	Sod3	6.77E-189	1.80

Table S1: Top 50 genes enriched in each cluster identified by differential analysis in tissue explants.

Cluster	gene	p value	fold change *
1	Myl9	1.75E-188	2.27
1	Plod2	8.07E-185	1.56
1	Wisp1	4.32E-184	1.34
1	Aebp1	5.57E-184	1.57
1	Gpx8	1.56E-183	1.50
1	Meg3	8.65E-179	2.44
1	Cxcl12	5.49E-178	2.12
1	C1s1	8.31E-178	1.28
1	Tpm1	9.19E-178	1.61
2	Clu	5.93E-195	3.76
2	Gsto1	1.05E-139	2.15
2	Krt14	4.50E-126	3.37
2	Wfdc2	9.97E-123	3.18
2	Spint2	1.53E-119	2.66
2	Sfn	1.62E-117	2.85
2	Krt7	5.32E-112	3.16
2	Cldn4	1.85E-107	2.86
2	Ly6a	5.14E-107	3.18
2	Nupr1	3.39E-97	2.49
2	Ltf	1.20E-96	2.73
2	Epcam	5.21E-91	2.67
2	Krt18	4.79E-88	2.73
2	Perp	1.27E-86	2.13
2	Gsta4	5.65E-85	2.33
2	Krt19	9.27E-82	2.81
2	Fxyd3	2.24E-81	2.15
2	Krt8	8.70E-80	2.60
2	S100a14	4.71E-78	2.28
2	Cldn7	1.13E-76	1.92
2	Tmc4	1.08E-73	1.30
2	Gpx2	1.83E-72	1.75
2	Krt17	4.85E-69	2.77
2	F11r	7.77E-65	1.23
2	2200002D01	4.65E-62	1.78
2	Krtcap3	4.76E-62	1.05
2	Cystm1	7.38E-62	1.39
2	Urah	5.14E-61	1.19
2	Anxa8	3.75E-60	1.78
2	S100a16	8.13E-60	1.99
2	Psca	1.15E-59	2.07
2	Pdlim1	2.50E-59	1.55

Table S1: Top 50 genes enriched in each cluster identified by differential analysis in tissue explants.

Cluster	gene	p value	fold change *
2	Serpinb5	3.11E-59	0.93
2	Dsp	9.46E-58	0.94
2	Tnfrsf12a	2.29E-57	1.71
2	Lamc2	4.85E-57	1.47
2	Tacstd2	7.79E-57	1.70
2	Spint1	1.36E-56	1.12
2	Rab25	1.46E-55	1.08
2	Lad1	1.74E-55	1.05
2	Tpm1	4.05E-55	1.77
2	Ly6d	8.98E-55	2.30
2	Serinc2	1.34E-54	1.24
2	Fermt1	1.77E-54	1.11
2	Crb3	4.81E-54	1.10
2	Jup	6.93E-54	1.01
2	Klf5	7.51E-54	1.26
2	Sox11	1.60E-52	0.87
2	Sdc1	1.69E-52	1.78
2	Smim22	4.00E-52	0.90
3	Ear2	8.57E-116	1.73
3	Ltc4s	6.54E-96	1.32
3	Mpc2	1.52E-89	0.98
3	Wfdc21	1.72E-89	2.14
3	Il18	4.95E-66	0.71
3	Abcg1	4.95E-66	0.75
3	Ramp1	6.75E-66	1.03
3	Etfb	1.34E-56	0.70
3	Unc119	1.39E-56	0.63
3	Sept9	2.51E-51	0.56
3	Axl	3.03E-49	0.77
3	Chil3	2.36E-48	1.50
3	Ucp2	5.33E-48	0.84
3	Klhdc4	3.93E-46	0.71
3	Marco	7.68E-46	1.67
3	Gngt2	3.62E-38	0.74
3	Abcc5	7.85E-38	0.51
3	Ear1	6.88E-37	0.52
3	Sort1	6.93E-37	0.52
3	Pmvk	2.53E-36	0.48
3	Serpinb1a	9.63E-36	0.79
3	Gm1673	9.83E-36	0.79
3	Msrb1	1.47E-35	0.66

Table S1: Top 50 genes enriched in each cluster identified by differential analysis in tissue explants.

Cluster	gene	p value	fold change *
3	Dgat1	2.23E-32	0.54
3	Ly75	1.33E-31	0.41
3	Ndufc1	1.48E-31	0.52
3	Adipor2	2.00E-31	0.46
3	Syk	3.57E-31	0.48
3	Prkcd	1.86E-30	0.54
3	Fcor	4.19E-30	0.55
3	N4bp1	4.45E-30	0.41
3	S100a1	1.16E-29	0.75
3	Siglece	2.02E-29	0.43
3	2700094K13I	2.97E-29	0.57
3	Acp5	3.45E-29	1.03
3	Iqsec1	3.58E-29	0.44
3	Mgll	4.69E-29	0.76
3	Hebp1	5.07E-29	0.56
3	Ccnd2	4.14E-27	0.67
3	Sirpa	2.51E-26	0.56
3	Cd200r2	2.25E-25	0.44
3	Olr1	1.40E-23	0.50
3	F10	2.85E-22	0.74
3	Serpine1	7.95E-22	0.83
3	Car4	2.39E-20	1.02
3	Tgm2	7.85E-18	0.49
3	Lpl	8.26E-18	0.51
3	Plet1	1.04E-16	0.82
3	Atp6v0d2	1.50E-16	0.58
3	Csf2rb	4.79E-13	0.44
4	Fxyd5	0	0.85
4	Lgals3	0	0.77
4	Tyrobp	0	0.73
4	Fcer1g	0	0.73
4	Ctss	1.136350985	0.93
4	Lilrb4a	1.854228368	1.13
4	Srgn	3.56E-280	1.24
4	H2-D1	7.74E-271	0.96
4	Arg1	7.25E-262	1.17
4	Capg	1.11E-256	0.63
4	Fcgr3	1.25E-256	1.22
4	Fcgr2b	1.49E-218	1.36
4	Trf	4.00E-213	1.08
4	Clec4d	3.93E-212	1.04

Table S1: Top 50 genes enriched in each cluster identified by differential analysis in tissue explants.

Cluster	gene	p value	fold change *
4	Ctsc	1.48E-207	0.93
4	Cd53	9.60E-204	1.25
4	Ccl3	2.33E-189	0.97
4	Ctsa	7.79E-184	0.74
4	Mmp12	4.33E-183	1.66
4	Cd68	5.67E-183	0.91
4	Lilr4b	1.48E-169	0.91
4	Pf4	7.23E-166	1.46
4	Lcp1	3.27E-163	0.75
4	Pla2g7	7.88E-158	1.25
4	Adam8	6.04E-155	1.10
4	Mmp9	7.95E-154	1.41
4	Grn	4.32E-151	0.60
4	Hilpda	5.50E-147	0.86
4	Bcl2a1b	3.38E-137	0.80
4	Sirpb1c	3.55E-134	0.94
4	Rac2	2.98E-133	0.67
4	Ms4a6d	7.13E-131	0.97
4	Ncf4	2.79E-129	0.79
4	Card19	2.69E-128	0.83
4	Itgam	4.27E-128	0.87
4	Rgs1	1.22E-127	1.30
4	Cd52	6.06E-126	0.73
4	Emb	1.20E-125	0.93
4	Mpeg1	9.86E-124	0.75
4	Cfp	3.07E-123	0.92
4	Ccl9	1.18E-120	0.75
4	Trem2	1.24E-120	0.84
4	Cxcl3	1.28E-117	1.22
4	Msr1	7.50E-117	0.77
4	Ccr1	1.42E-116	1.00
4	Adgre1	1.39E-108	0.82
4	Hmox1	6.06E-108	0.82
4	Pid1	1.35E-107	1.01
4	Mmp8	1.79E-107	1.44
4	Itgb2	3.50E-107	0.71
5	Tuba1b	2.15E-105	1.70
5	Cdk1	1.53E-99	1.41
5	Stmn1	5.02E-94	1.67
5	Birc5	1.21E-81	1.59
5	Top2a	7.80E-80	1.38

Table S1: Top 50 genes enriched in each cluster identified by differential analysis in tissue explants.

Cluster	gene	p value	fold change *
5	Cdca3	1.50E-77	1.35
5	Tubb5	1.95E-75	1.46
5	2810417H13I	1.01E-71	1.94
5	Ube2c	6.37E-71	1.85
5	Pmf1	6.03E-69	1.06
5	Spc24	1.90E-67	1.13
5	Cks1b	1.25E-66	1.28
5	H2afv	2.00E-64	1.19
5	Pttg1	1.18E-60	1.26
5	Tk1	7.04E-59	1.08
5	H2afz	2.87E-58	1.03
5	Ccna2	4.17E-58	0.76
5	Smc2	2.95E-57	1.12
5	Cdca8	7.61E-57	1.03
5	Ccnb1	3.56E-56	0.74
5	Pbk	4.53E-56	0.75
5	Mki67	9.38E-55	1.22
5	Racgap1	3.57E-54	0.73
5	Prc1	7.22E-53	0.67
5	Hmgb2	1.60E-52	1.07
5	Dut	3.65E-50	1.01
5	Asf1b	1.02E-49	0.82
5	Tyms	4.88E-49	1.05
5	Rrm2	1.02E-48	0.89
5	Cenpa	1.68E-48	1.14
5	Smc4	2.47E-48	0.99
5	Ccnb2	3.17E-48	0.93
5	Spc25	3.33E-48	0.84
5	Nusap1	5.33E-48	0.64
5	Cenpf	1.54E-46	0.72
5	Plk1	1.05E-44	0.45
5	Hmmr	1.15E-44	0.56
5	2700094K13I	2.28E-43	1.04
5	Gmnn	8.46E-43	0.91
5	Cenpm	3.04E-42	0.59
5	Aurkb	3.45E-42	0.57
5	Tacc3	1.55E-41	0.75
5	Cenpe	2.80E-41	0.70
5	Pcna	1.05E-39	0.86
5	Hist1h1b	1.88E-39	0.54
5	Tpx2	3.07E-39	0.69

Table S1: Top 50 genes enriched in each cluster identified by differential analysis in tissue explants.

Cluster	gene	p value	fold change *
5	Rad51	1.02E-38	0.51
5	Ranbp1	1.92E-38	0.90
5	Ube2t	1.94E-38	0.57
5	Rrm1	2.75E-36	0.73

* fold change for differentially expressed genes in each cluster is natural log transformed.

Table S2:Cluster specific genes identified by the differential analysis in the Organoid.

cluster	gene	p value	fold change *
1	Sparc	0	2.823106377
1	Col3a1	0	2.342912674
1	Lgals1	0	2.337714967
1	Bgn	0	2.310103026
1	Igfbp7	0	2.276159903
1	Col1a1	0	2.226378528
1	Vim	0	2.095929529
1	Serpinh1	0	2.079029089
1	Serpinf1	0	1.966261368
1	Rcn3	0	1.894706649
1	Col1a2	0	1.882037613
1	Aebp1	0	1.864543768
1	B2m	0	1.856069855
1	Selm	0	1.721362686
1	Cst3	0	1.534867618
1	Cald1	1.22972939	1.604614759
1	Id3	2.76E-289	2.135119774
1	Col5a2	1.55E-288	1.647767803
1	Mxra8	1.07E-287	1.561020863
1	Timp1	3.51E-283	2.023371424
1	Pcolce	1.16E-266	1.592761513
1	Cpxm1	2.16E-260	1.708798992
1	Cd302	5.66E-256	1.252557056
1	Mfap2	1.43E-254	1.324882514
1	Fbln2	6.55E-247	1.523486824
1	Dcn	1.58E-245	2.240119026
1	Wbp5	1.49E-241	1.374587992
1	Timp2	5.43E-237	1.235175164
1	Tceal8	7.41E-234	1.326985066
1	Efemp2	1.54E-232	1.314160723
1	Ctsz	4.90E-231	1.19237118
1	Ctla2a	2.24E-228	2.452867473
1	Nsg1	2.95E-227	1.345003951
1	Loxl1	7.51E-223	1.157656427
1	Eva1b	1.59E-220	1.204036317
1	Cxcl12	3.32E-220	1.79140212
1	Igfbp4	1.44E-217	1.563361818
1	Tmsb10	2.00E-216	1.17113605
1	Sfrp1	1.65E-211	2.068755725
1	Cdkn2a	1.16E-208	1.178921582
1	Dynlrb1	1.13E-206	0.918181808

Table S2:Cluster specific genes identified by the differential analysis in the Organoid.

cluster	gene	p value	fold change *
1	Tagln	1.48E-206	1.773083318
1	Myl9	3.56E-205	1.735182426
1	Slc25a4	8.30E-203	0.781003512
1	Cmtm3	3.44E-189	1.036968519
1	Fstl1	1.34E-188	1.134151368
1	Ccdc80	3.22E-188	1.487701293
1	Emp3	3.87E-185	1.160380429
1	Klf2	2.92E-184	1.720982709
1	Tuba1a	9.83E-182	1.10258012
1	Nradd	5.19E-177	0.829975834
1	Mfap5	2.43E-173	1.570080775
1	Fkbp7	6.41E-173	0.920517298
1	Olfml3	8.50E-169	1.034695728
1	Gpx7	3.07E-168	0.871665446
1	Copz2	1.72E-165	0.950478243
1	Grem1	1.72E-164	1.243865757
1	Serping1	1.75E-164	1.453911548
1	Lrp1	2.26E-164	1.058258171
1	Gng11	3.51E-162	1.040215385
1	Tubb6	3.83E-162	0.922207065
1	Prrx1	3.71E-161	0.912486978
1	Tpm1	1.37E-158	1.064227929
1	Wisp1	5.58E-158	0.942127292
1	Plat	1.21E-157	0.992409064
1	Rcn1	2.26E-157	0.92348993
1	Mfap4	1.22E-156	1.434147028
1	Rhoc	2.56E-156	0.944064681
1	Itm2a	4.50E-156	1.364623765
1	Maged2	1.13E-155	0.937703177
1	Pdlim2	4.13E-155	1.091061787
1	Ctsl	7.94E-155	0.85476355
1	Ctsk	2.27E-154	0.950439464
1	Cryab	1.37E-153	1.195907564
1	Tspan4	2.70E-151	0.991741107
1	Rbp1	1.34E-149	1.557940113
1	Csf1	1.62E-149	1.024696752
1	Fbln1	3.87E-148	1.113955084
1	Lhfp	6.87E-148	0.882464363
1	Nnmt	7.65E-148	0.792178731
1	Col12a1	9.96E-147	1.057037914
1	Fcgrt	5.56E-146	0.878838571

Table S2:Cluster specific genes identified by the differential analysis in the Organoid.

cluster	gene	p value	fold change *
1	Rrbp1	5.63E-145	0.898576516
1	Lox	4.21E-144	0.939329828
1	Fhl2	4.05E-142	1.092939978
1	Colec12	6.75E-142	0.715635669
1	Plac8	1.37E-135	1.380802556
1	Smim3	6.18E-134	0.879690061
1	Ccl2	2.14E-133	2.095040473
1	Tpm2	5.98E-133	1.0556755
1	Col6a1	7.88E-133	0.75087847
1	Ctsb	7.34E-132	0.778124065
1	Pdgfrb	7.70E-131	0.706540967
1	Calu	1.49E-130	0.872245784
1	Prkcdbp	1.72E-130	0.827357452
1	Col5a1	4.08E-130	0.780999344
1	Bst2	5.07E-130	1.070186434
1	Acta2	1.54E-129	1.979004936
1	Fkbp10	1.57E-129	0.683817728
1	Ccl7	3.39E-129	1.818360978
1	S100a1	1.48E-128	0.816236623
1	Fkbp1a	3.28E-128	0.73114375
1	Nbl1	4.25E-128	1.039864437
1	Postn	2.00E-127	1.162605445
1	H13	2.65E-127	0.830680073
1	Rasl11a	1.04E-126	1.354354938
1	Scarf2	1.07E-126	0.761975342
1	Il11ra1	2.10E-126	0.797532994
1	Timp3	4.24E-126	1.395347961
1	Mxra7	7.17E-126	0.697404852
1	Sdc2	1.02E-125	1.012867782
1	Map1lc3a	1.27E-125	0.765807669
1	Lbh	6.83E-125	0.997821591
1	Hand2	7.45E-125	0.803650494
1	Figf	2.46E-124	1.479581895
1	Cdh11	3.32E-124	0.703610037
1	Itm2c	9.18E-124	0.807540855
1	Snai1	4.93E-123	0.779286191
1	Lpl	6.37E-123	1.286473819
1	Ppib	1.44E-120	0.666415348
1	Fgfr1	2.51E-120	0.742234439
1	Kdelr3	4.37E-118	0.735034963
1	Hsd11b1	8.46E-118	1.240204292

Table S2:Cluster specific genes identified by the differential analysis in the Organoid.

cluster	gene	p value	fold change *
1	Lix1l	4.38E-117	0.573224634
1	Cnn2	1.98E-116	0.923464681
1	Thy1	2.15E-115	1.272121078
1	Pdpn	2.94E-114	0.840879948
1	Hmox1	6.52E-114	1.194214954
1	Igf1	8.94E-113	0.847002882
1	Rspo3	1.15E-112	0.838615215
1	Emilin1	1.17E-112	0.586386903
1	Plod2	1.62E-112	0.741306546
1	Ndn	8.51E-112	0.711366458
1	Cebpb	1.07E-111	0.884446631
1	Meg3	5.28E-111	1.997932511
1	Ier3	7.36E-111	1.113974847
1	Saa3	1.97E-110	2.902800309
1	Tfpi	6.21E-110	0.564212392
1	Tceb2	4.34E-108	0.550699874
1	Tspan3	7.04E-108	0.744610822
1	Gpx8	1.48E-106	0.753752613
1	Serpine1	4.23E-106	1.034815079
1	C1s1	6.53E-106	0.706323163
1	Fkbp11	1.66E-105	0.733025609
1	Bicc1	3.17E-105	0.626308162
1	Islr	3.55E-105	0.584305163
1	Tmed3	5.98E-104	0.727785105
1	Maged1	1.04E-103	0.77721257
1	Hexa	5.80E-103	0.816447334
1	7-Sep	4.52E-102	0.778605551
1	Nr2f1	4.73E-102	0.641469151
1	Ptgis	1.06E-100	0.628954955
1	Fbn1	1.59E-100	0.662401291
1	Snhg18	1.10E-99	0.729255963
1	Dpysl3	1.46E-99	0.748317274
1	Calr	3.50E-99	0.667997793
1	Fxyd6	6.20E-99	1.065114166
1	Gas1	1.08E-98	0.765901494
1	Vcam1	7.37E-98	0.759192812
1	Qpct	7.73E-98	0.652961692
1	Fermt2	2.04E-97	0.660854086
1	Tmem50a	2.95E-97	0.656956334
1	Rcn2	6.69E-97	0.690610984
1	Sec61b	4.66E-96	0.620948071

Table S2:Cluster specific genes identified by the differential analysis in the Organoid.

cluster	gene	p value	fold change *
1	Txndc5	8.10E-96	0.722729819
1	Arxes2	9.10E-96	0.714804882
1	Tgfbi	1.05E-95	0.850344407
1	Ppic	1.32E-94	0.756223774
1	Pde1a	1.87E-94	0.626470769
1	Flnc	3.44E-94	0.548087728
1	Tmem119	6.27E-94	0.533034014
1	Creb3l1	9.00E-94	0.477156553
1	C1ra	1.95E-92	0.681042366
1	Thbs1	2.06E-92	0.912104528
1	Ngfrap1	2.68E-92	0.770905438
1	Smim1	3.91E-92	0.583984718
1	Ifi27	4.98E-92	0.709394371
1	Flot1	5.35E-92	0.710079946
1	Sdpr	1.15E-91	0.623141464
1	Prdx4	4.66E-91	0.73096041
1	Tnc	7.76E-91	0.951001424
1	Thbd	1.82E-90	0.73723708
1	Mdk	1.15E-89	0.961475631
1	Tpm4	2.90E-89	0.755938973
1	Tm4sf1	7.82E-88	0.646531627
1	Nid1	8.49E-88	0.496880531
1	Lgi2	2.59E-87	0.6823409
1	Mmp23	3.46E-87	0.593120946
1	Tmem45a	3.54E-87	0.490062565
1	Arpc1b	5.68E-87	0.563994476
1	Glipr2	8.49E-87	0.521599186
1	Ak1	1.15E-86	0.493778808
1	Hsp90b1	1.82E-86	0.670993569
1	Has2	4.06E-86	0.635016133
1	Adamts2	7.16E-86	0.554120009
1	Eif4ebp1	9.33E-86	0.740707242
1	Il6st	1.22E-85	0.739351978
1	Fgf7	1.24E-85	0.608154375
1	Renbp	3.08E-85	0.489196079
1	Spp1	6.19E-85	1.681318203
1	Loxl2	8.48E-85	0.500665477
1	Osmr	1.50E-84	0.514196826
1	Gdpd2	1.58E-84	0.72820607
1	Pmp22	1.32E-83	0.682424745
1	Pdgfra	3.58E-83	0.534292242

Table S2:Cluster specific genes identified by the differential analysis in the Organoid.

cluster	gene	p value	fold change *
1	Mmp19	4.22E-83	0.570409406
1	Oaf	3.48E-82	0.56488903
1	Lsp1	3.75E-82	0.515514781
1	Apbb1ip	4.52E-81	0.449880286
1	Tenm3	1.69E-80	0.458136846
1	Mmp3	3.09E-80	1.829315927
1	Nrp1	7.84E-80	0.568426528
1	Col4a1	8.09E-80	0.568645188
1	Gadd45b	1.04E-79	0.756358788
1	Msn	9.50E-79	0.576553748
1	Arxes1	1.13E-78	0.541659332
1	Jam3	4.05E-78	0.463255798
1	Rgs16	4.10E-77	0.619097933
1	Mageh1	4.64E-77	0.446099501
1	Tshz2	9.78E-77	0.822701755
1	Plxdc2	3.45E-76	0.557799471
1	Thbs2	1.98E-75	0.570922954
1	Gem	4.84E-75	0.619670643
1	Itgb1	1.03E-74	0.641521882
1	Itga5	1.18E-74	0.521488485
1	Npc2	1.19E-74	0.59094458
1	Cpq	1.84E-74	0.413754296
1	Lmcd1	2.16E-74	0.798741865
1	P3h1	2.31E-74	0.401503028
1	Plpp3	2.66E-74	0.578771718
1	Nrn1	2.87E-74	0.577138252
1	Lamp1	1.18E-73	0.539709729
1	Grn	2.10E-73	0.6525983
1	Ddr2	4.11E-73	0.440810252
1	Gaa	6.93E-73	0.640001374
1	Prdx5	7.11E-73	0.604093205
1	Gadd45g	1.86E-72	0.870634748
1	Bdnf	2.00E-72	0.565930053
1	Steap1	2.08E-72	0.627933401
1	Gsn	2.43E-72	0.631978245
1	Aspn	2.79E-72	0.574535082
1	Clmp	1.70E-71	0.453543011
1	Sod3	1.86E-71	0.559632981
1	Mmp2	4.61E-71	0.532742095
1	Ppp1r18	1.21E-70	0.424872215
1	P3h3	1.30E-70	0.443591914

Table S2:Cluster specific genes identified by the differential analysis in the Organoid.

cluster	gene	p value	fold change *
1	Anxa6	2.21E-70	0.593251954
1	Cfh	2.36E-70	0.455347268
1	Rab31	2.92E-70	0.441842838
1	Serpina3g	1.39E-69	0.538681193
1	H6pd	2.06E-69	0.467681967
1	Slc7a2	2.11E-69	0.578691321
1	Sdf4	2.48E-69	0.54992905
1	Slit3	3.14E-69	0.466003059
1	Pde10a	1.10E-68	0.472493824
1	Egr1	6.25E-68	0.64385119
1	Lamp2	8.77E-68	0.606260581
1	Phldb2	1.23E-67	0.482702784
1	Vmp1	1.47E-67	0.583534449
1	Barx1	4.96E-67	0.464162775
1	Srpx2	8.52E-67	0.514211709
1	Tcea3	9.29E-67	0.470899516
1	Cyba	1.95E-66	0.489606336
1	Ikbip	5.60E-66	0.460135904
1	2210013O21Rik	5.78E-66	0.553199899
1	Slc16a2	6.37E-66	0.428421821
1	Nell2	6.60E-66	0.546541126
1	Ndufa2	1.23E-65	0.469009706
1	Isl1	3.80E-65	0.4300693
1	Fkbp9	4.08E-65	0.530876475
1	Man2a1	5.93E-65	0.484314333
1	Ltbp2	7.64E-65	0.487518002
1	Axl	9.35E-65	0.507397159
1	Hoxa5	1.70E-64	0.448518706
1	Actg2	7.48E-64	0.887611195
1	Crtap	1.12E-63	0.454546384
1	Pdlim4	4.15E-63	0.462370909
1	Slc43a3	1.74E-62	0.445430387
1	Clta	1.78E-62	0.437694755
1	Mmp14	2.04E-62	0.615452132
1	Col4a2	2.55E-62	0.46554643
1	Capzb	2.63E-62	0.497527753
1	Myl6	7.72E-62	0.536573942
1	Pros1	9.05E-62	0.454377125
1	Ptx3	9.89E-62	0.881320301
1	Inhba	1.46E-61	0.799514004
1	Fn1	1.59E-61	0.662384359

Table S2:Cluster specific genes identified by the differential analysis in the Organoid.

cluster	gene	p value	fold change *
1	Flna	1.71E-61	0.619046561
1	Sgce	3.13E-61	0.487731343
1	Mgp	3.92E-61	1.200253147
1	Svbp	1.18E-60	0.523627184
1	Bex1	2.75E-60	0.851318316
1	Cox7a2	1.37E-59	0.435678093
1	Gpm6b	1.82E-59	0.624572235
1	Mtch1	1.85E-59	0.505272208
1	Bnip2	3.79E-59	0.499938171
1	Ufc1	4.06E-59	0.53048799
1	Slco3a1	5.48E-59	0.450373289
1	Serf2	5.56E-59	0.522102729
1	Isg15	1.76E-58	0.792556705
1	B4galt5	1.80E-58	0.441856758
1	Lpar1	2.42E-58	0.421880496
1	Pea15a	3.03E-58	0.480595364
1	Lman1	3.39E-58	0.557908794
1	Ugdh	4.14E-58	0.736988087
1	Vcan	5.28E-58	0.435288081
1	Lxn	6.01E-58	0.662429103
1	Angptl2	6.59E-58	0.421360993
1	Hacd1	2.13E-57	0.522863971
1	Tnfrsf1a	2.33E-57	0.490796003
1	Akr1b8	3.55E-57	0.475960504
1	Creg1	4.87E-57	0.529594363
1	Grina	1.17E-56	0.546614982
1	Rab34	1.43E-56	0.527521446
1	Nedd4	1.96E-56	0.475437638
1	Timm8b	3.14E-56	0.506060146
1	Ddah2	4.47E-56	0.532414476
1	Derl1	5.62E-56	0.529296125
1	Rrad	1.16E-55	0.772493983
1	Plpp1	1.73E-55	0.44778835
1	Atp6v0b	1.81E-55	0.454193847
1	Srp14	2.63E-55	0.444211689
1	Kdelr2	2.79E-55	0.510564801
1	Vasn	5.00E-55	0.432230075
1	Scpep1	6.48E-55	0.551470497
1	Dad1	1.15E-54	0.405106975
1	Snhg6	1.21E-54	0.4991987
1	Macrod2	1.65E-54	0.420434618

Table S2:Cluster specific genes identified by the differential analysis in the Organoid.

cluster	gene	p value	fold change *
1	Ccdc107	1.77E-54	0.511929793
1	Dap	3.04E-54	0.575938098
1	Igfbp3	9.87E-54	1.0110424
1	Surf4	1.76E-53	0.503653483
1	Mif	1.76E-52	0.594396114
1	Copb2	3.43E-52	0.49031481
1	Tnfaip6	4.74E-51	0.769636045
1	Ssr2	9.50E-51	0.470195259
1	Hdlbp	1.57E-50	0.487214476
1	Ctsa	7.85E-50	0.520184738
1	Srpr	1.38E-49	0.449298857
1	Tmem258	1.00E-48	0.463332197
1	Clic4	1.54E-48	0.43154613
1	Ier5	1.87E-48	0.573847888
1	Tmem59	2.04E-48	0.414979739
1	Socs3	2.37E-48	0.549771542
1	Ptgs1	2.61E-48	0.502539163
1	Tbca	3.12E-48	0.42939578
1	Creb3l2	4.12E-48	0.42373904
1	Ddost	4.17E-48	0.46689201
1	P4hb	4.68E-48	0.472796648
1	Igfbp6	4.71E-48	0.569311512
1	Ncam1	7.89E-48	0.42117123
1	Asap1	1.78E-47	0.483624169
1	P4ha1	1.88E-47	0.474821644
1	Gpx3	1.98E-47	0.416138408
1	Tubb3	3.16E-47	0.434642852
1	Eprs	3.45E-47	0.501004579
1	Degs1	5.86E-47	0.45152751
1	Serf1	7.66E-47	0.472109988
1	Fam162a	1.02E-46	0.567765463
1	Psma2	1.43E-46	0.413251186
1	Sqstm1	2.02E-46	0.502851899
1	Ssr4	2.08E-46	0.430209464
1	Sod2	2.59E-46	0.497139463
1	Adprh	3.19E-46	0.426622906
1	Tmem167	3.38E-46	0.45902322
1	Pkm	3.69E-46	0.459541346
1	Rassf1	4.87E-46	0.45922825
1	Ch25h	5.42E-46	0.431411425
1	Manf	5.76E-46	0.49711349

Table S2:Cluster specific genes identified by the differential analysis in the Organoid.

cluster	gene	p value	fold change *
1	Fabp4	7.89E-46	0.531301858
1	Fibin	9.88E-46	0.487731782
1	Vimp	1.13E-45	0.49756459
1	Smad7	1.27E-45	0.472749753
1	11-Sep	1.35E-45	0.484369326
1	Prnp	1.75E-45	0.512339094
1	Nov	2.04E-45	0.637652076
1	Myeov2	3.34E-45	0.446735575
1	Eif1b	6.45E-45	0.458656425
1	Serpinb6a	1.28E-44	0.476415944
1	Gng12	4.86E-44	0.478216748
1	Apoe	6.47E-44	0.87790581
1	Rsrp1	6.93E-44	0.459391602
1	Hexb	9.23E-44	0.445282234
1	Penk	1.04E-43	0.552939106
1	Vegfa	1.13E-43	0.509582688
1	Gnai2	1.80E-43	0.442143179
1	2310022B05Rik	3.67E-43	0.406908254
1	Ddb1	4.78E-43	0.475028638
1	Cnn1	5.38E-43	0.490645169
1	Mrpl20	6.25E-43	0.436900177
1	Pnp	9.28E-43	0.46223673
1	Ndufb6	9.94E-43	0.428754384
1	Sh3bgrl3	1.34E-42	0.445811275
1	Htra1	1.74E-42	0.514468292
1	Lyz2	2.32E-42	0.48415003
1	1110065P20Rik	2.52E-42	0.435959721
1	Tln1	2.78E-42	0.417196644
1	Eno1	3.46E-42	0.466724324
1	Arl2	3.88E-42	0.44306657
1	Aprt	5.16E-42	0.434477144
1	Vat1	2.77E-41	0.432418016
1	Cdkn2b	6.14E-41	0.558795865
1	Sepp1	1.89E-40	0.428516581
1	Sar1a	2.97E-40	0.434606856
1	Aga	4.19E-40	0.442429217
1	Tomm5	6.05E-40	0.436317574
1	Tspan6	1.04E-39	0.425631142
1	Serpine2	1.14E-39	0.582117256
1	Morf4l2	1.35E-39	0.441626351
1	Pofut2	1.90E-39	0.417539194

Table S2:Cluster specific genes identified by the differential analysis in the Organoid.

cluster	gene	p value	fold change *
1	Txndc15	2.06E-39	0.408300866
1	Cyb5r3	4.69E-39	0.426444868
1	Ccnd3	4.89E-39	0.459487862
1	Anxa5	5.69E-39	0.441092673
1	Rexo2	3.13E-38	0.405338202
1	Ahnak	5.47E-38	0.486661547
1	Cd44	5.47E-38	0.419003314
1	Mmp11	6.11E-38	0.439886159
1	Srm	9.26E-38	0.437280885
1	Nenf	1.06E-37	0.400002678
1	Prelp	1.31E-37	0.462424076
1	Clptm1l	1.78E-37	0.403065652
1	Sec13	2.35E-37	0.406520426
1	Marcks	2.37E-37	0.41393038
1	Dbi	2.47E-37	0.41447517
1	Igfbp2	1.36E-36	0.885806349
1	2-Mar	1.38E-36	0.430566984
1	Il6	1.74E-36	0.419140515
1	Zwint	2.79E-36	0.433832954
1	Pdlim7	1.20E-35	0.411089934
1	Cnn3	1.62E-35	0.4188935
1	Fzd1	1.44E-34	0.471411118
1	Rnase4	1.69E-34	0.426418042
1	Cxcl1	1.79E-33	0.84908332
1	Lamb1	5.37E-33	0.449672493
1	Lum	1.26E-32	0.675943428
1	Serpinb1a	1.38E-32	0.637764833
1	C3	1.13E-31	0.434114017
1	Zfp36	1.42E-30	0.49647374
1	Sfrp2	1.67E-30	0.452877944
1	Sgk1	3.35E-29	0.57826925
1	Itgb5	4.49E-29	0.402894078
1	Cdkn1c	1.15E-28	0.460080665
1	Csrp2	9.21E-28	0.436820857
1	Higd1a	1.24E-26	0.408467598
1	Dlk1	1.88E-26	0.501318824
1	Ddit3	5.52E-26	0.401106626
1	Hist1h2bc	5.20E-25	0.535620829
1	Ptn	9.50E-24	0.408202671
1	Gadd45a	2.15E-23	0.410421817
1	Ifi27l2a	7.61E-21	0.401332159

Table S2:Cluster specific genes identified by the differential analysis in the Organoid.

cluster	gene	p value	fold change *
2	Stmn1	2.79E-52	1.585766778
2	Tuba1b	2.10E-43	1.272324602
2	Ccnb1	2.67E-43	0.952595938
2	Smc2	3.26E-43	1.168850342
2	Ccna2	3.70E-43	0.785733773
2	Birc5	6.13E-43	1.513741243
2	Cdca8	1.43E-42	1.066515736
2	Cenpa	2.20E-41	1.456242429
2	Ube2c	7.26E-41	1.539011437
2	Cks1b	1.87E-39	1.220824957
2	Spc24	2.59E-39	0.99670743
2	Cdca3	1.95E-38	0.954177835
2	Pbk	2.00E-38	0.793156593
2	2810417H13Rik	3.18E-38	1.289902728
2	Cdc20	1.00E-37	1.088339964
2	Lockd	8.05E-37	0.998941324
2	Ccnb2	9.17E-36	1.03999761
2	2700094K13Rik	1.05E-35	1.157561752
2	Cenpm	2.93E-35	0.937468975
2	Cenpw	9.93E-35	0.819405715
2	Pttg1	1.08E-34	1.356035737
2	Lgals1	4.44E-32	1.176506565
2	Ckap2	2.05E-31	0.476932444
2	Cdk1	1.12E-30	1.23689341
2	Tubb5	1.75E-30	1.106031819
2	Tpx2	7.84E-30	0.722553122
2	Ube2t	1.60E-29	0.563126247
2	Racgap1	2.28E-29	0.860437078
2	Aurka	4.96E-29	0.659383597
2	Cks2	5.46E-29	0.998715668
2	Spc25	1.13E-28	0.694522307
2	Knstrn	1.25E-28	0.752741555
2	Cenpf	1.46E-28	0.801258035
2	Fam64a	1.49E-28	0.685170425
2	Cenph	1.75E-28	0.673421819
2	Top2a	1.69E-27	1.107537763
2	Cdkn3	1.71E-27	0.659893869
2	Ran	1.47E-26	0.788746939
2	Mns1	6.04E-26	0.46265413
2	Ckap2l	1.05E-25	0.443355811
2	Anln	1.15E-24	0.503316576

Table S2:Cluster specific genes identified by the differential analysis in the Organoid.

cluster	gene	p value	fold change *
2	Hmmr	1.70E-24	0.496384219
2	Nusap1	4.13E-24	0.497628422
2	Tubb6	4.91E-24	1.052864698
2	Cenpe	7.22E-24	0.621477012
2	Rad51	1.10E-23	0.622486414
2	Rrm2	1.24E-21	0.690089742
2	Cdca2	1.32E-21	0.494227514
2	Ska1	2.22E-21	0.414970967
2	Atad2	2.24E-21	0.534238417
2	Ndc80	2.56E-21	0.52075181
2	Smc4	2.72E-21	0.980901862
2	Ncapd2	7.16E-21	0.491201038
2	Hmgb2	8.09E-21	1.101706048
2	Kif2c	1.39E-20	0.482224271
2	Tk1	1.45E-20	0.730760412
2	Tacc3	2.54E-20	0.480854479
2	Dhfr	5.90E-20	0.613209948
2	Gins2	1.09E-19	0.750168397
2	Dtymk	3.77E-19	0.807825624
2	Pmf1	8.09E-19	0.696330219
2	Dbf4	2.51E-18	0.604570554
2	Hmgn2	3.48E-18	0.502337231
2	Bgn	6.71E-18	0.576337309
2	Dek	6.97E-18	0.826537241
2	H2afv	1.33E-17	0.690212013
2	Kifc1	1.76E-17	0.441721177
2	Asf1b	3.36E-17	0.446249589
2	Ncl	4.11E-17	0.715498297
2	Dut	4.13E-17	0.878814261
2	H2afz	5.15E-17	0.710121185
2	Tm4sf1	6.00E-17	0.522233944
2	Slc29a1	8.03E-17	0.543165024
2	Mcm3	9.01E-17	0.488320307
2	Serpinf1	1.16E-16	0.416228889
2	Plk1	1.42E-16	0.418384087
2	Tyms	1.65E-16	0.737872629
2	Nsg1	2.79E-16	0.448553
2	Kif23	3.42E-16	0.486128553
2	Vim	4.38E-16	0.867177059
2	Aurkb	4.68E-16	0.489862582
2	Wisp1	5.94E-16	0.538951276

Table S2:Cluster specific genes identified by the differential analysis in the Organoid.

cluster	gene	p value	fold change *
2	Srm	6.13E-16	0.786335933
2	Psat1	6.56E-16	0.694833512
2	Prkg2	9.03E-16	0.748702671
2	Cald1	1.01E-15	0.555400832
2	Kif20a	1.11E-15	0.429970053
2	Gmnn	1.91E-15	0.535056318
2	Nt5dc2	2.55E-15	0.602935161
2	Rpa2	3.36E-15	0.525513371
2	2700029M09Rik	4.03E-15	0.524695555
2	Cmtm3	4.33E-15	0.432653167
2	Bcat1	4.37E-15	0.495871575
2	Prc1	1.11E-14	0.458393648
2	Ranbp1	2.19E-14	0.750817198
2	Serpinh1	2.70E-14	0.48680713
2	Mthfd2	2.76E-14	0.714289501
2	Tcf19	4.65E-14	0.451262768
2	Mki67	6.84E-14	0.41566602
2	Pcna	1.09E-13	0.607921225
2	Ezh2	1.21E-13	0.484396323
2	Tipin	1.94E-13	0.550467719
2	Hn1	2.04E-13	0.665689494
2	Dnajc9	2.12E-13	0.43045014
2	Lsm2	2.18E-13	0.625644588
2	Usp1	3.12E-13	0.472059505
2	Anp32b	5.11E-13	0.640099092
2	Rad21	5.47E-13	0.499535998
2	Rrm1	6.52E-13	0.405792962
2	Prrx1	7.14E-13	0.486435803
2	Pkm	1.02E-12	0.590196703
2	Rangap1	1.15E-12	0.534614015
2	Prdx4	1.15E-12	0.736429675
2	Ak6	1.33E-12	0.561419787
2	Tmpo	3.11E-12	0.494778455
2	Tuba1a	3.54E-12	0.704972594
2	Wdr12	3.99E-12	0.407733172
2	Tubb3	9.28E-12	0.43127579
2	Txn1	9.87E-12	0.497245728
2	Steap1	1.28E-11	0.495926924
2	Hmga2	1.73E-11	0.701439264
2	Eif4ebp1	1.85E-11	0.629670205
2	D030056L22Rik	1.98E-11	0.424581081

Table S2:Cluster specific genes identified by the differential analysis in the Organoid.

cluster	gene	p value	fold change *
2	Terf1	2.04E-11	0.459314483
2	Nop56	2.44E-11	0.638437302
2	Nudcd2	2.44E-11	0.550805329
2	Fbln2	2.71E-11	0.457236923
2	Alyref	2.74E-11	0.542218225
2	Yaf2	3.39E-11	0.588616034
2	Msn	4.27E-11	0.438692326
2	Peak1	4.39E-11	0.470415227
2	Cdkn2d	7.01E-11	0.463006004
2	Mif	7.37E-11	0.632420689
2	Cox7a2	9.92E-11	0.496840831
2	Psip1	1.07E-10	0.591587084
2	Oaf	1.31E-10	0.452864648
2	Pin1	1.57E-10	0.546954664
2	Hat1	1.74E-10	0.497954393
2	Syce2	2.03E-10	0.474399981
2	Cmc2	2.36E-10	0.565230683
2	Shmt2	2.38E-10	0.597144286
2	Tmem97	3.63E-10	0.422075063
2	Phgdh	3.78E-10	0.536877877
2	Nhp2	5.66E-10	0.580347989
2	Mcm6	7.33E-10	0.459829418
2	Ssr1	9.92E-10	0.435525962
2	Mrpl18	1.02E-09	0.486740618
2	Pole4	1.10E-09	0.492286111
2	Lsm3	1.19E-09	0.440777205
2	Ppa1	1.33E-09	0.436734125
2	Pabpc1	1.40E-09	0.487542749
2	Ndufaf2	1.42E-09	0.430626644
2	Ak2	1.61E-09	0.544310589
2	Ncaph2	1.65E-09	0.474451663
2	Cth	1.75E-09	0.552855603
2	Dkc1	1.87E-09	0.494909264
2	Rhoc	2.15E-09	0.44958788
2	Nucks1	2.18E-09	0.492308823
2	Pole3	2.50E-09	0.434473534
2	Ssrp1	2.92E-09	0.515426938
2	Clic4	3.36E-09	0.406417529
2	Spp1	3.41E-09	0.786362303
2	Aars	3.65E-09	0.538684165
2	Nsmce2	4.22E-09	0.457808436

Table S2:Cluster specific genes identified by the differential analysis in the Organoid.

cluster	gene	p value	fold change *
2	Mrpl13	4.84E-09	0.46245379
2	Banf1	4.86E-09	0.507605102
2	Emp3	5.91E-09	0.554428863
2	Fam92a	5.96E-09	0.488004105
2	Angptl6	8.23E-09	0.450541333
2	Dctpp1	8.53E-09	0.498241355
2	Cep57	9.39E-09	0.456878779
2	Psph	1.01E-08	0.569263126
2	Nme1	1.08E-08	0.537633072
2	Hmga1	1.32E-08	0.427230551
2	Anapc11	1.59E-08	0.520435313
2	Fkbp2	1.99E-08	0.452830411
2	Ppil1	2.04E-08	0.421018684
2	Ddx39	2.06E-08	0.498274762
2	Rrp15	2.07E-08	0.436033094
2	Fkbp1a	2.37E-08	0.462411023
2	Snrpd1	2.82E-08	0.479566892
2	Ebna1bp2	2.95E-08	0.405784597
2	Orc6	3.86E-08	0.477967006
2	Cbx5	6.32E-08	0.421981969
2	Cox6a2	6.66E-08	0.404567751
2	Hmgb1	6.99E-08	0.500103796
2	Cnih1	7.13E-08	0.435193131
2	Serbp1	8.90E-08	0.462882893
2	H2afx	9.14E-08	0.579839148
2	G3bp1	1.14E-07	0.494074531
2	Ddx18	1.17E-07	0.415578432
2	Pgp	1.17E-07	0.444779737
2	Polr2f	1.43E-07	0.449282739
2	Eno1	1.54E-07	0.461508212
2	Rps19bp1	1.72E-07	0.430370178
2	Ccnd1	1.94E-07	0.488514271
2	Manf	3.10E-07	0.446479797
2	Dcn	3.72E-07	0.459339096
2	Zcrb1	4.00E-07	0.41760872
2	Phf5a	4.27E-07	0.494359586
2	Mrpl49	4.54E-07	0.427951195
2	Ccl7	4.74E-07	0.401557485
2	Cyb5r1	4.98E-07	0.432072392
2	Mrto4	6.49E-07	0.438072545
2	Eef1e1	6.59E-07	0.439546288

Table S2:Cluster specific genes identified by the differential analysis in the Organoid.

cluster	gene	p value	fold change *
2	Zfos1	6.72E-07	0.527722845
2	Srsf7	1.42E-06	0.511249878
2	Zfp706	1.59E-06	0.416566722
2	Gapdh	1.63E-06	0.421677802
2	Cnih4	1.69E-06	0.443960488
2	Timm17a	1.94E-06	0.417976719
2	Hspa9	2.16E-06	0.451721534
2	Dap	2.49E-06	0.42699002
2	Dlk1	3.09E-06	0.52806399
2	Mrps30	4.12E-06	0.428794958
2	Lrrc59	4.95E-06	0.412620308
2	Nap1l1	6.23E-06	0.401929062
2	Lyar	6.72E-06	0.444762068
2	Gars	7.52E-06	0.402341493
2	Tpm2	9.32E-06	0.477991853
2	Mrpl51	9.68E-06	0.420881229
2	Arpp19	1.42E-05	0.404682031
2	Asns	2.47E-05	0.430844236
2	Tspo	4.85E-05	0.452533903
2	Tpm1	0.001126	0.435035359
2	Ptn	0.009524	0.418968182
3	Elof1	1.63E-115	2.047977978
3	Chchd10	1.67E-93	2.55615977
3	Ift172	3.17E-93	2.593299503
3	1700016K19Rik	1.17E-76	2.555619536
3	Cfap126	2.26E-76	2.416804586
3	Tppp3	4.79E-73	2.724652206
3	Ccdc153	3.45E-72	2.579859463
3	1700088E04Rik	8.05E-71	1.921528797
3	Tmem212	4.96E-68	2.574395408
3	Pifo	5.42E-66	2.359817497
3	Mlf1	4.04E-65	2.075547331
3	Meig1	6.22E-65	2.307228694
3	Lrrc51	6.43E-63	1.918428137
3	1700007K13Rik	6.48E-61	2.102926924
3	Fam183b	7.06E-61	2.488691138
3	Tmem107	1.40E-60	1.752096808
3	Ift43	1.24E-58	1.589900565
3	Ccdc189	7.05E-58	1.793031492
3	Rsph1	8.55E-58	2.008079083
3	Arhgdig	1.24E-56	1.772585561

Table S2:Cluster specific genes identified by the differential analysis in the Organoid.

cluster	gene	p value	fold change *
3	1110004E09Rik	1.47E-56	1.535835502
3	Prr29	1.14E-55	1.918647146
3	Tctex1d4	1.03E-54	1.665581873
3	Cfap206	9.21E-54	1.422667294
3	Spef1	2.05E-53	1.67707032
3	2410004P03Rik	2.05E-53	1.648694289
3	AU040972	2.30E-53	3.032962153
3	Bola1	4.36E-51	1.416952234
3	1700026L06Rik	3.71E-50	1.830329597
3	Dpcd	1.88E-48	1.455534976
3	BC051019	6.55E-48	1.540618792
3	Foxj1	1.42E-47	1.842427318
3	Erich2	2.78E-47	1.631885808
3	Morn5	6.67E-47	1.653555242
3	Chchd6	3.67E-45	1.446489889
3	Nme5	1.53E-44	1.555206465
3	Fam92b	1.58E-44	1.552148094
3	Capsl	3.75E-44	1.675687207
3	1110017D15Rik	5.40E-44	1.658491714
3	Ccdc113	8.93E-44	1.530073382
3	Cetn4	1.87E-42	1.500036607
3	Cep83	2.43E-41	1.472853986
3	Ccpg1os	2.45E-41	1.403310909
3	Ccdc34	2.68E-41	1.536010899
3	Ezr	8.20E-41	1.586184293
3	4933434E20Rik	5.28E-40	1.422748967
3	Gtsf1l	5.75E-40	1.270765625
3	Tekt1	2.63E-39	1.51627138
3	Gtf2b	4.03E-39	1.328391893
3	Smim5	4.75E-39	1.582841457
3	Nudc	7.42E-39	1.289877856
3	Rsph9	1.30E-38	1.370566528
3	1700013F07Rik	1.36E-38	1.330407391
3	Mycbp	1.46E-38	1.314509373
3	Sntn	4.86E-38	1.346895419
3	Lrrc23	7.97E-38	1.31890668
3	Cfap77	1.17E-37	1.129633657
3	Dnali1	1.72E-37	1.140897496
3	Lrrc48	1.85E-37	1.382642227
3	Cfap36	3.72E-37	1.312704543
3	Cfap52	4.99E-37	1.211945927

Table S2:Cluster specific genes identified by the differential analysis in the Organoid.

cluster	gene	p value	fold change *
3	Dnah12	1.19E-36	1.527263581
3	Ift22	1.33E-36	1.293385331
3	Ccdc146	1.84E-36	1.110296964
3	Calm3	1.98E-36	1.257830087
3	2610028H24Rik	2.50E-36	1.395826322
3	Gde1	5.30E-36	1.31707006
3	Dnajb13	9.27E-36	1.081795418
3	1700007G11Rik	2.43E-35	1.227063803
3	Cib1	2.55E-35	1.310219747
3	Mapk15	5.50E-35	0.978310241
3	Ccdc17	5.59E-35	1.015842281
3	Ndufaf3	1.38E-34	1.282593114
3	Cyp2s1	1.55E-34	1.133007823
3	Gm867	2.03E-34	1.27825278
3	1110032A03Rik	4.61E-34	1.232522852
3	Ccdc39	5.48E-34	1.372106505
3	Crip2	5.85E-34	1.401719175
3	Fank1	7.04E-34	1.047397175
3	Tspan1	7.60E-34	1.672277071
3	Tekt4	9.92E-34	1.060344452
3	Eml2	1.12E-33	1.272530421
3	Ift46	1.24E-33	1.195717097
3	Mns1	1.85E-33	1.069613341
3	Cldn3	4.91E-33	1.644314643
3	Tm4sf1	5.24E-33	1.843686359
3	Vpreb3	1.79E-32	1.654192823
3	Cfap45	2.68E-32	1.166602772
3	Ctxn1	3.64E-32	1.274897284
3	Ccdc181	3.88E-32	1.297080978
3	Fam47e	4.32E-32	1.006829354
3	Cetn2	1.02E-31	1.193806125
3	Kif19a	2.78E-31	1.135981993
3	Ccdc96	3.19E-31	0.937706242
3	Smim22	4.65E-31	1.37469908
3	Acox2	5.63E-31	1.207835194
3	Smim6	7.78E-31	1.326878146
3	Basp1	9.52E-31	1.370678576
3	Pih1d2	9.94E-31	1.069538755
3	1700001C02Rik	1.14E-30	1.212266806
3	Tubb4b	1.65E-30	1.307449741
3	Ttc25	1.74E-30	0.994356556

Table S2:Cluster specific genes identified by the differential analysis in the Organoid.

cluster	gene	p value	fold change *
3	Spef2	2.42E-30	1.035073279
3	Fbxo36	5.47E-30	1.236414633
3	Saxo2	5.49E-30	1.172057347
3	Ttc16	6.79E-30	0.8661098
3	Cxcl17	7.67E-30	1.76963025
3	Uckl1os	9.61E-30	1.193986832
3	Lrrc71	1.16E-29	0.700925401
3	Cyb561	1.52E-29	1.218875062
3	Gas8	1.54E-29	1.129303785
3	Ubxn11	2.84E-29	1.044381849
3	Morn3	3.07E-29	0.917159682
3	Dnah6	3.65E-29	0.776565498
3	Enkur	4.32E-29	1.0516868
3	Lrrc46	4.96E-29	1.034541268
3	Ccdc65	5.01E-29	0.931811355
3	B9d1	5.05E-29	1.193620354
3	Ccdc78	5.94E-29	0.902640644
3	Dnaaf3	6.87E-29	1.001640869
3	Sri	8.30E-29	1.037906164
3	Cfap70	1.06E-28	0.951391331
3	Dnajc15	1.11E-28	1.102753447
3	Gm29538	2.81E-28	0.965154154
3	3300002A11Rik	7.73E-28	1.378196937
3	Spag17	9.55E-28	0.725697826
3	Ppfia3	1.34E-27	1.161196786
3	Ccdc40	2.25E-27	0.905702365
3	Mok	2.41E-27	1.048446863
3	Cystm1	4.37E-27	1.281472093
3	1810037I17Rik	1.18E-26	1.122935943
3	Iqcg	1.73E-26	0.820459527
3	Fam179a	2.88E-26	0.787998468
3	Tuba1a	3.63E-26	1.411634349
3	Nhlrc4	6.30E-26	0.547354274
3	Ak9	7.59E-26	0.733430747
3	Dnah9	1.04E-25	0.798879757
3	4931406C07Rik	1.19E-25	1.104691698
3	Cby1	1.30E-25	1.12196023
3	Wdr60	1.31E-25	1.093250674
3	Mak	2.56E-25	0.741675295
3	Ift27	3.39E-25	1.098916517
3	1700028P14Rik	3.75E-25	0.860546874

Table S2:Cluster specific genes identified by the differential analysis in the Organoid.

cluster	gene	p value	fold change *
3	Pacrg	4.52E-25	0.924119259
3	Dynlrb2	1.07E-24	1.594440413
3	Znhit1	1.13E-24	0.968224353
3	Swi5	1.24E-24	0.965024636
3	Ttc21a	1.42E-24	0.805943817
3	Vwa3a	1.61E-24	0.567259161
3	Rabl2	1.75E-24	1.040654438
3	Dusp14	1.89E-24	1.213179609
3	Cdhr4	2.25E-24	0.954541757
3	Eif2b2	2.62E-24	1.052555626
3	Cfap44	2.88E-24	0.581339123
3	Bphl	4.41E-24	1.043009289
3	Dalrd3	5.39E-24	1.137495143
3	Cd24a	5.82E-24	1.430247705
3	Tmc5	6.15E-24	0.870571835
3	Ak7	6.84E-24	0.708053092
3	Ift88	8.66E-24	0.916493732
3	Zmynd12	1.95E-23	0.724828563
3	Lgals3	2.54E-23	1.725386814
3	Fam81b	3.41E-23	0.778928621
3	1700037C18Rik	4.37E-23	0.91264269
3	Ccdc103	4.43E-23	0.933054808
3	Aldh1a1	4.67E-23	1.338606919
3	Ift74	4.68E-23	1.000240575
3	Gm15402	5.77E-23	0.701109909
3	Ruvbl2	1.12E-22	1.052864378
3	Lhb	1.13E-22	0.504852229
3	Bbof1	1.13E-22	0.844404607
3	Las1l	1.39E-22	1.12898068
3	Myh14	1.40E-22	1.214362248
3	Agr2	1.54E-22	1.643386939
3	1700029J07Rik	1.67E-22	0.844078765
3	Gpx4	1.73E-22	0.887171593
3	Ankrd42	2.18E-22	0.887363731
3	Dydc2	2.35E-22	0.661622155
3	Kcnh3	3.57E-22	0.743439807
3	Ccdc151	4.57E-22	0.676805384
3	Enkd1	4.76E-22	0.913638892
3	Wdr66	5.55E-22	0.628809873
3	Fhad1	8.53E-22	0.74265168
3	Rsph4a	1.51E-21	0.53847201

Table S2:Cluster specific genes identified by the differential analysis in the Organoid.

cluster	gene	p value	fold change *
3	Armc4	1.86E-21	0.704831483
3	Cep126	2.07E-21	0.759926166
3	Kif3a	2.55E-21	0.990367006
3	Wdr63	4.75E-21	0.490862715
3	Ino80b	9.42E-21	1.016069384
3	Adam8	1.48E-20	0.824212916
3	Cfap69	1.55E-20	0.861052818
3	Hydin	1.87E-20	0.527089609
3	Hspa2	2.10E-20	1.019753777
3	Tomm7	2.20E-20	0.862220678
3	Hdc	2.44E-20	0.871128733
3	Sptlc3	2.61E-20	0.75957123
3	Clic3	3.18E-20	1.050222611
3	BC022687	3.96E-20	0.93067376
3	Dnah3	5.65E-20	0.572878509
3	Arhgef28	6.96E-20	1.016745079
3	Cfap46	9.18E-20	0.442327194
3	Lrriq1	1.00E-19	0.659227245
3	Gng5	1.11E-19	0.807488947
3	Ifitm1	1.34E-19	1.7730778
3	Efhc1	1.35E-19	0.659413006
3	Cbr2	1.60E-19	1.662611877
3	Ankrd54	1.62E-19	0.994300242
3	Dnah11	4.43E-19	0.492770916
3	Cfap100	4.56E-19	0.829870129
3	Fam216a	4.67E-19	0.982817142
3	Cmtm8	5.25E-19	0.983967835
3	Dnaaf1	6.53E-19	0.605187539
3	Adipor2	7.75E-19	0.982938076
3	Maats1	8.06E-19	0.504746447
3	Kif9	1.03E-18	0.618470576
3	Cisd3	1.05E-18	1.009805793
3	Cfap161	1.16E-18	0.452593247
3	Ttc39a	1.47E-18	0.68065919
3	Akip1	2.04E-18	0.921711542
3	Oscp1	2.57E-18	0.822974071
3	Prr15l	2.79E-18	0.930645218
3	Krt8	3.00E-18	1.223037349
3	Tctex1d2	3.09E-18	0.914685892
3	Rsph14	4.09E-18	0.407683643
3	2200002D01Rik	4.09E-18	1.138473878

Table S2:Cluster specific genes identified by the differential analysis in the Organoid.

cluster	gene	p value	fold change *
3	Cds1	4.49E-18	0.733166336
3	Cnp	4.91E-18	0.925169021
3	Strbp	4.92E-18	0.973030577
3	Ulk4	5.11E-18	0.671305458
3	Ctsh	5.42E-18	1.014656539
3	Lrrc56	6.33E-18	0.656234419
3	Lrrc10b	7.34E-18	0.631042015
3	Sec14l3	7.60E-18	0.687244387
3	Ppil6	8.44E-18	0.756716147
3	Ccdc74a	1.08E-17	0.628338112
3	Dcxr	1.10E-17	1.313506649
3	Ndufb5	1.16E-17	0.814403923
3	2010109I03Rik	1.45E-17	0.632313853
3	Fam161b	1.63E-17	0.584780595
3	Gm42722	1.76E-17	0.542989755
3	Ccdc180	1.80E-17	0.490659078
3	Ldlrad1	1.80E-17	0.476904532
3	Sys1	2.25E-17	0.866480231
3	Catip	2.31E-17	0.612764975
3	Mt1	3.08E-17	1.085645687
3	Traf3ip1	3.16E-17	0.800000152
3	Spa17	3.49E-17	0.846999999
3	Adck4	4.36E-17	0.894157246
3	Rfc2	4.79E-17	0.900460632
3	Dusp18	4.97E-17	0.919529278
3	6820408C15Rik	6.65E-17	0.677079432
3	Efcab10	6.81E-17	0.733604712
3	Cntrl	7.13E-17	0.796857368
3	Mdh1b	8.49E-17	0.417963943
3	Nek11	8.98E-17	0.575550452
3	Nphp1	1.03E-16	0.846064922
3	Dnah1	1.05E-16	0.433869858
3	Pbx4	1.15E-16	0.811259431
3	Wdr54	1.43E-16	0.851795846
3	Riiad1	1.46E-16	0.794244906
3	Ribc1	1.55E-16	0.616397021
3	Ckap2l	1.76E-16	0.912242669
3	Dnaaf2	1.93E-16	0.826097467
3	Cfap20	2.00E-16	0.847095357
3	Gm26717	2.81E-16	0.65582891
3	Ift81	2.81E-16	0.848356212

Table S2:Cluster specific genes identified by the differential analysis in the Organoid.

cluster	gene	p value	fold change *
3	Pcnxl4	2.92E-16	0.836295237
3	Syt5	2.93E-16	0.642432905
3	Slc9a3r1	3.92E-16	0.953974227
3	Ccdc67	4.04E-16	0.869960697
3	Fam227a	4.50E-16	0.454908661
3	Tsnaxip1	4.71E-16	0.577948246
3	Zmynd10	6.51E-16	0.813396191
3	B230118H07Rik	7.05E-16	0.916405936
3	Bbs7	7.55E-16	0.599225702
3	Fam134b	9.60E-16	0.959755058
3	Ppp5c	9.71E-16	0.852684509
3	Nat9	1.16E-15	0.868097135
3	Styxl1	1.18E-15	0.620995136
3	Wdr34	1.30E-15	0.832826267
3	Akap14	1.35E-15	0.565976116
3	Ccdc60	1.35E-15	0.413920135
3	Lrwd1	1.37E-15	0.87335647
3	Dpy30	1.40E-15	0.833960962
3	Ddr1	1.65E-15	0.948705254
3	Morn2	2.09E-15	0.92213713
3	Prom1	2.36E-15	0.928208248
3	Cep290	2.56E-15	0.894243462
3	D430036J16Rik	2.74E-15	0.452763797
3	1700024G13Rik	2.79E-15	0.718625259
3	Hes6	2.84E-15	1.223564647
3	Hipk1	2.88E-15	0.829084007
3	Smkr-ps	3.19E-15	0.421628119
3	Sugt1	3.30E-15	0.815359795
3	Pglyrp1	3.56E-15	1.156578507
3	Ccdc173	3.70E-15	0.700056004
3	Wdr78	3.91E-15	0.623983254
3	Rfk	4.05E-15	0.87329244
3	Krt18	4.26E-15	1.166202399
3	Josd2	4.36E-15	0.817550371
3	Acyp1	4.39E-15	0.947084946
3	1810008I18Rik	4.64E-15	0.425392748
3	D430042O09Rik	4.96E-15	0.921859686
3	Dynlt1f	5.12E-15	0.858453315
3	Ina	5.92E-15	0.704380527
3	Lrrc73	7.27E-15	0.731674268
3	2210407C18Rik	7.35E-15	0.762586142

Table S2:Cluster specific genes identified by the differential analysis in the Organoid.

cluster	gene	p value	fold change *
3	1810043G02Rik	1.11E-14	0.876633513
3	Hspa4l	1.16E-14	1.154845183
3	1700003E16Rik	1.22E-14	0.405740896
3	Lekr1	1.63E-14	0.765024236
3	Pkig	1.70E-14	0.819773836
3	Ccdc30	2.00E-14	0.566184588
3	Gm33050	2.31E-14	0.447017529
3	Cfap57	3.00E-14	0.431312325
3	Fhit	3.24E-14	0.641440304
3	Snrnp25	3.54E-14	0.783112274
3	Ccdc191	4.07E-14	0.842627154
3	Mdm1	6.44E-14	0.762898576
3	Psmd9	9.82E-14	0.798770798
3	Iqck	1.08E-13	0.517559045
3	Odf2l	1.16E-13	0.810526413
3	Spag6l	1.22E-13	0.553024953
3	1700086L19Rik	1.40E-13	0.539551722
3	BC005624	1.61E-13	0.785824005
3	Nudt4	2.92E-13	0.881823529
3	Ruvbl1	3.03E-13	0.758261269
3	Wdr35	4.07E-13	0.819608678
3	B9d2	4.25E-13	0.801002464
3	Ndufab1	5.01E-13	0.761743314
3	Fbxo32	6.37E-13	0.941711911
3	Ppp1r16a	6.38E-13	0.684515877
3	Mrpl34	6.52E-13	0.694598994
3	Ift57	7.89E-13	0.847731828
3	Efcab11	8.56E-13	0.619419257
3	Rph3al	9.00E-13	0.50763156
3	Med31	9.35E-13	0.73994836
3	Wwc1	1.49E-12	0.787953827
3	Glod4	1.61E-12	0.817614935
3	Ccp110	2.00E-12	0.574267448
3	Tmem231	2.01E-12	0.53854261
3	Cep131	2.07E-12	0.572332861
3	Elf3	2.19E-12	0.914744947
3	Lrrc45	2.47E-12	0.803199333
3	Pcyt1b	2.64E-12	0.543005767
3	Bag1	2.72E-12	0.673380114
3	Acot13	2.73E-12	0.792367916
3	PISD	3.37E-12	0.902733104

Table S2:Cluster specific genes identified by the differential analysis in the Organoid.

cluster	gene	p value	fold change *
3	Ppp1r14c	3.44E-12	0.740632226
3	Mea1	3.69E-12	0.669184941
3	Gga1	3.72E-12	0.767377028
3	Ahsa1	3.74E-12	0.776741044
3	Serpina9	4.86E-12	0.955146999
3	Fam167a	5.09E-12	0.518309203
3	Fbxo16	5.39E-12	0.434246705
3	Pllp	5.53E-12	0.617343539
3	Ly6c1	6.38E-12	1.345831691
3	Arl6	6.81E-12	0.776118563
3	Rassf7	7.23E-12	0.806375976
3	Zc2hc1c	7.38E-12	0.545806034
3	Prr13	8.07E-12	0.768017576
3	Rilpl2	8.63E-12	0.728987537
3	Gm16537	1.50E-11	0.456584011
3	Nelfe	1.57E-11	0.715750451
3	Thop1	1.72E-11	0.74494959
3	Cmbl	1.77E-11	0.650626481
3	Cluap1	1.81E-11	0.754808005
3	Bad	2.07E-11	0.605683744
3	Churc1	2.66E-11	0.785860357
3	Dnal4	3.09E-11	0.764078932
3	Spire2	3.21E-11	0.478290505
3	Rwdd3	4.18E-11	0.482691191
3	Ift20	4.47E-11	0.750111429
3	Gm17586	6.58E-11	0.487440643
3	Mrpl24	6.59E-11	0.66972194
3	Nudt17	7.00E-11	0.459162836
3	Diras2	7.05E-11	0.474181248
3	Mrpl57	7.45E-11	0.716371097
3	Rpgrip1l	7.60E-11	0.595345982
3	Lrrc34	7.91E-11	0.444579701
3	Ift52	8.01E-11	0.692005005
3	Ttc36	8.12E-11	0.522332749
3	Gdpd1	8.48E-11	0.693848628
3	Ogg1	1.02E-10	0.571855185
3	Ndufc2	1.04E-10	0.677792318
3	Pla2g16	1.26E-10	0.721613784
3	Mrps17	1.31E-10	0.688969258
3	Znhit2	1.37E-10	0.635600908
3	Ceacam1	1.38E-10	0.793017203

Table S2:Cluster specific genes identified by the differential analysis in the Organoid.

cluster	gene	p value	fold change *
3	Isyna1	1.64E-10	0.810404443
3	Cd82	1.74E-10	0.705510359
3	Rfx2	1.90E-10	0.530431903
3	Spint2	2.19E-10	0.561831168
3	Gm11992	2.26E-10	0.439519311
3	Polr3k	2.72E-10	0.716624448
3	Pdxk	2.82E-10	0.659745187
3	Celsr1	2.83E-10	0.549064387
3	Tmem17	2.88E-10	0.573198715
3	Abcb6	3.04E-10	0.507802948
3	Wars	4.72E-10	0.666464211
3	Ly6g6c	6.09E-10	0.741245022
3	Cdpf1	6.14E-10	0.690747157
3	Fam84b	6.64E-10	0.489901663
3	Myo5b	6.82E-10	0.416632488
3	Shisa8	6.86E-10	0.819759825
3	Gm1673	9.74E-10	0.707878087
3	Odf2	1.00E-09	0.606775099
3	Spns1	1.13E-09	0.70875836
3	Scd2	1.19E-09	0.746364168
3	Carkd	1.20E-09	0.653057413
3	Phtf1	1.22E-09	0.687683088
3	Sirt3	1.43E-09	0.579165216
3	Naga	1.46E-09	0.671937205
3	Plxnb2	1.69E-09	0.734900634
3	Mid1ip1	1.83E-09	0.742115924
3	Dnajb2	2.03E-09	0.673337077
3	Sec11c	2.20E-09	0.708648037
3	Sec14l1	2.51E-09	0.69678572
3	Asrgl1	2.56E-09	0.630459767
3	Cep164	2.61E-09	0.725648998
3	Oit1	2.63E-09	0.642679315
3	Cipc	2.92E-09	0.526320936
3	Hsbp1	2.96E-09	0.625822439
3	Txnip	3.08E-09	0.816450315
3	Wdr73	3.11E-09	0.545853226
3	Nr2c2ap	3.25E-09	0.651980442
3	Cdkl2	3.41E-09	0.400949723
3	Flt3l	3.61E-09	0.429383129
3	Ropn1l	3.77E-09	0.432794086
3	Cspp1	4.39E-09	0.650704086

Table S2:Cluster specific genes identified by the differential analysis in the Organoid.

cluster	gene	p value	fold change *
3	Misp	5.42E-09	0.556008268
3	Mcee	5.51E-09	0.63968702
3	Rpap3	5.53E-09	0.60282557
3	Ift122	5.95E-09	0.563150349
3	Fgfr1op	6.31E-09	0.650410608
3	Tpd52l2	6.62E-09	0.61742398
3	Phf10	7.99E-09	0.6916081
3	Aes	8.05E-09	0.583398218
3	Tesc	8.78E-09	0.459746161
3	Gm26542	9.24E-09	0.420776896
3	Ccdc157	9.76E-09	0.456961448
3	Zdhhc1	1.01E-08	0.471728994
3	Ttc30b	1.01E-08	0.435996635
3	Baiap2l1	1.05E-08	0.525819469
3	Chchd4	1.12E-08	0.66488058
3	Ndufa12	1.13E-08	0.537187807
3	Lfng	1.13E-08	0.524697672
3	Ccno	1.28E-08	0.801045158
3	Fbxw9	1.30E-08	0.596200097
3	Gm42418	1.50E-08	0.650957869
3	Lztfl1	1.52E-08	0.664756018
3	Pddc1	1.61E-08	0.701389631
3	Vps13d	1.69E-08	0.538927206
3	Wnt7b	2.05E-08	0.637327224
3	Osbpl3	2.12E-08	0.684110222
3	Ephx4	2.24E-08	0.459271432
3	Nek2	2.35E-08	0.459148608
3	Suds3	2.42E-08	0.633819106
3	Abcb8	2.61E-08	0.624955748
3	Papola	2.88E-08	0.624382019
3	Cracr2b	3.02E-08	0.607570139
3	Bbs5	3.37E-08	0.53079794
3	Mipep	3.42E-08	0.486288537
3	Rab25	3.49E-08	0.50991237
3	Dync2li1	3.50E-08	0.657485739
3	Mien1	3.57E-08	0.633393537
3	Iqce	3.79E-08	0.49506886
3	Tagln2	4.15E-08	0.574884915
3	Pcnt	4.16E-08	0.571544161
3	Pex11g	5.12E-08	0.552935127
3	Frem2	5.13E-08	0.452973703

Table S2:Cluster specific genes identified by the differential analysis in the Organoid.

cluster	gene	p value	fold change *
3	Cep19	5.30E-08	0.564551628
3	Fam229b	5.68E-08	0.565004164
3	Dgcr6	6.31E-08	0.567325874
3	Glrx5	6.58E-08	0.5417873
3	Zc3h14	6.85E-08	0.664704147
3	Gm20661	7.01E-08	0.534240012
3	Arhgap39	8.19E-08	0.545496139
3	R3hdm4	8.23E-08	0.626323
3	Tmc4	9.38E-08	0.760216813
3	Qsox1	9.49E-08	0.643569237
3	Pfkp	1.08E-07	0.62219752
3	Rab36	1.15E-07	0.409993101
3	Slf1	1.29E-07	0.501286141
3	Bcl2l12	1.31E-07	0.542579826
3	Bok	1.37E-07	0.691656947
3	Pxk	1.41E-07	0.569791604
3	Nin	1.47E-07	0.437435076
3	Fkbp8	1.47E-07	0.548537533
3	Ube2m	1.62E-07	0.560119718
3	Epn3	1.70E-07	0.560445318
3	Med19	1.81E-07	0.561568866
3	Wfdc2	1.92E-07	0.86773041
3	Mdm2	2.00E-07	0.707737475
3	Dhx16	2.32E-07	0.466917675
3	Spata24	2.60E-07	0.469211348
3	Wdr19	2.67E-07	0.418638011
3	Ank3	2.78E-07	0.64206601
3	Heatr5a	2.90E-07	0.436071341
3	4932443I19Rik	2.94E-07	0.429138409
3	Arl2bp	3.02E-07	0.627683573
3	Fdps	3.07E-07	0.678623323
3	Nek1	3.43E-07	0.556205401
3	Nme7	3.46E-07	0.531053633
3	Psmc3ip	3.56E-07	0.523957582
3	Smc4	3.68E-07	0.618362521
3	Gnb2	3.71E-07	0.485780191
3	1500009L16Rik	3.74E-07	0.536008204
3	Slc44a4	4.09E-07	0.45762948
3	Ech1	4.57E-07	0.582867015
3	Sclt1	4.74E-07	0.612363118
3	Mul1	4.76E-07	0.544230731

Table S2:Cluster specific genes identified by the differential analysis in the Organoid.

cluster	gene	p value	fold change *
3	Dnaaf5	5.06E-07	0.468897478
3	Arl3	5.10E-07	0.570621362
3	Ric8	5.23E-07	0.535261702
3	Col27a1	5.32E-07	0.55949471
3	Gm5617	5.39E-07	0.586321276
3	Dtx3	6.95E-07	0.63064997
3	Cdc14a	7.29E-07	0.47318792
3	Mrpl39	7.32E-07	0.551042199
3	Alms1	7.65E-07	0.539426037
3	2310030G06Rik	7.67E-07	0.44075123
3	Acot7	7.74E-07	0.607284043
3	Stk11	7.83E-07	0.56603956
3	Myb	7.91E-07	0.472860187
3	Cldn7	8.07E-07	0.519895075
3	Pex7	8.17E-07	0.605512127
3	Erlin2	8.87E-07	0.435826635
3	Casz1	9.70E-07	0.498538696
3	Apbb1	9.91E-07	0.520725779
3	Cuta	1.01E-06	0.514404042
3	Pak4	1.06E-06	0.40107185
3	Adrbk2	1.07E-06	0.475271079
3	Dgat2	1.08E-06	0.436942495
3	mt-Co1	1.12E-06	0.680319151
3	Rnf181	1.13E-06	0.563493556
3	Akap9	1.16E-06	0.623601044
3	Cep89	1.20E-06	0.420700471
3	Rogdi	1.20E-06	0.496594653
3	Isoc2b	1.24E-06	0.54298924
3	Irak1bp1	1.26E-06	0.557242338
3	Gprc5c	1.26E-06	0.46192814
3	Dxo	1.30E-06	0.442289307
3	Fads3	1.32E-06	0.518267688
3	Slc9a8	1.33E-06	0.482550729
3	Galk1	1.34E-06	0.575497386
3	Wrap53	1.38E-06	0.4481079
3	Atp1a1	1.44E-06	0.550612791
3	Spata7	1.55E-06	0.433176572
3	Taf11	1.56E-06	0.560863024
3	Tmem216	1.65E-06	0.536730414
3	Six1	1.81E-06	0.54631317
3	Ccnc	1.84E-06	0.433817954

Table S2:Cluster specific genes identified by the differential analysis in the Organoid.

cluster	gene	p value	fold change *
3	Pltp	1.85E-06	0.507795925
3	H2afx	1.92E-06	0.663538948
3	1810043H04Rik	2.13E-06	0.480032782
3	Sox2	2.25E-06	0.577021196
3	Cops5	2.55E-06	0.509485658
3	Mt2	2.56E-06	0.721916288
3	Calml4	2.57E-06	0.411742129
3	Fkbpl	2.70E-06	0.400207735
3	Hyls1	2.70E-06	0.490533417
3	Aig1	2.76E-06	0.602868696
3	0610011F06Rik	3.12E-06	0.57737583
3	Kbtbd4	3.26E-06	0.402131218
3	Wbp2	3.46E-06	0.555736973
3	Ccdc61	3.59E-06	0.522308318
3	1110008L16Rik	3.60E-06	0.557863671
3	Zc3h6	3.84E-06	0.508025788
3	Eif4g3	3.90E-06	0.518958218
3	Nubpl	4.04E-06	0.484528186
3	Fam161a	4.05E-06	0.497870176
3	Ppp1r11	4.07E-06	0.502882777
3	Lamtor2	4.28E-06	0.482227533
3	Plet1	4.39E-06	0.608135707
3	Pop5	4.56E-06	0.573387512
3	Slc4a2	4.75E-06	0.557234271
3	Grhpr	4.88E-06	0.510157494
3	Suclg1	4.91E-06	0.532512733
3	Ubald2	5.19E-06	0.55798901
3	Ypel3	5.48E-06	0.5680993
3	Yipf1	5.61E-06	0.498514613
3	Blzf1	5.92E-06	0.500852941
3	Myo1d	6.30E-06	0.447959999
3	Me2	6.43E-06	0.466893749
3	Cct7	6.68E-06	0.493525028
3	Lgals4	6.80E-06	0.512460625
3	Ahcyl1	7.50E-06	0.492809696
3	Abcc1	7.97E-06	0.521604567
3	Coq10a	8.11E-06	0.457478661
3	Ccdc148	8.62E-06	0.486134608
3	Sptan1	8.92E-06	0.591336275
3	Tspan13	9.09E-06	0.45562822
3	Cars	9.30E-06	0.548539678

Table S2:Cluster specific genes identified by the differential analysis in the Organoid.

cluster	gene	p value	fold change *
3	Usp22	9.63E-06	0.507184044
3	Wdyhv1	9.67E-06	0.522521967
3	Dyrk3	1.06E-05	0.479604179
3	Cav2	1.09E-05	0.622013742
3	Cdk2ap2	1.18E-05	0.471984285
3	Hist1h2bc	1.18E-05	0.595146781
3	Epb41l4b	1.27E-05	0.408821372
3	Rita1	1.29E-05	0.428178545
3	Hgs	1.32E-05	0.527053721
3	0610009B22Rik	1.33E-05	0.534888385
3	Ptges3	1.34E-05	0.542317764
3	Atp1b1	1.39E-05	0.675998124
3	Pebp1	1.40E-05	0.443856348
3	Rassf9	1.40E-05	0.548831455
3	Abr	1.65E-05	0.460944196
3	Rrp7a	1.71E-05	0.490095352
3	Eif3c	1.76E-05	0.484628736
3	Ckmt1	1.78E-05	0.481509332
3	Plvap	1.92E-05	0.540478808
3	Wbp11	1.98E-05	0.53435809
3	Mgst2	2.01E-05	0.613485412
3	Rab11fip1	2.18E-05	0.428812293
3	Itpr2	2.19E-05	0.453846938
3	Sec23b	2.20E-05	0.50345631
3	Tmem194	2.22E-05	0.405917978
3	Etnk1	2.32E-05	0.509139061
3	Anapc11	2.32E-05	0.468284242
3	Gpx2	2.39E-05	0.642847404
3	B4galt4	2.41E-05	0.422330367
3	Cenpb	2.65E-05	0.534541468
3	Hid1	2.76E-05	0.439074165
3	Tpgs1	2.76E-05	0.435932243
3	Tefm	2.78E-05	0.408132265
3	Krcc1	2.83E-05	0.493790237
3	Zmynd8	2.93E-05	0.488549236
3	Trip4	2.93E-05	0.409077907
3	Ndufc1	2.98E-05	0.451881259
3	Ppdpf	3.17E-05	0.485614495
3	Lrp10	3.17E-05	0.517487319
3	Coasy	3.21E-05	0.459110461
3	Ssna1	3.23E-05	0.512205908

Table S2:Cluster specific genes identified by the differential analysis in the Organoid.

cluster	gene	p value	fold change *
3	Trib3	3.31E-05	0.56329054
3	Fam98c	3.33E-05	0.470468035
3	Myl12b	3.58E-05	0.537358843
3	Mrpl54	3.77E-05	0.477354675
3	Gm38037	3.86E-05	0.423963771
3	Napg	3.98E-05	0.452031357
3	Fam83h	4.03E-05	0.49823045
3	Cryz	4.11E-05	0.482482947
3	Dicer1	4.16E-05	0.514059489
3	Tmem138	4.32E-05	0.444627145
3	Rcbtb2	4.60E-05	0.481859945
3	Ndufaf7	4.62E-05	0.432222163
3	Fam102a	4.73E-05	0.42051729
3	Dmkn	4.90E-05	0.427905579
3	Csde1	4.95E-05	0.459298164
3	Plk4	5.02E-05	0.486476515
3	Mettl20	5.24E-05	0.423813247
3	Brd8	5.50E-05	0.497420377
3	Appl2	5.69E-05	0.412355751
3	Stard10	6.40E-05	0.460965404
3	Gtf2i	6.45E-05	0.508386049
3	Gas2l1	6.94E-05	0.440948399
3	Gemin7	7.17E-05	0.459961163
3	Ict1	7.39E-05	0.4709606
3	Sod1	7.86E-05	0.488638281
3	Endog	7.97E-05	0.433243627
3	Rab3ip	8.26E-05	0.492420697
3	Paf1	8.44E-05	0.419163242
3	Mif4gd	8.70E-05	0.541565166
3	Med25	9.10E-05	0.455226053
3	Fam213a	9.85E-05	0.527543593
3	Smim20	9.86E-05	0.464697938
3	Slc25a46	0.000101	0.416385462
3	Alkbh6	0.000101	0.415365863
3	Zfp704	0.000102	0.448979811
3	Hax1	0.000106	0.416630101
3	Atxn2	0.000113	0.437760273
3	Cox14	0.000117	0.405026253
3	Minos1	0.00012	0.415054102
3	BC029214	0.000122	0.432375796
3	Syne2	0.000124	0.532662989

Table S2:Cluster specific genes identified by the differential analysis in the Organoid.

cluster	gene	p value	fold change *
3	Daam1	0.000124	0.433697697
3	Tanc2	0.000124	0.581068223
3	Skiv2l	0.000127	0.461682531
3	Amdhd2	0.000131	0.417138398
3	Dynll2	0.000135	0.438022235
3	Oaz2	0.00014	0.475362704
3	Cbarp	0.000141	0.484176468
3	Fam32a	0.000141	0.495944294
3	Slc25a10	0.000146	0.425901844
3	Yif1b	0.000149	0.426223918
3	Rhoq	0.000149	0.456077841
3	Cs	0.00015	0.426848653
3	Npnt	0.000154	0.416667383
3	Pitpna	0.000157	0.426767569
3	Ppp1r35	0.000157	0.475710442
3	Haghl	0.000162	0.459495224
3	Crb3	0.000164	0.490669459
3	Nrd1	0.000168	0.436947377
3	Ildr1	0.000171	0.426394141
3	Nr2f6	0.000172	0.497513192
3	Nfe2l1	0.000177	0.449888241
3	Tmem218	0.000183	0.412867871
3	Tes	0.000187	0.423266607
3	Fam234a	0.000211	0.486713166
3	Med8	0.000214	0.432457748
3	Macrod1	0.000219	0.427349908
3	Bsdc1	0.00022	0.410538378
3	Ralgds	0.000225	0.403564857
3	Bola3	0.000225	0.446057782
3	Card19	0.000236	0.446392365
3	Ttc8	0.000243	0.446648183
3	Mvb12a	0.000243	0.453428804
3	Eps8l2	0.000255	0.431525868
3	Fzr1	0.000257	0.439564133
3	Stx18	0.000261	0.436718026
3	Pir	0.000267	0.436154939
3	Pnkp	0.000268	0.459692163
3	Babam1	0.000269	0.435035699
3	Nxt1	0.00028	0.413818024
3	Cmtm4	0.000281	0.407370715
3	Ebp	0.000282	0.485372991

Table S2:Cluster specific genes identified by the differential analysis in the Organoid.

cluster	gene	p value	fold change *
3	Tmem126b	0.000285	0.405004855
3	Gle1	0.00029	0.467193785
3	Pcm1	0.000296	0.513803856
3	Usp19	0.000301	0.418653822
3	Uhrf1bp1l	0.000319	0.439427638
3	Mrpl43	0.000325	0.426920612
3	Spop	0.00033	0.471150052
3	Lgals3bp	0.000343	0.448750041
3	Hspbp1	0.000356	0.435924608
3	Slc25a1	0.00039	0.423288599
3	Caml	0.000392	0.401394455
3	Rif1	0.000401	0.410817953
3	Sptbn1	0.000413	0.497218333
3	2510002D24Rik	0.00043	0.439717659
3	Ddx42	0.000431	0.442759644
3	Pbxip1	0.000492	0.440394173
3	Dnajb14	0.000514	0.404070128
3	Pigyl	0.000514	0.434446603
3	Slc31a1	0.000525	0.428211152
3	Lsm10	0.000561	0.435122098
3	Ubn2	0.000562	0.471320334
3	Tmem109	0.000592	0.44748964
3	Scrib	0.000592	0.459086952
3	Tmem53	0.000608	0.42608826
3	Pafah1b3	0.000647	0.461297373
3	Wwox	0.000661	0.411547497
3	Cdkn1a	0.000694	0.540651746
3	Ndufv3	0.000697	0.407511537
3	Krtcap3	0.000703	0.483382956
3	Hacd1	0.000767	0.499031553
3	Wbp1	0.000821	0.422357694
3	Sgsm3	0.000833	0.408905994
3	Mphosph8	0.000887	0.469284854
3	Coq4	0.000908	0.417531426
3	Btbd9	0.000939	0.425227249
3	Ascc1	0.001007	0.419155517
3	Adh7	0.001156	0.402091043
3	Trmt1l	0.001168	0.426529958
3	Cbr1	0.00117	0.441150913
3	Traf4	0.001222	0.433589985
3	Fabp5	0.001231	0.533172963

Table S2:Cluster specific genes identified by the differential analysis in the Organoid.

cluster	gene	p value	fold change *
3	Gak	0.001387	0.419830528
3	Rbck1	0.001422	0.412391524
3	Dnajc7	0.001505	0.437942715
3	Slc35e4	0.001622	0.404909228
3	Cttn	0.001622	0.431974532
3	Pin1	0.00201	0.40705329
3	Fasn	0.00221	0.421241318
3	Agpat4	0.002612	0.42491386
3	Hook2	0.002644	0.404938866
3	Slc39a4	0.003177	0.412498764
3	Ten1	0.003334	0.404935243
3	Irf2bpl	0.004816	0.417606662
3	Gclm	0.009502	0.412797608
4	Hmgb2	2.09E-76	1.699244725
4	Birc5	1.65E-62	1.641672639
4	Smc4	5.75E-62	1.484280452
4	Ube2c	3.79E-56	2.145619256
4	Cdca3	2.11E-55	1.292562962
4	H2afz	9.11E-53	1.172542347
4	Cenpa	2.59E-52	1.7170923
4	Ccna2	2.79E-52	1.040211181
4	Stmn1	9.62E-52	1.427301848
4	Top2a	1.90E-47	1.520530513
4	Cdc20	3.51E-47	1.466847645
4	Cks1b	8.99E-45	1.212859639
4	Cdk1	1.19E-43	1.328129099
4	Nusap1	3.94E-43	0.929336071
4	Cenpf	7.04E-41	1.209351196
4	Cenpe	1.32E-40	1.083750354
4	Pttg1	2.38E-40	1.312802817
4	Ccnb1	2.80E-39	1.073262882
4	Esco2	1.33E-38	0.745089676
4	H2afv	3.83E-38	0.957504051
4	Knstrn	6.69E-38	0.981151294
4	Arl6ip1	7.42E-38	1.1084841
4	Spc24	1.18E-36	0.912265672
4	Mki67	3.97E-34	0.677271983
4	Prc1	5.40E-34	0.802104957
4	Ect2	9.95E-34	0.802543931
4	2810417H13Rik	4.80E-33	1.073304129
4	Ccdc34	5.39E-33	1.056669241

Table S2:Cluster specific genes identified by the differential analysis in the Organoid.

cluster	gene	p value	fold change *
4	Cdca8	7.08E-32	0.91968694
4	Ccnb2	1.39E-31	0.946485335
4	Pbk	2.69E-31	0.840251451
4	Tpx2	1.40E-30	0.704853439
4	Aurkb	2.42E-30	0.564672233
4	Hmgb1	6.67E-29	0.832928414
4	Tacc3	2.04E-28	0.766270217
4	Racgap1	5.05E-28	0.842732397
4	Cdca2	2.09E-27	0.539399647
4	Hmmr	3.54E-27	0.535060762
4	Lockd	9.15E-27	0.821200604
4	Plk1	2.93E-26	0.60980922
4	Dlgap5	4.12E-25	0.514311856
4	Kifc1	6.02E-25	0.497555767
4	Mad2l1	1.45E-24	0.613423965
4	Psrc1	1.45E-24	0.510152638
4	Rrm2	1.54E-24	0.822118966
4	Cdkn3	3.17E-24	0.739250495
4	Fbxo5	7.15E-24	0.452746683
4	Tubb5	8.25E-24	0.880668197
4	Rfc4	9.74E-24	0.715262925
4	Anp32e	2.11E-23	0.809230483
4	H2afx	3.91E-23	0.891375594
4	Ckap2l	4.09E-23	0.475537269
4	Kif22	5.49E-23	0.5324948
4	Hdgf	1.13E-22	0.72726775
4	Spc25	1.48E-22	0.521015744
4	Smc2	5.57E-22	0.689956614
4	Pmf1	5.79E-22	0.761024655
4	Fam64a	6.34E-22	0.55254808
4	Gmnn	8.81E-22	0.631540842
4	Bub1	1.21E-21	0.402988057
4	Kif23	1.42E-21	0.562783304
4	Wfdc2	1.63E-21	0.560326307
4	Bub3	8.01E-21	0.80932574
4	Tmpo	1.09E-20	0.8305883
4	Ezh2	1.37E-20	0.666374801
4	Aurka	1.95E-20	0.726288904
4	Hn1	3.96E-20	0.749133567
4	Kpna2	6.95E-20	0.636332505
4	Tk1	1.79E-19	0.5913502

Table S2:Cluster specific genes identified by the differential analysis in the Organoid.

cluster	gene	p value	fold change *
4	Sapcd2	2.31E-19	0.443964898
4	Tuba1b	2.36E-19	0.741359857
4	Casc5	2.91E-19	0.522459345
4	Ckap2	5.31E-19	0.557454753
4	Cks2	5.84E-19	0.711129728
4	Cit	6.10E-19	0.519957868
4	Rad51	1.31E-18	0.478546828
4	Shcbp1	1.97E-18	0.466219459
4	Mcm7	2.55E-18	0.680175698
4	Nrm	6.97E-18	0.542738752
4	Mcm5	7.88E-18	0.562603032
4	Uhrf1	1.77E-17	0.431453255
4	Hells	1.93E-17	0.474905129
4	Gpsm2	2.11E-17	0.605665196
4	Ube2t	3.90E-17	0.436348523
4	Epcam	5.21E-17	0.65242094
4	H1f0	8.49E-17	0.879063833
4	2700094K13Rik	9.88E-17	0.735375317
4	Aspm	2.55E-16	0.457011115
4	Sgol2a	3.55E-16	0.471193553
4	Ncapg	7.78E-16	0.425473994
4	Rfc5	1.23E-15	0.567217078
4	Prim1	1.98E-15	0.477658118
4	Hmgb3	5.71E-15	0.56879187
4	Krt19	7.77E-15	0.780988682
4	Hmgn5	1.36E-14	0.543727346
4	Lsm4	1.47E-14	0.555978794
4	H1fx	1.65E-14	0.686049828
4	Spag5	6.92E-14	0.405461877
4	Terf1	7.56E-14	0.470274229
4	Cenpm	8.05E-14	0.500563859
4	Lmnb1	9.98E-14	0.476121593
4	Krtcap2	1.08E-13	0.606182508
4	Nucks1	1.44E-13	0.647200476
4	Dnajc9	1.65E-13	0.627394732
4	Lig1	2.14E-13	0.596810826
4	Vrk1	2.99E-13	0.581853356
4	Krt8	6.30E-13	0.580620051
4	Nup37	9.67E-13	0.517286683
4	Rrm1	1.28E-12	0.565198753
4	Sap30	2.69E-12	0.697641474

Table S2:Cluster specific genes identified by the differential analysis in the Organoid.

cluster	gene	p value	fold change *
4	Cldn3	2.85E-12	0.41933633
4	Mis18a	2.86E-12	0.423113963
4	Ccdc18	3.74E-12	0.411524881
4	Cldn7	3.97E-12	0.715331598
4	Hmgn2	6.60E-12	0.411527405
4	Reep6	1.06E-11	0.43603883
4	Rangap1	2.42E-11	0.437507509
4	Actl6a	5.61E-11	0.580875282
4	Trib3	6.92E-11	0.516903483
4	Lsm2	8.49E-11	0.573707647
4	Rad21	1.34E-10	0.504004988
4	Ubald2	1.54E-10	0.562865272
4	Srsf6	1.65E-10	0.534373209
4	Cdk4	1.96E-10	0.461786661
4	Parp2	2.49E-10	0.450319511
4	Rnaseh2c	3.12E-10	0.476536234
4	Dazap1	3.98E-10	0.491409829
4	Mcm3	4.50E-10	0.459699736
4	Reep4	5.41E-10	0.469055836
4	Serbp1	7.85E-10	0.427905229
4	Dbf4	9.41E-10	0.403237003
4	Set	1.02E-09	0.497447173
4	Arpc5l	1.05E-09	0.504898872
4	Usp1	1.19E-09	0.41814722
4	Aspa	1.54E-09	0.556612843
4	Cbx1	1.83E-09	0.504729317
4	Ewsr1	2.98E-09	0.41190292
4	Dek	3.05E-09	0.495684889
4	Ncaph2	3.51E-09	0.493220371
4	Msln	8.31E-09	0.801570079
4	Tubb4b	8.69E-09	0.540969378
4	Gltp	9.97E-09	0.497241395
4	Ddx39b	1.28E-08	0.469279398
4	Sae1	1.44E-08	0.48347756
4	Hnrnpa3	1.66E-08	0.499183106
4	Sf3b4	1.76E-08	0.46778374
4	Lsm5	2.12E-08	0.431917464
4	Ilf2	2.26E-08	0.484909557
4	Hnrnpf	2.58E-08	0.468105583
4	Apobec3	2.69E-08	0.524774036
4	Mrpl24	2.73E-08	0.437192994

Table S2:Cluster specific genes identified by the differential analysis in the Organoid.

cluster	gene	p value	fold change *
4	Slirp	3.85E-08	0.442164327
4	Srsf1	3.91E-08	0.420153869
4	Hnrnpa2b1	3.92E-08	0.429073299
4	Trim28	4.39E-08	0.543605952
4	Rfc2	4.84E-08	0.428180082
4	Srsf3	6.25E-08	0.406717648
4	Trim2	7.30E-08	0.486836006
4	Ckap5	7.55E-08	0.400618847
4	Larp7	8.42E-08	0.450065345
4	Crabp2	9.19E-08	0.466057947
4	Pnn	9.88E-08	0.417284896
4	Asf1a	1.18E-07	0.407121823
4	Rbm8a	1.85E-07	0.414288162
4	Exosc8	2.02E-07	0.42842817
4	Krt18	2.03E-07	0.429847807
4	Ssrp1	2.15E-07	0.423177878
4	Prpf40a	2.19E-07	0.415243073
4	Taf15	2.60E-07	0.427822443
4	Atf5	2.65E-07	0.42302881
4	Rpf1	2.66E-07	0.412258556
4	Phgdh	3.04E-07	0.463619671
4	Dctpp1	3.40E-07	0.452639479
4	Sptssb	3.40E-07	0.493972756
4	Sypl	4.14E-07	0.493791752
4	Dut	4.21E-07	0.437339746
4	Alyref	4.67E-07	0.405100164
4	Ngdn	4.76E-07	0.418391235
4	Ranbp1	4.87E-07	0.446774765
4	Anxa3	5.52E-07	0.537326836
4	Krcc1	5.61E-07	0.401785359
4	Cebpg	7.82E-07	0.419391864
4	Eif2s1	1.02E-06	0.456357007
4	Tyms	1.13E-06	0.42963662
4	Lsm8	1.30E-06	0.427493331
4	Gipc1	1.46E-06	0.424072031
4	Nop56	1.63E-06	0.417032726
4	Tspan8	2.06E-06	0.449022736
4	Pcna	3.16E-06	0.416637388
4	Psmd14	5.15E-06	0.404940768
4	Sptssa	5.19E-06	0.427337688
4	Papola	5.39E-06	0.406416533

Table S2:Cluster specific genes identified by the differential analysis in the Organoid.

cluster	gene	p value	fold change *
4	Smc6	1.15E-05	0.429235754
4	Hat1	1.61E-05	0.404263578
4	Cldn6	2.60E-05	0.433237121
5	Ly6d	0	1.823742329
5	Gsta4	0	1.799718775
5	Wfdc2	0	1.407448719
5	Epcam	5.52E-306	1.495413613
5	Spint2	2.06E-303	1.29957543
5	Krt7	5.44E-274	1.406820162
5	Fxyd3	2.91E-253	1.2022928
5	Krt19	3.33E-246	1.6637729
5	Eif3f	1.19E-237	0.99878454
5	Cyp2f2	4.94E-235	1.636193459
5	Gsto1	5.89E-234	1.099143993
5	Sfn	1.13E-224	1.372514743
5	Clu	5.33E-220	1.688248324
5	Klf5	8.95E-218	1.292656809
5	Avpi1	8.50E-197	1.33007236
5	S100a14	8.40E-184	1.090024812
5	Rab25	8.54E-181	1.113232469
5	Krt8	1.13E-180	1.067120122
5	Bcam	5.56E-180	1.044792553
5	Perp	1.05E-176	0.862367161
5	Cd24a	4.98E-174	1.064994348
5	Cldn7	2.29E-172	1.139356004
5	Sprr1a	4.79E-170	1.924404217
5	Fam3c	5.53E-169	1.030392425
5	Foxq1	4.84E-165	1.202565787
5	S100a16	1.39E-163	1.002635772
5	Ezr	3.43E-162	0.811223963
5	Anxa8	1.00E-158	1.068981423
5	Gpx2	1.66E-154	1.075093028
5	Pthlh	5.96E-153	1.675147473
5	Lmo7	2.51E-150	1.16236807
5	Cd9	1.75E-146	0.893497625
5	Ahr	1.14E-140	1.040423783
5	Areg	4.11E-140	1.441688972
5	Krt18	5.03E-140	1.023428241
5	Ces1d	7.77E-139	1.377788507
5	Ywhaq	1.34E-138	0.769488738
5	Aqp5	8.63E-134	1.096581051

Table S2:Cluster specific genes identified by the differential analysis in the Organoid.

cluster	gene	p value	fold change *
5	Krt14	2.19E-133	0.762988309
5	Col25a1	2.78E-129	0.974606673
5	Dnajb4	1.50E-128	1.049293589
5	Tacstd2	8.11E-127	0.894595737
5	Cldn4	3.17E-125	1.187242058
5	Cdkn1a	5.52E-120	1.066194182
5	Hes6	2.63E-118	1.073897135
5	Sprr2a3	3.02E-117	1.59824558
5	Krt15	1.09E-116	1.603205892
5	Anxa1	3.65E-115	0.798617909
5	Msln	9.95E-115	1.449103797
5	2200002D01Rik	3.64E-114	0.789004614
5	Cdh13	2.18E-113	0.796460936
5	Ccnd2	6.57E-113	0.683789951
5	Fam107b	2.01E-112	0.899799481
5	Tmc4	3.54E-110	0.775377894
5	Shroom3	2.99E-108	0.973780211
5	Gprc5a	1.48E-106	0.873727387
5	Tspan1	2.37E-105	0.705290038
5	Jup	1.76E-102	0.807700488
5	Ppp2r2b	1.12E-100	0.765641105
5	Ckmt1	3.06E-100	0.731857249
5	Adam28	2.45E-99	0.734593886
5	Ifrd1	1.09E-97	0.960610004
5	Hspa4l	1.86E-97	0.836645834
5	F3	1.30E-93	0.950336658
5	Lsr	1.76E-93	0.624576766
5	Aspa	1.09E-90	0.743207326
5	Atp1b1	3.83E-90	0.784706966
5	Fbxo2	7.18E-90	0.747419244
5	Tspan8	2.18E-87	0.712289665
5	Eya2	6.78E-87	0.631733398
5	Homer2	2.66E-86	0.621848439
5	Pdzk1ip1	2.76E-84	0.803179974
5	Adh7	4.29E-84	0.850832014
5	Aqp4	1.01E-83	0.704061924
5	Plaur	1.05E-83	0.925931629
5	Kcnk1	8.85E-83	0.631167608
5	Krt6a	1.43E-82	1.179052619
5	Noct	6.89E-82	0.796200429
5	Trim2	3.27E-81	0.972738499

Table S2:Cluster specific genes identified by the differential analysis in the Organoid.

cluster	gene	p value	fold change *
5	Urah	9.59E-81	0.535647957
5	Gstm1	1.44E-79	0.905144709
5	Ifitm1	2.63E-79	0.617802304
5	Socs2	1.04E-78	0.739816349
5	Taldo1	1.48E-78	0.643827317
5	Pax9	3.67E-78	0.638048297
5	Mapk13	2.56E-77	0.646654469
5	Mgst2	2.36E-76	0.620835376
5	Fam110c	1.56E-74	0.773549283
5	Tpd52	4.31E-74	0.647999536
5	Cdh1	9.93E-74	0.58127111
5	Ly6e	2.71E-73	0.600721478
5	Sox2	8.69E-73	0.570803499
5	Elf3	2.66E-70	0.649554368
5	Aig1	3.66E-70	0.622694527
5	H2afj	1.39E-69	0.517490833
5	Sox11	8.96E-69	0.694528798
5	Krtcap3	2.24E-68	0.524221183
5	Pmm1	3.48E-68	0.601376693
5	Tsc22d1	1.36E-67	0.590012629
5	Fermt1	3.75E-67	0.607104597
5	Trappc6a	1.65E-66	0.579276252
5	Ube2d3	2.58E-66	0.462726859
5	Nr4a1	3.43E-66	0.582892282
5	Gclm	5.88E-66	0.670770257
5	Tmem238	7.81E-66	0.597482297
5	Ltbp4	1.16E-65	0.63008075
5	Sypl	1.32E-65	0.590418761
5	Klf13	1.38E-65	0.668064407
5	Zfand6	2.53E-65	0.62477615
5	Arl4a	2.76E-65	0.752022485
5	Fgfbp1	1.26E-64	0.669872668
5	Sox21	2.56E-64	0.537840195
5	Syne4	3.47E-64	0.485808201
5	Gltscr2	4.95E-64	0.553881575
5	Serpinb5	9.60E-64	0.603707865
5	Ripk4	2.44E-63	0.4344643
5	Slc26a2	2.62E-61	0.614182987
5	Tst	2.24E-60	0.838183829
5	Lgals3	2.44E-60	0.460175307
5	Rps29	4.79E-60	0.439224226

Table S2:Cluster specific genes identified by the differential analysis in the Organoid.

cluster	gene	p value	fold change *
5	Foxg1	1.25E-59	0.54418898
5	Nudt14	1.63E-59	0.560800987
5	Hspa1a	1.69E-59	0.566004702
5	Tspan15	2.23E-59	0.516002989
5	Adrb2	2.81E-59	0.578373688
5	Alcam	3.09E-59	0.56401702
5	Ehf	4.92E-59	0.456617423
5	Mdh1	7.12E-59	0.479313179
5	Ccnl1	1.76E-58	0.542134187
5	Cystm1	1.83E-58	0.587334605
5	Pitx1	2.10E-58	0.455578745
5	Aldh3a1	6.18E-58	0.646308231
5	Cbr2	8.83E-58	0.679583679
5	Itgb4	1.15E-57	0.446384943
5	Emp1	1.18E-57	0.637506789
5	Rpl36al	2.49E-57	0.444724838
5	Sftpd	2.67E-57	0.821126351
5	Rpl22l1	3.23E-57	0.654662092
5	Klc3	1.01E-56	0.472485288
5	Baz1a	3.25E-56	0.582798003
5	Eif5	3.66E-56	0.505854904
5	Msmo1	3.17E-55	0.675978131
5	Tra2a	7.47E-54	0.519841171
5	Plek2	1.44E-53	0.513315451
5	Onecut2	2.10E-52	0.419023664
5	Siva1	2.47E-52	0.569447979
5	Tprg	5.15E-52	0.458403609
5	Vill	5.53E-52	0.405087216
5	Echs1	7.98E-52	0.516713908
5	Hbegf	3.96E-51	0.708893827
5	Cisd1	1.16E-50	0.455400569
5	Nr4a2	1.20E-50	0.677983527
5	4930523C07Rik	1.23E-50	0.482270676
5	Srsf5	1.28E-50	0.498198786
5	Tkt	3.30E-50	0.472776895
5	Skil	1.28E-49	0.570850302
5	Cldn3	1.89E-49	0.432574639
5	Neat1	2.44E-49	0.545788157
5	Dusp1	8.33E-49	0.744616758
5	Ttc9	1.11E-48	0.501259888
5	Crb3	2.36E-48	0.403532352

Table S2:Cluster specific genes identified by the differential analysis in the Organoid.

cluster	gene	p value	fold change *
5	Dedd2	3.63E-48	0.447072153
5	Crabp2	5.90E-48	0.511949063
5	Fkbp4	2.58E-47	0.443096846
5	Vamp8	9.59E-47	0.411950435
5	Slc39a4	1.16E-46	0.403338501
5	Sdc1	1.22E-46	0.471129762
5	Serpinb11	1.63E-46	0.496243971
5	Rac3	2.58E-46	0.452807007
5	Sf3b6	4.87E-46	0.457461267
5	Anxa3	7.17E-46	0.559426601
5	Epas1	9.63E-46	0.452833005
5	Bhlhe40	1.29E-45	0.562917278
5	Gpr87	1.35E-45	0.521678511
5	Bace2	1.82E-45	0.4692982
5	Osbpl3	3.78E-45	0.465178923
5	Dcxr	9.06E-45	0.672287057
5	Kcnq1ot1	4.74E-44	0.536988101
5	Wnt4	1.40E-43	0.413804289
5	Krt20	1.99E-43	0.618807541
5	Basp1	1.11E-42	0.499327344
5	Ahsa1	1.47E-42	0.463698614
5	Id2	3.57E-42	0.556134007
5	Krt23	4.85E-42	0.615104861
5	Cirbp	9.52E-42	0.47314332
5	Atp6v1d	5.86E-41	0.453453062
5	Ffar4	7.41E-41	0.480120726
5	Krt17	1.38E-40	0.89130566
5	Stard10	1.79E-40	0.417009424
5	Ythdc1	2.70E-40	0.497619451
5	Taf10	1.28E-39	0.422822438
5	Ptprf	1.58E-39	0.445409529
5	Crip2	1.97E-39	0.404957852
5	Tubb4b	5.20E-39	0.424714072
5	Cav2	7.15E-39	0.466475616
5	Tgif1	7.62E-39	0.517843747
5	Fuca2	2.35E-38	0.414862067
5	Klf3	1.29E-37	0.489393873
5	Larp1b	3.84E-37	0.409555219
5	Ces1f	5.51E-37	0.664659383
5	Zfp36l1	7.78E-37	0.448269892
5	Sh3yl1	1.17E-36	0.408547222

Table S2:Cluster specific genes identified by the differential analysis in the Organoid.

cluster	gene	p value	fold change *
5	Pgap2	1.27E-36	0.414988997
5	Grasp	1.55E-36	0.473683042
5	Pmaip1	2.06E-36	0.441415136
5	Ttc14	6.91E-36	0.437815966
5	Rab10	1.31E-35	0.413027455
5	Pnrc1	1.67E-35	0.437132969
5	Lypd2	1.71E-35	0.460247181
5	Limk2	5.24E-35	0.412945914
5	Rbm25	8.15E-35	0.416736317
5	Egln3	9.24E-35	0.46293
5	Isg20	2.50E-34	0.41866963
5	Hras	4.87E-34	0.426117724
5	2010111I01Rik	3.14E-33	0.453622238
5	Cmip	4.23E-33	0.431550406
5	Lmo4	4.41E-33	0.409316773
5	Atf3	8.73E-33	0.574906675
5	Tnfaip8	8.79E-33	0.496419666
5	Cd14	3.33E-32	0.437825816
5	Srxn1	7.18E-31	0.505193637
5	Lurap1l	2.06E-30	0.458032088
5	Tuba4a	3.08E-30	0.443768815
5	Gpd2	5.90E-30	0.400374097
5	Alkbh1	7.14E-30	0.416258748
5	Bpgm	2.55E-28	0.48403594
5	Fam213a	1.42E-27	0.413876048
5	Sema3c	2.04E-27	0.402883861
5	Btg1	6.87E-27	0.409404998
5	Ltf	1.16E-26	1.127846318
5	8430408G22Rik	3.08E-24	0.400525108
5	Klf6	8.51E-24	0.453172451
5	Dnaja4	8.89E-23	0.412636914
5	Reg3g	5.85E-17	0.816313433
5	Nppc	1.42E-14	0.435486781
5	Hp	6.72E-11	0.54110486
5	Lcn2	1.22E-06	0.40051354
6	Krt5	1.73E-160	2.70169159
6	Hspb1	4.03E-102	2.352654542
6	Krt14	1.16E-80	1.861942819
6	Ecm1	4.03E-57	1.283212506
6	Hspb8	1.45E-48	1.193185125
6	Sfn	1.51E-46	1.208900876

Table S2:Cluster specific genes identified by the differential analysis in the Organoid.

cluster	gene	p value	fold change *
6	Fxyd3	2.03E-46	1.188783905
6	Lmo1	2.66E-42	1.0482416
6	Ucp2	5.66E-41	0.969347328
6	Perp	1.76E-39	1.060742747
6	Urah	8.92E-39	1.022160071
6	Krt17	1.28E-34	1.538286299
6	Ociad2	6.75E-34	0.861583524
6	Mt2	2.95E-33	1.179799001
6	Hcar2	6.92E-33	1.14246861
6	S100a10	1.36E-32	0.86248736
6	Glul	1.84E-32	1.004749956
6	Palmd	1.19E-30	0.662876847
6	Cat	4.19E-30	0.770632009
6	Mt1	6.90E-30	1.010100191
6	Vsnl1	1.34E-29	0.748300885
6	Pycard	5.09E-28	0.641749445
6	Rab27b	4.61E-27	0.764431584
6	Ly6a	4.72E-27	1.256282604
6	Dusp6	7.15E-27	0.944542107
6	App	7.00E-25	0.735958439
6	Lgals3	1.32E-23	0.970603435
6	Ifitm10	1.46E-23	0.560275709
6	Anxa3	9.37E-23	0.774080625
6	Pitx1	2.84E-22	0.777985542
6	Phlda3	1.30E-21	0.654972738
6	Hbegf	2.88E-20	0.814119199
6	Serpinb5	3.78E-20	0.780318302
6	Sostdc1	4.49E-20	0.531542218
6	Anxa1	1.18E-19	0.726204859
6	Gpx2	1.87E-19	0.842846027
6	Anxa8	2.13E-19	0.64698534
6	Slc25a5	5.22E-19	0.547523139
6	Ramp3	1.10E-18	0.870583271
6	Upk3bl	4.68E-18	0.824493095
6	Krt7	2.84E-17	0.609909275
6	Stra6	7.44E-17	0.412823249
6	4930523C07Rik	7.98E-17	0.76489862
6	Lypd3	1.36E-16	0.598813942
6	Ppp2r2c	1.71E-16	0.516668251
6	Tacstd2	2.44E-16	0.717621174
6	Atf3	4.35E-16	0.805879665

Table S2:Cluster specific genes identified by the differential analysis in the Organoid.

cluster	gene	p value	fold change *
6	Kif21a	4.43E-16	0.600197857
6	Lad1	8.32E-16	0.529739919
6	F11r	8.92E-16	0.561874315
6	Ctsc	1.36E-15	0.642391313
6	Tuba4a	1.82E-15	0.605311681
6	Ackr3	3.25E-15	0.679141426
6	Ube2f	5.02E-15	0.444797805
6	Pdlim1	7.40E-15	0.549094039
6	Golim4	1.02E-14	0.511986894
6	Yrdc	1.17E-14	0.473819644
6	Moxd1	1.30E-14	0.404377203
6	Serpinb10	1.67E-14	0.535691601
6	Plk2	2.10E-14	0.574277784
6	Arrdc4	2.52E-14	0.522173748
6	Vps37b	4.14E-14	0.531471042
6	Arhgdib	4.81E-14	0.556256228
6	Krt6a	5.02E-14	0.834887315
6	Gsr	8.63E-14	0.516382833
6	Ly6d	2.07E-13	0.449578828
6	4833423E24Rik	2.80E-13	0.429423241
6	Lrrfip1	3.93E-13	0.519202158
6	Col17a1	4.03E-13	0.579740033
6	Vwa1	4.60E-13	0.412993852
6	Dsp	5.53E-13	0.479454787
6	Lurap1l	1.10E-12	0.608959793
6	Trim29	1.59E-12	0.57623139
6	S100a14	1.68E-12	0.595859093
6	Lgals7	2.38E-12	0.649004239
6	Sdc1	7.19E-12	0.450036667
6	Avpi1	7.43E-12	0.406648268
6	Klf4	1.54E-11	0.565079882
6	Runx1	1.61E-11	0.415458682
6	Bcl11b	2.00E-11	0.468412659
6	Krt13	2.28E-11	1.003836193
6	Arl13b	2.34E-11	0.476262798
6	Itga6	2.50E-11	0.55962991
6	Areg	3.25E-11	0.661198968
6	Icam1	5.52E-11	0.534631556
6	Gabrp	8.14E-11	0.589977198
6	Ly6e	8.61E-11	0.507594294
6	Arl4c	1.37E-10	0.541357766

Table S2:Cluster specific genes identified by the differential analysis in the Organoid.

cluster	gene	p value	fold change *
6	Flrt3	1.48E-10	0.466178683
6	Cnot6l	1.51E-10	0.475989263
6	Ifitm1	1.75E-10	0.719885128
6	Wnt10a	1.86E-10	0.454621744
6	Cyp4b1	2.32E-10	0.410247258
6	Irx5	2.75E-10	0.43702258
6	Cpne8	3.25E-10	0.402605298
6	Coq10b	3.98E-10	0.467785657
6	Ets2	4.34E-10	0.427612991
6	Rras2	6.77E-10	0.403817736
6	Dnaja1	7.38E-10	0.471449538
6	Gpr87	1.29E-09	0.51605069
6	Pltp	2.13E-09	0.428198535
6	Osr2	2.22E-09	0.47861804
6	Emp2	2.41E-09	0.408645029
6	Tns4	2.67E-09	0.430436239
6	Ehf	6.37E-09	0.460849163
6	Gltscr2	6.76E-09	0.412091472
6	Pthlh	8.32E-09	0.525894148
6	Serinc2	1.25E-08	0.411984368
6	Klf5	1.73E-08	0.439197594
6	Ifi202b	1.79E-08	0.462310932
6	Trp63	2.47E-08	0.483953156
6	Cav2	3.90E-08	0.405539251
6	Id4	3.91E-08	0.406099324
6	Emp1	4.44E-08	0.450827154
6	Acsl3	5.77E-08	0.410278803
6	Tnfaip8	9.24E-08	0.433261253
6	Cbr2	1.62E-07	0.500896843
6	Nrip1	3.84E-07	0.408480517
6	Lamc2	4.30E-07	0.404450092
6	Dusp14	7.15E-07	0.427487973
6	Gsto1	1.09E-06	0.693475535
6	Ltf	1.39E-06	0.659725534
6	Sgk1	1.69E-06	0.517439731
6	Prss23	3.00E-06	0.407644186
6	Plek2	5.44E-06	0.403550912
6	Fst	8.80E-06	0.655697395
6	Fgfbp1	5.69E-05	0.578848018
6	Krt16	0.002544	0.477537959
6	Cldn4	0.006403	0.455986765

Table S3. Differentially expressed genes between Lgr5_1 and Lgr5_2

gene	fold change *	p value	Adjusted p value
Lgals1	-3.933507993	1.19E-23	1.80E-19
Sparc	-3.311605515	8.50E-17	1.28E-12
Dcn	-3.145369214	2.72E-11	4.10E-07
Col3a1	-3.115959694	6.31E-14	9.50E-10
Ctla2a	-2.703667543	9.40E-09	1.42E-04
Bgn	-2.696418013	1.89E-16	2.84E-12
Col1a1	-2.644233003	7.39E-15	1.11E-10
Igfbp3	-2.485017539	1.38E-07	2.08E-03
Serpinh1	-2.462043746	1.22E-11	1.84E-07
Serpinf1	-2.449164351	2.55E-13	3.84E-09
Col1a2	-2.383334282	1.51E-13	2.28E-09
Igfbp7	-2.33686282	6.08E-09	9.17E-05
Vim	-2.296674529	9.34E-10	1.41E-05
Timp3	-2.204728746	1.41E-08	2.13E-04
Sfrp1	-2.159555637	9.94E-08	1.50E-03
Klf2	-2.127980984	1.24E-07	1.86E-03
Col5a2	-2.085115373	2.86E-11	4.31E-07
Id3	-2.044335654	1.21E-08	1.82E-04
Rcn3	-1.996299999	1.23E-11	1.85E-07
Aebp1	-1.937583694	9.14E-11	1.38E-06
B2m	-1.863180825	5.85E-16	8.82E-12
Timp1	-1.859544689	6.56E-10	9.89E-06
Igfbp4	-1.774322692	1.15E-07	1.73E-03
Wbp5	-1.744268061	2.90E-11	4.37E-07
Cpxm1	-1.731016617	1.54E-05	2.31E-01
Selm	-1.661642553	4.60E-10	6.92E-06
Tceal8	-1.63665571	2.04E-09	3.07E-05
Cxcl12	-1.629281246	1.45E-06	2.18E-02
Cald1	-1.622908226	1.51E-11	2.27E-07
Sdc2	-1.603558565	3.77E-07	5.68E-03
Ccl7	-1.556483232	4.76E-06	7.17E-02
Ccdc80	-1.553517777	1.44E-05	2.17E-01
Postn	-1.51433019	9.31E-08	1.40E-03
Pcolce	-1.509460638	1.14E-07	1.72E-03
Cdkn2a	-1.501499782	4.63E-09	6.98E-05
Rasl11a	-1.459399716	2.86E-05	4.31E-01
Nov	-1.45069933	1.67E-06	2.51E-02
Gng11	-1.445587687	5.10E-09	7.68E-05
Fbln2	-1.436744772	5.46E-08	8.22E-04
Nsg1	-1.430892805	1.14E-08	1.71E-04
Mif	-1.39557716	1.25E-07	1.88E-03

Table S3. Differentially expressed genes between Lgr5_1 and Lgr5_2

gene	fold change *	p value	Adjusted p value
Myl9	-1.386630671	2.50E-05	3.76E-01
Plac8	-1.354033347	1.22E-07	1.84E-03
Cst3	-1.350747955	3.68E-07	5.54E-03
Grem1	-1.327497424	6.40E-08	9.64E-04
Efemp2	-1.314370574	2.97E-08	4.48E-04
Ngfrap1	-1.291234465	2.18E-08	3.28E-04
Lrp1	-1.260921713	3.90E-08	5.88E-04
Loxl1	-1.255623869	4.97E-11	7.49E-07
Tmsb10	-1.244032611	3.70E-07	5.57E-03
Cmtm3	-1.232241829	4.37E-10	6.59E-06
Col6a1	-1.227051286	2.83E-07	4.26E-03
Mxra8	-1.220722439	3.49E-06	5.25E-02
Lhfp	-1.199633293	1.79E-07	2.70E-03
Pdlim2	-1.196746414	3.18E-05	4.79E-01
Timp2	-1.191294124	1.44E-06	2.16E-02
Fam162a	-1.189048038	2.13E-06	3.21E-02
Cd302	-1.178399798	6.72E-06	1.01E-01
Tubb6	-1.172764921	1.22E-05	1.83E-01
Col12a1	-1.166954559	1.16E-06	1.74E-02
Emp3	-1.134346907	6.47E-06	9.75E-02
Csf1	-1.133642051	2.12E-06	3.20E-02
Nudt4	-1.09960902	1.25E-07	1.88E-03
Eva1b	-1.086625163	1.26E-06	1.90E-02
Ctsz	-1.077181056	5.26E-07	7.93E-03
Cdc42ep3	-1.077014363	3.92E-06	5.90E-02
Map1lc3a	-1.071689564	2.28E-06	3.44E-02
Lbh	-1.054211443	1.49E-06	2.24E-02
Prrx1	-1.013627606	5.10E-09	7.68E-05
Pde1a	-0.989365337	5.10E-09	7.68E-05
Wisp1	-0.977155312	4.15E-08	6.25E-04
Has2	-0.972452553	8.74E-06	1.32E-01
Mfap2	-0.964101319	2.19E-06	3.29E-02
Fstl1	-0.943913823	1.22E-07	1.84E-03
Nr2f1	-0.942654875	1.67E-06	2.52E-02
Calu	-0.931663582	5.34E-06	8.04E-02
Pdgfrb	-0.9271987	1.67E-06	2.52E-02
Fbln1	-0.920710523	5.14E-06	7.75E-02
Fhl2	-0.915380446	2.94E-05	4.42E-01
Olfml3	-0.90752279	1.67E-06	2.52E-02
Slc7a2	-0.86789767	6.43E-06	9.69E-02
Lgi2	-0.867138283	8.95E-06	1.35E-01

Table S3. Differentially expressed genes between Lgr5_1 and Lgr5_2

gene	fold change *	p value	Adjusted p value
Ptgs1	-0.859029386	1.21E-05	1.82E-01
Plod2	-0.836713919	1.66E-06	2.50E-02
Pdlim4	-0.81599942	8.74E-06	1.32E-01
Cdh11	-0.807624682	4.15E-08	6.25E-04
Ctsk	-0.800363412	8.74E-06	1.32E-01
Dpysl3	-0.791210488	5.77E-06	8.69E-02
Psmd5	-0.744240167	1.50E-07	2.26E-03
Phldb2	-0.730963361	8.74E-06	1.32E-01
S100a1	-0.724680758	1.29E-05	1.95E-01
Rab34	-0.711155006	3.10E-05	4.68E-01
Lama4	-0.708976763	1.67E-06	2.52E-02
Plpp3	-0.683348996	8.74E-06	1.32E-01
Myeov2	-0.682177996	2.53E-05	3.82E-01
Kdelr3	-0.679283329	6.57E-06	9.90E-02
Tomm5	-0.675096251	2.61E-05	3.93E-01
Scarf2	-0.63974762	8.74E-06	1.32E-01
Map4	-0.622943559	1.42E-05	2.14E-01
Qpct	-0.60556909	8.74E-06	1.32E-01
Gem	-0.524298072	6.44E-06	9.71E-02
Slc30a5	-0.420916332	1.04E-05	1.57E-01
Psme1	0.843882334	1.37E-05	2.07E-01
Tuba4a	0.948936605	1.54E-05	2.32E-01
Gltscr2	0.968232152	2.78E-05	4.18E-01
Cirbp	0.995095114	6.35E-06	9.56E-02
Aig1	1.033823533	3.10E-06	4.67E-02
H2-D1	1.095027446	3.82E-07	5.76E-03
Pitx1	1.109255302	1.61E-05	2.42E-01
Ezr	1.13588339	2.60E-05	3.92E-01
Anxa1	1.1586122	1.38E-06	2.07E-02
Tmc4	1.167227289	5.47E-06	8.24E-02
Eif3f	1.204483522	1.93E-08	2.91E-04
Ly6e	1.318418726	2.52E-06	3.80E-02
Krt8	1.378755843	1.15E-06	1.73E-02
Krt18	1.382895674	4.20E-07	6.33E-03
Anxa8	1.394677198	9.45E-07	1.42E-02
Lmo7	1.397189572	1.08E-05	1.63E-01
Tacstd2	1.403784564	3.49E-08	5.26E-04
Trim2	1.420821066	4.52E-06	6.81E-02
Perp	1.425856709	3.26E-08	4.91E-04
Rab25	1.44441173	8.96E-06	1.35E-01
Hes6	1.513525565	1.09E-05	1.64E-01

Table S3. Differentially expressed genes between Lgr5_1 and Lgr5_2

gene	fold change *	p value	Adjusted p value
S100a16	1.547725061	3.59E-07	5.41E-03
Cd24a	1.631796389	1.87E-07	2.82E-03
Avpi1	1.728311437	5.73E-08	8.63E-04
Sprr1a	1.763943958	1.93E-05	2.91E-01
Gsto1	1.795945872	7.93E-07	1.20E-02
S100a14	1.833403534	6.10E-10	9.19E-06
Spint2	2.001677383	1.74E-12	2.62E-08
Klf5	2.031702639	1.65E-11	2.48E-07
Gpx2	2.05373539	7.96E-09	1.20E-04
Krt19	2.093457581	1.01E-07	1.53E-03
Clu	2.111669841	1.58E-06	2.38E-02
Krt7	2.206029512	2.46E-09	3.71E-05
Cyp2f2	2.304594388	2.07E-10	3.13E-06
Epcam	2.324802668	1.39E-10	2.10E-06
Gsta4	2.445766921	1.49E-13	2.24E-09
Wfdc2	2.562240003	2.60E-16	3.92E-12
Fxyd3	2.600897447	4.96E-11	7.48E-07
Sfn	2.771746625	8.38E-09	1.26E-04
Krt14	2.877080886	3.45E-09	5.20E-05
Ly6d	2.998619786	2.66E-15	4.00E-11

* fold change for differentially expressed genes between epithelial cluster and fibroblast cluster is na

Table S4. The differentially expression of Wnt related genes between epithelial and fibroblas

gene	p value	Adjusted p value	fold change *
Col1a1	0	0	-2.417279479
Sfrp1	5.93E-216	8.9335E-212	-2.050607432
Grem1	3.80E-209	5.7247E-205	-1.326195833
Wisp1	2.04E-206	3.0733E-202	-1.034760698
Lrp1	2.59E-178	3.9018E-174	-1.080624682
Krt6a	1.70E-141	2.5611E-137	1.734564734
Rspo3	5.12E-128	7.7133E-124	-0.854205222
Bicc1	2.70E-119	4.0676E-115	-0.627935942
Sox2	7.05E-115	1.0621E-110	0.730680136
Cdh1	1.25E-105	1.8831E-101	0.685627869
Fermt2	5.20E-105	7.8338E-101	-0.670145046
Tgfb1i1	3.05E-91	4.59483E-87	-0.406135268
Barx1	1.09E-88	1.64209E-84	-0.494256723
Nkd1	1.89E-82	2.84729E-78	-0.403924455
Zeb2	4.56E-80	6.86964E-76	-0.386152116
Plpp3	1.05E-79	1.58183E-75	-0.577786008
Isl1	2.45E-79	3.69093E-75	-0.442622614
Wnt4	4.73E-71	7.12575E-67	0.503058554
Sdc1	4.88E-67	7.35172E-63	0.650822984
Egr1	5.49E-63	8.27069E-59	-0.623132231
Sulf1	3.33E-62	5.01665E-58	-0.388753969
Dkk3	4.93E-61	7.42705E-57	-0.390418704
Celsr2	2.84E-56	4.27846E-52	0.407509855
Hic1	1.28E-53	1.92832E-49	-0.280492456
Wnt7b	2.93E-51	4.41405E-47	0.353789992
Cd44	5.02E-48	7.56263E-44	-0.44968687
Slc9a3r1	2.92E-47	4.39898E-43	0.509971116
Dab2	8.79E-46	1.32421E-41	-0.242275728
Dkk2	1.92E-44	2.89248E-40	-0.363645129
Ddb1	5.12E-44	7.71328E-40	-0.473092731
Ryk	5.40E-43	8.1351E-39	-0.40274682
Apoe	2.09E-42	3.14859E-38	-0.845713006
Cdh2	3.67E-42	5.52886E-38	-0.198896953
Celsr1	4.36E-41	6.56834E-37	0.257165445
Hmga2	5.16E-41	7.77354E-37	-0.436220037
Nfatc4	7.27E-40	1.09523E-35	-0.272372705
Pin1	2.56E-38	3.85664E-34	-0.419157068
Macf1	1.17E-37	1.76261E-33	-0.317061243
Grb10	1.31E-37	1.97352E-33	-0.379438099
Wnt5b	4.74E-37	7.14081E-33	0.249084125
Mark1	9.67E-36	1.45679E-31	-0.264954223

Table S4. The differentially expression of Wnt related genes between epithelial and fibroblas

gene	p value	Adjusted p value	fold change *
Thra	4.08E-33	6.14652E-29	-0.341759039
Sfrp2	3.72E-32	5.60418E-28	-0.444073376
Wnt5a	4.15E-32	6.25198E-28	-0.229987584
Wisp2	6.85E-32	1.03195E-27	-0.278648466
Ror2	7.80E-32	1.17507E-27	-0.169814016
Amotl2	8.64E-32	1.30162E-27	-0.304828052
Cdc42	1.50E-31	2.25975E-27	-0.321796466
Fzd1	7.10E-31	1.06962E-26	-0.442886045
Nrarp	7.91E-31	1.19164E-26	0.22491565
Ift20	1.55E-30	2.33508E-26	-0.348695342
Ddit3	8.25E-30	1.24286E-25	-0.430943586
Fgf10	3.42E-29	5.15223E-25	-0.152134687
Fgf2	5.11E-29	7.69822E-25	-0.122412679
Ndrg2	1.18E-28	1.77767E-24	-0.192032154
Lats2	3.23E-28	4.866E-24	-0.195186234
Hoxb9	4.33E-27	6.52315E-23	-0.150486117
Frat2	1.24E-26	1.86806E-22	0.361014923
Pfdn5	2.43E-26	3.6608E-22	0.266938178
Cpe	2.36E-25	3.55534E-21	-0.331713893
Gli3	6.31E-25	9.50602E-21	-0.166624355
Fzd2	3.02E-23	4.54963E-19	-0.248391977
Aes	3.09E-23	4.65509E-19	0.223429383
Rbpj	1.44E-22	2.16936E-18	-0.223764532
Tbl1x	2.21E-22	3.32937E-18	-0.273468921
Itga3	2.29E-22	3.44989E-18	0.195630047
Mdfic	2.66E-22	4.00729E-18	-0.174773969
Fgfr2	4.24E-22	6.38756E-18	0.212687772
Wnt10a	5.36E-22	8.07484E-18	0.244819878
Cela1	8.15E-22	1.2278E-17	-0.126230919
Dact3	1.80E-21	2.7117E-17	-0.104079969
Tbx18	2.02E-21	3.04313E-17	-0.10092606
Tmem237	2.87E-21	4.32366E-17	-0.238283434
Mgat3	5.78E-21	8.70757E-17	0.23832028
Gpc3	1.69E-20	2.54599E-16	-0.214025262
Mdfi	7.81E-20	1.17658E-15	0.257787219
Siah2	1.36E-19	2.04884E-15	0.312883507
Bambi	3.68E-19	5.54392E-15	-0.296468378
Grk5	3.60E-18	5.4234E-14	-0.085501132
Prickle1	1.51E-17	2.27482E-13	-0.121539401
Foxo1	3.22E-17	4.85093E-13	0.274652345
Lrrk2	3.58E-17	5.39327E-13	-0.099864181

Table S4. The differentially expression of Wnt related genes between epithelial and fibroblas

gene	p value	Adjusted p value	fold change *
Jade1	5.89E-17	8.87329E-13	0.228514563
Notch1	6.38E-17	9.61147E-13	0.169478053
Wls	1.41E-16	2.12417E-12	0.241246489
Mesdc2	2.10E-16	3.16365E-12	-0.189623031
Myc	3.70E-16	5.57405E-12	-0.140662223
Tle1	1.00E-15	1.5065E-11	-0.217873228
Lef1	3.19E-15	4.80574E-11	-0.050941156
Mark2	1.91E-14	2.87742E-10	0.222153377
Ctnnd1	2.07E-14	3.11846E-10	0.254802347
Nfkb1	5.83E-14	8.7829E-10	-0.307350099
Dact1	6.12E-14	9.21978E-10	-0.085365
Sox9	6.19E-14	9.32524E-10	-0.205147347
Cby1	1.42E-13	2.13923E-09	-0.048461522
Leo1	2.57E-13	3.87171E-09	-0.160187699
Zranb1	5.44E-13	8.19536E-09	0.207734333
Wnt2	1.25E-12	1.88313E-08	-0.094587216
Hnf1b	1.65E-12	2.48573E-08	0.07483575
Frzb	3.32E-12	5.00158E-08	-0.06627257
Ptpru	9.16E-12	1.37995E-07	0.103105686
Mitf	1.74E-11	2.62131E-07	-0.05739222
Wdr61	1.97E-11	2.96781E-07	-0.081460082
Rnf43	1.01E-10	1.52157E-06	0.066628535
Bmp2	2.74E-10	4.12781E-06	-0.185847093
Cul3	2.86E-10	4.30859E-06	-0.131242655
Lzts2	3.07E-10	4.62496E-06	-0.163820725
Arl6	4.63E-10	6.9751E-06	0.147176807
Fgfr3	5.79E-10	8.72264E-06	0.084323394
Fzd6	7.15E-10	1.07715E-05	0.081749737
Rtf1	8.52E-10	1.28354E-05	0.167125558
Cpz	1.06E-09	1.59689E-05	-0.052267872
Csnk1a1	1.55E-09	2.33508E-05	0.169838207
Rnf146	1.71E-09	2.57612E-05	0.167324547
Brd7	3.14E-09	4.73041E-05	0.163507728
Dapk3	3.83E-09	5.7699E-05	-0.13469034
Yap1	4.87E-09	7.33666E-05	-0.141855645
Bcl7b	1.85E-08	0.000278703	-0.115950244
Scyl2	2.58E-08	0.000388677	0.120768975
Snai2	3.11E-08	0.000468522	0.062689869
Cthrc1	3.52E-08	0.000530288	-0.128516155
Dlx5	8.01E-08	0.001206707	0.056965971
Ctnnbip1	8.99E-08	0.001354344	0.036223499

Table S4. The differentially expression of Wnt related genes between epithelial and fibroblas

gene	p value	Adjusted p value	fold change *
Lrrfip2	9.23E-08	0.0013905	-0.105309299
Tmem64	9.52E-08	0.001434188	0.090315285
Cav1	1.47E-07	0.002214555	-0.051032733
Amer1	2.08E-07	0.00313352	-0.088446486
Znrf3	2.61E-07	0.003931965	0.070201801
Csnk2a1	2.80E-07	0.0042182	0.007946896
Csnk1e	2.82E-07	0.00424833	0.120188685
Ctnnb1	3.17E-07	0.004775605	-0.052174692
Pygo1	3.21E-07	0.004835865	-0.041827844
Lgr6	4.09E-07	0.006161585	0.043947399
Ccne1	4.37E-07	0.006583405	-0.057992969
Wwtr1	4.73E-07	0.007125745	-0.152225013
Tnks2	5.59E-07	0.008421335	0.037144848
Apc2	7.14E-07	0.01075641	-0.026729328
Sulf2	7.79E-07	0.011735635	-0.08311142
Nxn	1.11E-06	0.01672215	-0.082020971
Prkaa2	1.20E-06	0.018078	0.057890821
Pitx2	1.73E-06	0.02606245	-0.046251241
Grk6	1.86E-06	0.0280209	-0.060314905
Rac1	2.21E-06	0.03329365	-0.122547277
Hdac2	2.22E-06	0.0334443	0.094308498
Sdhaf2	2.27E-06	0.03419755	-0.050852087
Fzd9	2.82E-06	0.0424833	0.004458985
Zbed3	3.44E-06	1	-0.045022167
Ccny	3.68E-06	1	-0.003919382
Rapgef1	3.83E-06	1	-0.091136814
Stk11	3.94E-06	1	0.068838472
Csnk2b	4.05E-06	1	-0.114546653
Spin1	4.66E-06	1	-0.074678397
Tsc2	5.43E-06	1	-0.108050268
Csnk1d	6.16E-06	1	-0.05169917
Smarca4	6.84E-06	1	0.121338748
Rab5a	7.34E-06	1	-0.027589762
Fuz	8.47E-06	1	0.108637648
Stk3	1.17E-05	1	-0.084757707
Nkd2	1.20E-05	1	-0.089899039
Ptk7	1.42E-05	1	-0.134187373
Usp34	1.65E-05	1	0.076338795
Vps35	1.71E-05	1	-0.05744949
Porcn	1.83E-05	1	0.110977464
Emd	2.28E-05	1	0.021164651

Table S4. The differentially expression of Wnt related genes between epithelial and fibroblas

gene	p value	Adjusted p value	fold change *
Lgr4	2.37E-05	1	0.06642679
Cxxc4	2.58E-05	1	0.063717357
Fbxw4	2.86E-05	1	0.078385115
Fzd7	4.06E-05	1	-0.03730797
Ubr5	4.08E-05	1	-0.113145126
Tsku	4.21E-05	1	-0.020428155
Ankrd10	6.29E-05	1	0.101557441
Tlr2	6.57E-05	1	-0.055188942
Sall1	8.85E-05	1	0.025324497
Dixdc1	8.95E-05	1	-0.036681037
Tbl1xr1	9.54E-05	1	0.087626162
Sostdc1	0.000104	1	0.074587285
Abl2	0.00012059	1	0.074690087
Tcf7l1	0.00012906	1	0.093160312
Nle1	0.00014944	1	0.074386474
Wwox	0.00015859	1	0.089419296
Csnk1g3	0.00016839	1	0.023078542
Trpm4	0.00021108	1	0.063699468
Psen1	0.00022286	1	0.09829427
Vax2	0.00028417	1	-0.02386274
Mapk14	0.00031616	1	0.090239092
Otulin	0.00033122	1	0.024367665
Hdac1	0.00038486	1	-0.021267021
Nkx2-5	0.00042167	1	-0.019961361
Rspo1	0.00048441	1	0.022761686
Atp6ap2	0.0004851	1	-0.064110236
Caprin2	0.00068611	1	-0.038781858
Trabd2b	0.00092294	1	-0.015032806
Ube2b	0.00092569	1	0.086211341
Dvl2	0.00093744	1	-0.059385282
Sox4	0.00106926	1	-0.114822694
Abl1	0.0011186	1	-0.08366194
Prkaa1	0.00116852	1	-0.046110112
Fzd3	0.00120823	1	0.06236498
Sfrp4	0.0012433	1	-0.040209253
Amotl1	0.00145182	1	0.028986574
Mbd2	0.00159507	1	-0.073097285
Ctr9	0.00163659	1	0.096035546
Fbxw11	0.00170686	1	0.060969713
Draxin	0.00201063	1	-0.021057574
Gsc	0.00205909	1	-0.00483016

Table S4. The differentially expression of Wnt related genes between epithelial and fibroblas

gene	p value	Adjusted p value	fold change *
Pias4	0.00218513	1	0.102036716
Paf1	0.00276107	1	0.087900118
Limd1	0.0030196	1	0.035416968
Ddx3x	0.00343283	1	-0.013738181
Uty	0.00351923	1	0.048749741
Klf4	0.0037502	1	-0.102242394
Dvl1	0.00396404	1	-0.032856209
Gsk3b	0.00453161	1	0.063315946
Xiap	0.00463015	1	0.077914288
Tmem198	0.00483477	1	-0.03981833
Ilk	0.00503374	1	-0.076754831
Snx3	0.00549417	1	-0.027514186
Cdh3	0.00549621	1	0.029779749
Ankrd6	0.00618002	1	0.025651019
Chd8	0.00683122	1	0.087213955
Calcoco1	0.00786322	1	-0.022313222
Csnk2a2	0.00823044	1	-0.056982695
Zfp703	0.00891291	1	-0.065388706
Ror1	0.01008176	1	-0.016047226
Pygo2	0.0114283	1	0.010489079
Cdc73	0.01186762	1	0.048984726
Hbp1	0.01196868	1	0.064060667
Aspm	0.01392319	1	0.019374713
Ldb1	0.01475674	1	0.025900348
Naip2	0.0149579	1	0.0093315
Wnt7a	0.0149579	1	0.010225992
Fzd10	0.0149579	1	0.018959307
Shisa2	0.01591465	1	-0.017566202
Stk4	0.01612986	1	0.035509028
Tcf7	0.01791038	1	0.019966643
G3bp1	0.01823693	1	-0.030564453
Pten	0.01836483	1	-0.068320346
Med12	0.02111411	1	-0.04918339
Tax1bp3	0.02480969	1	0.00528615
Kdm6a	0.02828886	1	0.034115465
Hhex	0.02855046	1	0.023354833
Senp2	0.03008416	1	-0.055125294
Gli1	0.03034247	1	-0.001780326
Ppm1a	0.03332061	1	0.067439637
Fzd5	0.03349694	1	-0.011541053
Kank1	0.03438915	1	-0.017331951

Table S4. The differentially expression of Wnt related genes between epithelial and fibroblas

gene	p value	Adjusted p value	fold change *
Axin2	0.03946118	1	-0.022545858
Tnks	0.04031097	1	0.021434674
Sox7	0.04223134	1	-0.005385194
Tiam1	0.04315055	1	0.059861428
Arntl	0.04385047	1	0.007523399
Wnt9a	0.04923634	1	-0.01757598
Gsk3a	0.0513927	1	0.0621353
Eda	0.05249952	1	-0.021427623
Rnf138	0.05508831	1	0.048413618
Lrp6	0.06447433	1	-0.055066801
Lats1	0.07192667	1	0.018196299
Apc	0.07417088	1	0.058867228
Park2	0.08275677	1	-0.000390483
Nog	0.08280885	1	-0.02753193
Tcf7l2	0.08561023	1	0.060397479
Axin1	0.09408773	1	-0.054253646
Bcl9	0.09971873	1	0.05256694
Ccar2	0.10187454	1	0.027034351
Tmem88	0.10227135	1	0.001618228
Csnk1g1	0.11220255	1	0.01570548
Fzd8	0.12134457	1	-0.018023561
Ccdc88c	0.12167533	1	0.007841937
Sox17	0.12167533	1	0.008290613
Lgr5	0.12587765	1	0.015579089
Csnk1g2	0.12873887	1	0.029543061
Lrrk1	0.13133465	1	0.066591302
Wnt6	0.13364641	1	0.022724968
Invs	0.14069419	1	-0.026452533
Bcl9l	0.14451088	1	-0.015301848
Mad2l2	0.14851633	1	-0.023587458
Mllt3	0.15435939	1	0.06238386
Tle4	0.1636389	1	0.029045883
Celsr3	0.17913684	1	-0.005023602
Smad3	0.17961683	1	-0.045975293
Ccnd1	0.18020117	1	0.024563149
Fgf9	0.18204407	1	-0.004744227
Cyld	0.19226783	1	0.013097711
Six3	0.22462014	1	-0.005745769
Ctnnd2	0.23209037	1	-0.025029647
Nlk	0.23244543	1	-0.024732111
Rnf220	0.24037291	1	0.031606625

Table S4. The differentially expression of Wnt related genes between epithelial and fibroblas

gene	p value	Adjusted p value	fold change *
Lrp5	0.24358948	1	-0.004959042
Wnt10b	0.24817421	1	-0.009564426
Ctdnep1	0.26188105	1	0.004854009
Gata3	0.26193576	1	0.008821771
Klhl12	0.29226297	1	-0.00096131
Zbtb33	0.30782307	1	-0.007131993
Wnt16	0.30845578	1	0.010041711
Ppp2r3a	0.31126921	1	-0.018113983
Apcdd1	0.32446528	1	0.003419693
Foxo3	0.33247215	1	0.035262219
Amer2	0.35336161	1	-0.001736721
Lrp4	0.36546713	1	-0.0070059
Folr1	0.37635542	1	0.001534577
Rnf213	0.37677129	1	-0.012488378
Nphp3	0.38921731	1	-0.010558659
Dvl3	0.4564861	1	0.016164044
Vangl2	0.46983741	1	-0.012467268
Dab2ip	0.48847989	1	0.007697242
Nphp4	0.52024552	1	-0.000850854
Btrc	0.53256957	1	-0.002069145
Cdk14	0.54543235	1	0.036215784
Egf	0.54703377	1	0.005316349
D930015E06	0.56328183	1	0.020755905
Mks1	0.59298446	1	0.017604022
Src	0.60756981	1	0.022417092
Grhl3	0.63138451	1	0.010568625
Tle2	0.64850777	1	0.010373784
Fzd4	0.66754654	1	-0.002661444
Ift80	0.67951483	1	0.022842719
Notum	0.69389951	1	-0.014549358
Kremen1	0.76517881	1	0.009048712
Gata4	0.78845555	1	-0.011848359
Mcc	0.8468829	1	-0.000509576
Tnik	0.8504737	1	-0.004556618
Atp6v1c2	0.86500392	1	0.004461883
Atp6v0c	0.88047077	1	0.004966438
Wnt11	0.94485515	1	-0.001010819
Strn	0.95476963	1	0.006138881
Gprc5b	0.97324366	1	-0.006814223
Tle3	0.9774557	1	0.006577062
Wnt1	1	1	-0.005880309

Table S4. The differentially expression of Wnt related genes between epithelial and fibroblas

gene	p value	Adjusted p value	fold change *
Depdc1b	1	1	0.000884305
Naip5	1	1	0.011140092

* fold change for differentially expressed genes between epithelial cluster and fibroblast clus
